# CRISPR/Cas9-mediated deletion of *MADD* induces cell cycle arrest and apoptosis in anaplastic thyroid cancer cells

**DOI:** 10.1038/s41598-025-22907-1

**Published:** 2025-11-10

**Authors:** Velavan Bakthavachalam, Mark A. Sanborn, Manikannan Mathayan, Satyajeet S. Salunkhe, Jason M. Wood, Mark Maienschein-Cline, Suman Setty, Basirudeen Syed Ahamed Kabeer, Jalees Rehman, Bellur S. Prabhakar

**Affiliations:** 1 Scientist Jesse Brown VA Medical Center, Chicago, IL 60612 USA; 2 Department of Biochemistry and Molecular Genetics, University of Illinois College of Medicine, Chicago, IL 60607 USA; 3 Research Informatics Core, University of Illinois at Chicago, Chicago, IL 60612 USA; 4 Department of Pathology, University of Illinois College of Medicine, Chicago, IL 60612 USA; 5 Center for Global Health Research, Saveetha Medical College and Hospital, Saveetha Institute of Medical and Technical Sciences (SIMATS), Saveetha University, Chennai, India; 6 University of Illinois Cancer Center, Chicago, IL 60612 USA; 7 Department of Microbiology and Immunology, University of Illinois College of Medicine, Chicago, IL 60612 USA

**Keywords:** CRISPR/Cas9, MADD, Anaplastic thyroid cancer, Apoptosis, Cell cycle, Therapeutic target, Cancer, Cell biology, Oncology

## Abstract

Anaplastic thyroid cancer (ATC) is an aggressive malignancy with a poor prognosis and limited treatment options. Previous studies have shown that selective downregulation of the *MADD* (MAP-kinase-activating death domain-containing protein) gene isoform increases ATC cell susceptibility to TRAIL-induced apoptosis. However, the existence of multiple *MADD* gene isoforms raised the possibility of functional compensation. This study aimed to definitively evaluate the role of *MADD* in ATC by employing CRISPR-Cas9 to Cas9 to target exon 3, a conserved exon expressed in all known MADD isoforms, resulting in functional knockout of MADD expression. CRISPR-Cas9-mediated *MADD* knockout, performed in three ATC cell lines (8505 C, C643, and HTH7) with distinct mutational backgrounds, significantly impaired ATC cell function in vitro, as indicated by reduced viability, increased apoptosis, decreased migration, and G0/G1 cell cycle arrest. RNA-seq analysis revealed alterations in genes related to cell survival, proliferation, and metastasis. In the orthotopic ATC mouse model, *MADD* deletion dramatically suppressed tumor growth, reduced lung metastases, and prolonged survival. Our findings demonstrate that *MADD* plays a crucial role in ATC cell survival, proliferation, and metastasis. The consistent effects observed across multiple cell lines and in vivo models suggest that *MADD* may represent a promising therapeutic target for this aggressive malignancy.

**Supplementary Information:**

The online version contains supplementary material available at 10.1038/s41598-025-22907-1.

## Introduction

The incidence of thyroid cancer has been increasing worldwide over the past three decades^1^. In 2020, the age-standardized global thyroid cancer incidence was higher in females than in males (10.1 vs. 3.1 per 100,000 individuals, respectively)^2^. Thyroid cancers are categorized into three groups on the basis of the stage of differentiation: differentiated (follicular, oncocytic and papillary), poorly differentiated, and undifferentiated (anaplastic). This classification is determined by how closely the cancer cells resemble normal thyroid cells^3^. Anaplastic thyroid cancer (ATC) represents the most aggressive form among all types of thyroid cancers and is associated with a poor prognosis. ATC demonstrates rapid growth and widespread dissemination within the patient’s body^4^. Individuals diagnosed with ATC typically exhibit a median survival of 2–6 months^5,6^.

ATC typically displays resistance to conventional anticancer medications, including radioactive iodine^7^. Currently, several targeted therapies including tyrosine kinase inhibitors, CDK (Cyclin Dependent Kinase) 4/6 inhibitors, BRAF/MEK (Mitogen-activated protein kinase kinase) inhibitors, NTRK (Neurotrophic Tyrosine Receptor Kinase) inhibitors, and mTOR (mammalian target of rapamycin) inhibitors^8^ are undergoing clinical trials. While these therapies generally demonstrate some degree of anti-ATC efficacy, observations suggest a tendency for resistance to develop over time, possibly owing to the compensatory activation of overlapping prosurvival signaling pathways^5^. The complex mechanisms of ATC resistance underscore the importance of identifying potential new targets for developing novel therapeutic strategies that can overcome the challenges posed by the complex signaling networks involved. Advances in understanding these pathways and their interactions will contribute to the development of more effective and enduring targeted therapies for ATC^5,9^.

MAPK-activating death domain-containing protein (*MADD*), also known as Rab3aGTP-GDP exchange factor (RAB3GEP) or differentially expressed in normal and neoplastic cells (DENN) or insulinoma-glucagonoma clone 20 (IG20), is a ubiquitously expressed protein^10^ that regulates Rab3/Rab27 functions^11–16^, which in turn play critical roles in cell survival, vesicular trafficking and secretion^13,17–31^.

The *MADD* gene can encode multiple isoforms, each exhibiting a unique expression profile across various tissues^32^. Typically, multiple isoforms are expressed in different permutations and combinations in each cell type. Six different isoforms have been identified, and they arise from alternative splicing of exons 13, 16, 21, 26 and 34. These isoforms have varying effects on cell survival and function, with some promoting cell survival (such as *MADD* and KIAA0358) and others enhancing cell death (IG20pa and IG20-SV4)^10,33–42^.

Previous studies have investigated the role of *MADD* in cancer via RNA interference approaches to knockdown specific isoforms^33–37,40,43–47^. However, these studies were limited by the potential for compensation by other *MADD* isoforms. To overcome this limitation and comprehensively evaluate the role of *MADD* in ATC, we employed CRISPR-Cas9-mediated knockout targeting a conserved exon expressed in all known *MADD* isoforms.

We hypothesized that *MADD* deletion would impair ATC cell survival, proliferation, and metastatic potential. To test this hypothesis, we employed CRISPR-Cas9-mediated knockout of *MADD* in three ATC cell lines with distinct mutational backgrounds: 8505 C (BRAFV600E, TP53R248G, and TERT promoter C228T mutations); C643 (HRASQ61R, TP53R248Q, and TERT promoter C228T mutations); and HTH7 (NRASQ61R, TP53R273H, and TERT promoter C250T mutations).

We investigated the effects of *MADD* deletion on cell viability, apoptosis, migration, and cell cycle progression in vitro, as well as tumor growth and metastasis in an orthotopic mouse model of ATC. Additionally, we performed RNA sequencing to elucidate the transcriptomic changes associated with *MADD* deletion. This comprehensive approach aimed to provide a definitive perspective on the functional significance of *MADD* in ATC and inform potential therapeutic strategies targeting this gene.

## Methods

### Chemicals and reagents

4-Hydroxytamoxifen (4HT) was purchased from Sigma-Aldrich Chemical Company (St. Louis, MO, USA). HRP-conjugated Beta Actin Monoclonal antibody (Cat #HRP-60008) was acquired from Proteintech (Rosemont, IL USA) and used for direct detection in Western blots. For detection of other primary antibodies, in Western blotting, secondary anti-rabbit and anti-mouse Abs were purchased from Cell Signaling Technology (Danvers, MA, USA). The iCAS plasmid was procured from Addgene (#84232) (Watertown, MA USA). *MADD* sgRNA1&2 were procured from OriGene (Rockville, MD). The anti-MADD antibody (#A302-143 A) was from Bethyl Laboratories, sold by Thermofisher Scientific (Rockford, IL, USA), Annexin V-FITC (fluorescein isothiocyanate) kit, and Alexa Flour 488-conjugated goat anti-rabbit were purchased from Invitrogen (Thermo Fisher, IL, USA). DAPI (4′,6-Diamidino-2-phenylindole) was obtained from Sigma Aldrich (St. Louis, USA). Anti-Ki67 antibody [SP6] (Cat #ab16667) was procured from Abcam, and Anti-rabbit IgG HRP-linked antibody was procured from Cell Signaling Technology (Boston, USA).

### Cell lines

The human ATC cell lines 8505 C, C643 and HTH7 were procured from the University of Colorado Cancer Center, Aurora, CO, USA. All the cell lines were tested for mycoplasma and other pathogens before initiating experiments (MycoFluor™, Thermo Fisher Scientific, Inc) and were used at a low passage number (5–15). The cells were cultured in Roswell Park Memorial Institute (RPMI) 1640 media supplemented with 10% fetal bovine serum (FBS) and 1% antibiotic (penicillin and streptomycin)-antimycotic (Amphotericin B) (Gibco). The cells were incubated at 37 °C in a humidified CO_2_ incubator.

### Construction of CRISPR Cas9 for *MADD* deletion

**Fig. 1:**
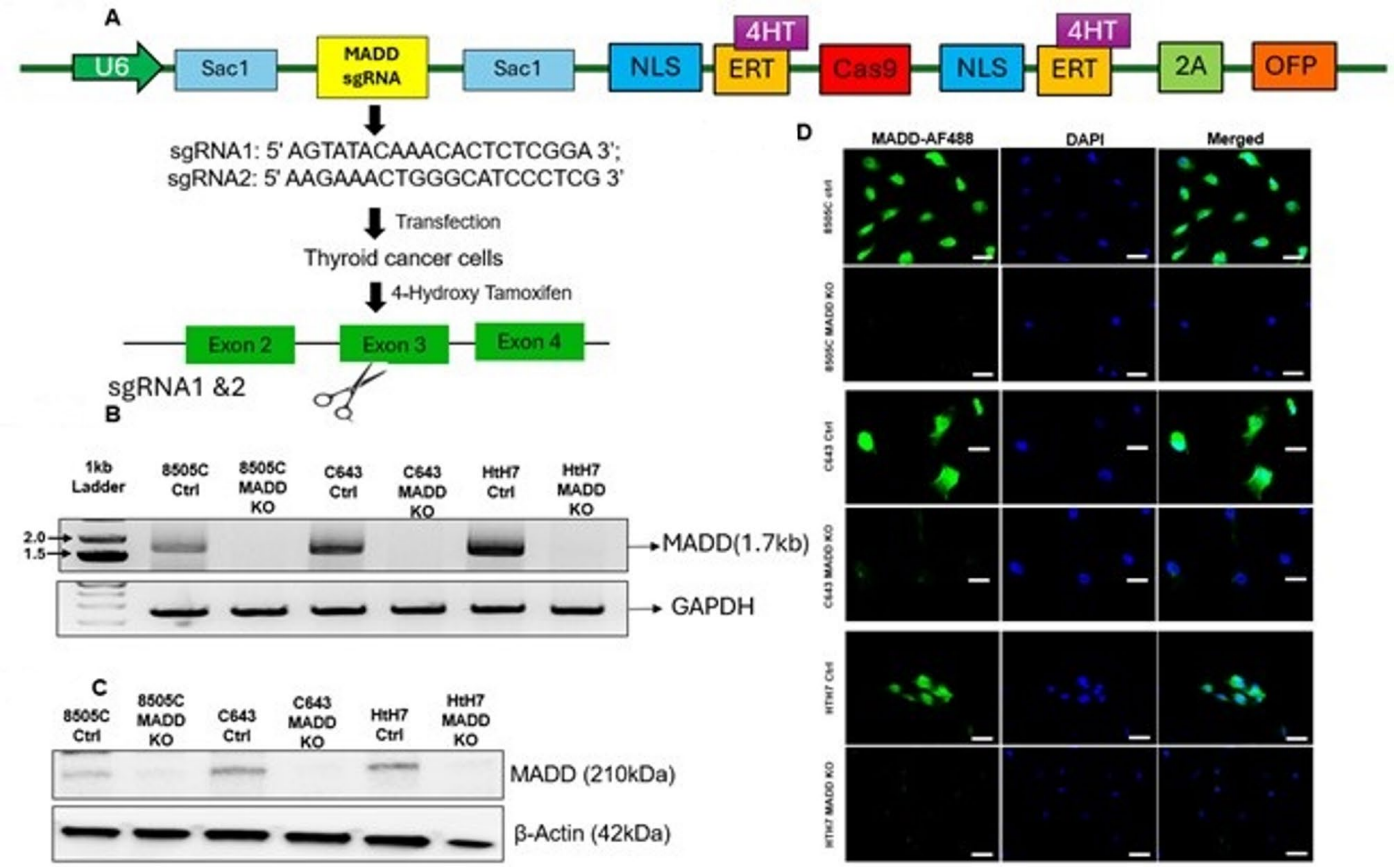
Construction and characterization of CRISPR/Cas9-mediated genomic knockout of *MADD* gene in ATC Cells: (**A**) Schematic diagram of the construction of CRISPR/Cas9-*MADD* dual-sgRNA targeting Exon 3 of the human *MADD* gene. (**B**) Confirmation of *MADD* Knockout by PCR compared to control cells. (**C**) Western blot analysis of MADD expression in control and knockout cells after 24 h of treatment with 4-Hydroxytamoxifen (4HT). β-actin served as a loading control. (**D**) Immunocytochemistry of *MADD* expression. Cells were cultured on sterile coverslips in the 8-well chamber, and following fixation, the cells were stained with anti-MADD-specific antibodies. DAPI staining was used for counter-staining. (Green = MADD, blue = nucleus) absence of any green color indicates that *MADD* was successfully knocked out in ATC cells at the translational level.

The CRISPR/Cas9 *MADD* single-guide RNA (sgRNA) targeting exon 3 of the human *MADD* gene was designed via the IDT online service: Predesigned Alt-R^®^CRISPR/Cas9 guide RNA (https://sg.idtdna.com/site/order/designtool/index/CRISPR_PREDESIGN). The sgRNA sequences used were sgRNA1 3’ AGTATACAAACACTCTCGGA 5’ and sgRNA2 5’ AAGAAACTGGGCATCCCTCG 3’ in chromosome 11 at site 748–767 and 810–829, respectively (For sequence-related information kindly refer to ‘Supplementary data - MADD Nucleotide seq – FASTA’). SgRNA1 has on target and off target scores of 89 and 70, respectively. The sgRNA2 on-target and off-target scores are 61 and 74, respectively. The negative control consisted of a nontargeting control sgRNA. The double-stranded guide sequence oligonucleotides were ligated into the Lenti-iCas plasmid (#84232, Adgene), which was linearized via the Sac 1 restriction enzyme. The Cas9 plasmid was constructed for editing activity with ERT (1 µM, 4HT) (Fig. 1A). We used an inducible Cas9 system that involves the Cas9 enzyme fused to a modified estrogen receptor (ERT) domain, which allows for temporal control of Cas9 activity. In this system, the Cas9-ERT fusion protein remains inactive in the cytoplasm until the addition of 4-hydroxytamoxifen (4HT), which binds to the ERT domain and induces translocation of the fusion protein into the nucleus where it can perform gene editing. This inducible approach was essential since our preliminary experiments showed that constitutive MADD deletion resulted in poor cell replication and eventual cell death after a few passages.

For plasmid control experiments, cells were transfected with the same Lenti-iCas plasmid containing a non-targeting control sgRNA sequence that has no complementary target in the human genome. These plasmid control cells underwent the same selection and 4HT treatment procedures as the MADD sgRNA-transfected cells, ensuring that any observed phenotypic changes in the MADD knockout cells were specifically due to MADD gene deletion rather than non-specific effects of plasmid transfection, selection pressure, or 4HT treatment.

### Transfections

The cells were seeded into 6-well plates at a density of 2 × 10^5^ cells/well and cultured in 2 ml of RPMI medium supplemented with containing 10% FBS without antibiotics for 24 h in a CO_2_ incubator. Lipofectamine 3000 (Invitrogen, Carlsbad, CA, USA) and sgRNA 1&2 and Cas9 plasmid DNA were separately diluted in 250 µl of portions of Opti-MEM reduced serum medium (Invitrogen). For transfection, we used the volume of DNA with two volumes of Lipofectamine-3000 (2 µL/µg DNA), or 5µg DNA/10µl Lipo 3000 for 6-well plate. After 5 minutes of incubation at room temperature, the diluted Lipofectamine 3000 and plasmid DNA were mixed. The DNA-liposome complex was incubated for 20–30 min at room temperature and then directly added dropwise to the cultured cells in 2 ml of RPMI medium. The culture medium was replaced with RPMI medium containing 10% FBS with antibiotics after transfection for 24 h in a CO_2_ incubator. The efficiency of transfected cells was detected by fluorescence microscopy using the OFP channel after 48 h of incubation. The transfected cells were then sorted using a cell sorter (MoFlo Astrios EQ, Beckman) at 570 nm to isolate the successfully transfected OFP + cell population. The sorted cells were subsequently treated with 1 µM 4HT for 24 h to induce *MADD* gene knockout.

### Cell proliferation

Cell proliferation was assessed using the MTS (3-(4,5-dimethylthiazol-2-yl)-5-(3-carboxymethoxyphenyl)-2-(4-sulfophenyl)-2 H-tetrazolium) assay. Equal numbers of nontransfected control, negative sgRNA-transfected, 4HT control, and *MADD* sgRNA1&2-transfected cells were plated and incubated at 37 °C in a CO_2_ incubator for 24, 48 and 72 h. For the assay (MTS), we used 10,000 cells/well in the 96-well plate. At each time point, MTS was added to the plate, followed by incubation for 2 h at 37 °C in a CO_2_ incubator. Absorbance was recorded at 590 nm using an ELISA plate reader.

### Apoptosis

Apoptosis was detected by flow cytometry using an Annexin V-FITC/DAPI apoptosis detection kit (Invitrogen, USA). The cells were seeded into a 6-well plate at 2 × 10^5^ cells/well and incubated for 72 h. The cells were then collected, washed twice with ice-cold PBS, and resuspended in 500 µl of binding buffer. Annexin V/FITC (5 µl) and (10 µg/ml) 5 µl of DAPI were sequentially added to the cell suspension, which was subsequently incubated at room temperature in the dark for 15 min. The cells were analyzed on a flow cytometer (CytoFLEX S, Beckman, USA). Annexin V/FITC-positive and DAPI-negative cells were scored as apoptotic. The data were analyzed by Kaluza 2.1 software.

### Cell cycle analysis

ATC cells (1 × 10^6^) were plated in 6-well plates and incubated at 37 °C. After 24 h, the cells were treated or left untreated with 4HT. After 72 h, both live and dead cells were collected and centrifuged for 10 min at 1000 rpm. The cells were fixed with 70% ice cold ethanol at -20 °C overnight. Then, the ethanol was removed, and the cells were resuspended in 200 µl of RNase, stained with 10 µg/ml DAPI for 30 min at room temperature, and analyzed using flow cytometry (CytoFLEX S, Beckman, USA). The data were analyzed by Kaluza 2.1 software.

### Colony formation assay

The effect of *MADD* gene deletion on cellular clonogenicity was determined by a colony formation assay. 500 cells/well were plated in 6-well plates and incubated at 37 °C for 24 h. *MADD* sgRNA transfected, and drug control cells were treated with 4HT for 2 weeks, then fixed with acetic acid: methanol (1:7) and stained with 0.1% crystal violet. After washing, the colonies were counted, and images were captured with the Keyence microscope.

### Trans-well assays

Trans-well assay was used to evaluate the ability of the cells to migrate, as described previously^44^. In brief, the 100 µl of cell suspension (1 × 10^4^) was added to the upper chamber of the Boyden chamber, and 800 µl of medium containing 10% FBS containing medium was to the lower chamber. The cells were allowed to migrate for 24 h, and the trans-well chamber was washed with PBS and then fixed with 4% paraformaldehyde for 10 min. The fixed cells were washed with PBS and then stained with 0.1% crystal violet for 30 min. After washing, the cells were imaged from different areas under a microscope (Keyence), and the migrated cells were counted. The percentage of migrated cells was determined by dividing the number of cells in the lower chamber by the total number of unmigrated cells in the upper chamber.

### PCR

The DNA was extracted from the cells with DNeasy kit (Qiagen, USA) according to the manufacturer’s instructions, and the quantity and purity of the DNA were measured by Nanodrop (Thermo Scientific). PCR (polymerase chain reaction) was performed using Q5 high-fidelity DNA polymerase (M0491) New England Biolab according to the manufacturer’s instructions. The total volume of the reaction solution was 50 µl. The preparation was subjected to 34 cycles of PCR (denaturation at 95 °C for 30 s, annealing at 62 °C for 30 s, and extension at 72 °C for 2 min). GAPDH primers were used as an internal control, and the PCR products were visualized on a 1% agarose gel. The sequences of the primers used for *MADD* were as follows: Forward: 5′-TCACGCGATATGGCATCTGT-3′, *MADD* Reverse: 5′-TCCTAGTTCTTGTACTGCCCA-3′, GAPDH Forward: 5′-TGTGGGCATCAATGGATTTGG-3′ and GAPDH Reverse: 5′-ACACCATGTATTCCGGGTCAAT-3′.

### Western blot

Briefly, 50 µg of whole-cell lysates from the nontransfected control, negative sgRNA-transfected and *MADD* gene knockout cells were subjected to 4–20% gradient SDS-PAGE, transferred on to the PVDF membrane, blocked with 5% skimmed nonfat milk and incubated with different primary antibodies diluted in 1% bovine serum albumin in Tris-buffered saline (1% BSA in TBS) at 4 °C overnight on a rocker shaker. After being washed with 0.1% TBST, the membrane was incubated with the corresponding HRP conjugated secondary antibody, anti-mouse IgG, which was prepared in blocking solution (1% milk in 0.1% TBST), for one hour at room temperature. After washing with 0.1% TBST, the blots were developed after Pierce enhanced chemiluminescence (ECL) western blotting substrate (Thermo Fisher Scientific) was added. Densitometric values were analyzed using Image J software.

### Immunocytochemistry

The cells were allowed to grow in 8-well chambers to attain 70–80% confluency and fixed with 4% paraformaldehyde (pH 7.4) for 10 min at room temperature. After that, the cells were permeabilized with 0.05% Triton X-100 (Sigma-Aldrich), washed with TBST (0.1% Tween 20 in TBS), and blocked with 5% BSA solution. The cells were incubated with the rabbit anti-MADD primary antibody (1:100) (Bethyl Laboratories) overnight at 4 °C, washed with TBST, and incubated with anti-rabbit AF488 (1:200) secondary antibody. After washing, the cell nuclei were stained with DAPI solution, and coverslips were placed onto the slides with mounting medium (Thermo Fisher Scientific). Images were captured using a Keyence microscope at 20X magnification.

### Immunohistochemistry of mouse tissues

Following the fixation in 10% formalin, the mouse tumor samples were loaded into ASP 300s automated tissue processor (Leica Biosystems) and dehydrated in a series of ascending graded ethanol solutions, cleared in xylene, and infused with paraffin following a preset protocol. The samples were then embedded in paraffin and sectioned (5 μm). The sections were mounted on positively charged slides, dried, and baked at 60 °C for an hour. The adhered sections were deparaffinized on Leica Autostainer XL automated stainer (Leica Biosystems) using xylene and descending ethanol series, rehydrated in tap water, and stained with hematoxylin (Vintage Hematoxylin, StatLab#SL100) and eosin (Vintage Eosin, StatLab #SL101) using a preset protocol on the Autostainer XL. Staining was performed with BOND Polymer Refine Detection Kit (Leica, #DS9800) on BOND RX automated stainer (Leica Biosystems) according to the preset protocol.

A subset of slides was used for immunohistochemistry. After deparaffinization, the sections were subjected to heat-based antigen retrieval with BOND Epitope retrieval buffer 1 (pH 6.0, Leica Biosystems, #AR9961) for 40 min at 99 °C. Endogenous peroxidase activity and nonspecific binding were blocked by sequentially treating samples with peroxidase block (BOND Polymer Refine Detection Kit) and protein block (Background Sniper, Biocare Medical, #BS966) for 15 min at room temperature. The sections were then incubated with Ki67 antibody (1:200, ab16667, Abcam) for 30 min. After several washes, the signal detection was performed with anti-rabbit-Poly-HRP and 3,3′-diaminobenzidine (DAB) from the BOND Polymer Refine Kit by incubating the sections for 15 min and 10 min at room temperature. All the sections were counterstained with hematoxylin. The slides were dehydrated on Autostainer XL and mounted with Micromount media (Leica Biosystems) and imaged with a Keyence microscope set to phase contrast/brightfield magnification (200x).

### Orthotopic thyroid cancer model

To generate tumor orthotopic xenografts, athymic nude mice were procured from Jackson Laboratories and maintained in a pathogen-free facility of the biological resources laboratory (BRL) at the University of Illinois at Chicago (UIC, Chicago, IL). All animal experiments were approved by the Animal Care and Use Committees at the University of Illinois at Chicago (UIC) and Jesse Brown VA Medical Center, Chicago, IL. The experiments were performed in accordance with the guidelines and regulations set forth by these committees. Furthermore, all procedures were carried out in compliance with the ARRIVE guidelines.

To generate orthotopic xenografts, the mice were anesthetized with an i.p injection mixture of 100 mg /kg ketamine and 5 mg/kg xylazine in sterile saline. The neck was scrubbed with a surgical scrub and then with an alcohol pad. A one-centimeter midline cervical incision was made, and then the strap muscle was retracted laterally to visualize the thyroid gland. Under the surgical microscope, 1 × 10^6^ 8505 C control cells (groups 1 & 2), 8505 C plasmid control cells (group 3), and 8505 C sgRNA-*MADD*-transfected cells (group 4) (*n* = 6) suspended in 5 µl of serum-free RPMI medium were directly injected into the right thyroid gland using a Hamilton gas-tight syringe and 30G hypodermic needle. Buprenorphine (0.1 mg/kg SC) was given as postoperative analgesia prior to recovery from anesthesia. The mice were monitored daily for signs of infection and weight loss. After 17–28 days, the tumors were detected in the control, drug control, and plasmid control groups, and 106–121 days were detected in the *MADD* gene knockout groups. On days 28–35, the tumor volume reached approximately 0.4–0.8 cm. Group 1 mice were treated with corn oil, and groups 2&3 were treated with 20 mg/ml of 4HT on alternative days for 10 days (to delete the *MADD* gene). The mice were sacrificed by CO2 inhalation followed by decapitation when weight loss exceeded 20% of their body weight or at the end of the observation period, and the tumors from the thyroid and lung tissues were collected. The tissues were fixed in formalin, processed into paraffin, sectioned, and subjected to hematoxylin and eosin (H&E) staining and immunohistochemistry for Ki-67 and the stained sections were evaluated by light microscopy (representative images are presented in Fig. 5G and H).

### RNA-sequencing (RNA-seq) and data collection

Total RNA was extracted and purified using the Promega Maxwell RSC simple RNA kit (Cat.no. ASI390). The RNA and DNA contents of the RNA samples were quantified using Qubit fluorimeter and HS RNA assay (Invitrogen), and their integrity was analysed using Agilent 4200 TapeStation with RNA ScreenTape. The levels of remaining DNA did not exceed 15–17% of the total amount of nucleic acid.

Libraries for Illumina sequencing were prepared in one batch in a 96-well plate using CORALL Total RNA-seq Library Prep Kit with Unique Dual Indices with the RiboCop HMRv2 rRNA Depletion Kit (Lexogen). In brief, approximately 250 ng of total RNA per sample was used for the first rRNA depletion step, followed by library generation initiated with random oligonucleotide primer hybridization and reverse transcription. Next, the 3’ ends of first-strand cDNA fragments were ligated with a linker containing Illumina-compatible P5 sequences and unique molecular identifiers (UMIs). During the following steps of second strand cDNA synthesis and ds cDNA amplification, i7 and i5 indices as well as the complete adapter sequences required for cluster generation were added. The number of PCR amplification cycles was 11 as determined by qPCR using a small preamplification library aliquot for each sample.

The final amplified libraries were purified and quantified, and the average fragment sizes were confirmed to be approximately 380–405 bp by gel electrophoresis using 4200 TapeStation and D5000 Screen Tape (Agilent). The concentration of the final library pool was confirmed by qPCR (quantitative PCR), and the pool was subjected to test sequencing on MiniSeq instrument (Illumina) to check the sequencing efficiency and adjust the proportions of individual libraries accordingly. Sequencing was carried out on NovaSeq X 10B (Illumina) with 2 × 150 bp reads. Sequencing was performed at the Roy J. Carver Biotechnology Center at the University of Illinois at Urbana-Champaign.

### Gene expression quantification

FastQC version 0.11.9 was used to assess the sequence quality of the raw RNA-seq data. The reads were quantified against the Ensembl GRCh38 human transcriptome (release 109) using Salmon (v1.10.1)^48^. Gene level counts were generated by collapsing individual transcript counts.

### Differential expression analysis

Differential expression analysis was done using DE-Seq2 method^49^. High-level sample clustering was assessed using principal component analysis (PCA), and experimental variability was estimated using the biological coefficient of variation (BCV). Pair-wise comparisons between sample groups were performed to analyze differences between the control and *MADD* deletion groups for each cell line. Multigroup statistics were performed to test for effects from the cell line, genotype, and interactions between the cell line and the genotype. All p-values were adjusted using the false discovery rate (FDR) correction of Benjamini and Hochberg. Significantly differentially expressed genes were determined on the basis of an FDR threshold of 0.05 and |log2FC| >0.5.

### Pathway enrichment analysis

Differentially expressed genes were subjected to pathway and gene ontology enrichment analyses using GSEApy^50^ against the GO biological processes and KEGG databases. All genes detected in the assay were used as the background.

### Statistical analysis

All the data are presented as the means ± SD. Differences between groups were evaluated with ANOVA and Dunnett’s multiple comparisons test. Statistical analysis was performed with GraphPad Prism 7, and a *p* < 0.05 was considered statistically significant.

## Results

### Construction and characterization of CRISPR/Cas9-mediated *MADD* gene knockout in ATC cells

To investigate the role of *MADD* in ATC, we employed CRISPR/Cas9-mediated gene editing to delete the *MADD* gene in three different ATC cell lines with different mutational backgrounds: 8505 C (Braf, p53, and TERT mutated), C643 (HRas, p53, and TERT mutated), and HTH7 (NRas, p53, and TERT mutated)^41^. The schematic design of CRISPR Cas9 is shown in Fig. 1A (Supplementary Fig. 1). Transfection with sgRNAs resulted in efficient *MADD* knockout, as confirmed by PCR (Fig. 1B, Supplementary Fig. 2A-2B), Western blotting (Fig. 1C, Supplementary Fig. 2C-2D), and immunofluorescence staining (Fig. 1D). The PCR analysis showed successful deletion of the targeted region in *MADD* knockout cells compared with control cells. Western blot analysis revealed a complete loss of MADD protein expression in the knockout cells, which was further substantiated by the absence of immunofluorescence signal. Notably, we initially employed the CRISPR-Cas9 method without an inducible promoter to delete the *MADD* gene in ATC cells. However, the ATC cells with *MADD* gene deletion replicated very poorly and eventually died after a few passages. We subsequently utilized 4HT-dependent conditionally activated Cas9 to induce the deletion of the *MADD* gene.

### Level of *MADD* gene expression predicts ATC prognosis

**Fig. 2:**
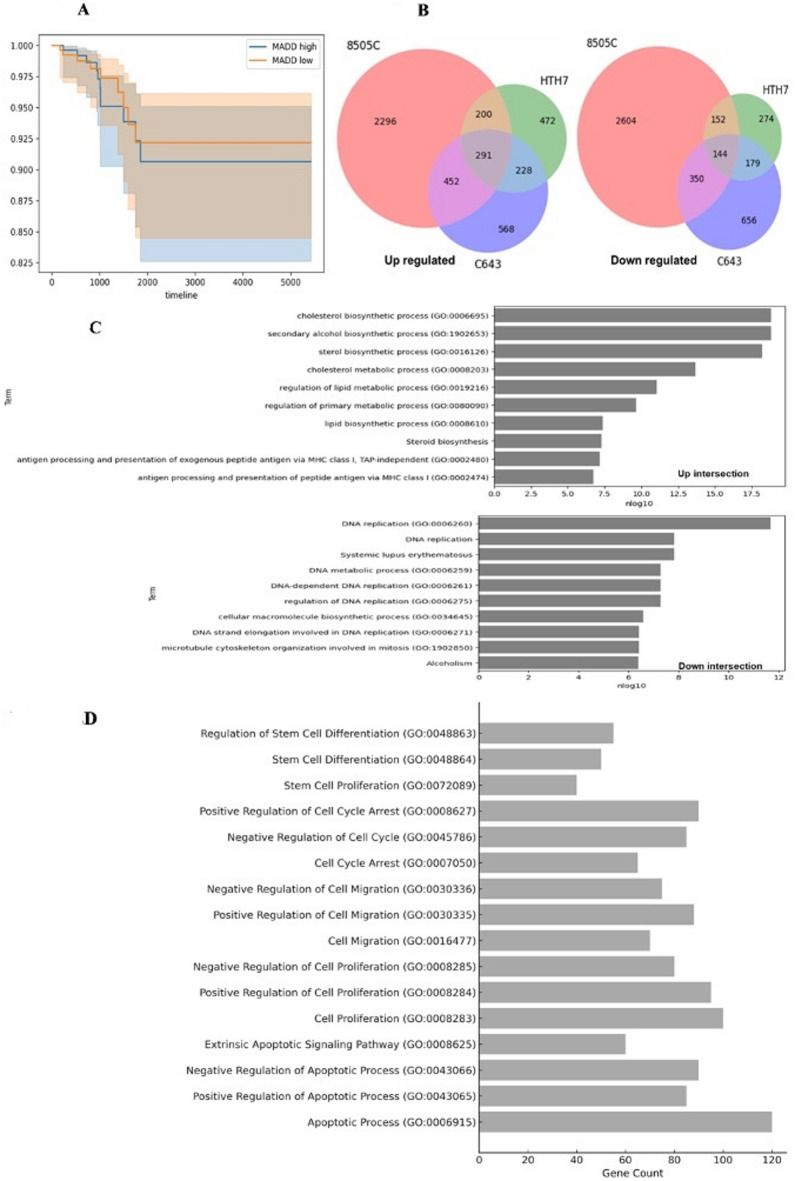
*MADD* gene deletion leads to transcriptomic changes in ATC cell lines: (**A**) Kaplan-Meier curve demonstrates the association of *MADD* mRNA expression levels in thyroid cancer patients with the overall survival rate in the TCGA database. High *MADD* expression correlates with shorter overall survival Shaded areas represent 95% confidence intervals, timeline in days (X-axis). (**B**) Venn diagrams showing the overlap of up-regulated (left) and down-regulated (right) genes among 8505 C, C643, and HTH7 cell lines following *MADD* deletion. (**C**) Gene Ontology (GO) based enrichment analysis of differentially expressed genes (DEGs) of *MADD* knockout and control cells. (**D**) GO enrichment analysis of differentially expressed genes related to cell cycle, proliferation, migration, and apoptosis. The bar graph shows the gene count for each GO term.

To assess the clinical relevance of *MADD* expression in thyroid cancer, we analyzed RNA-seq data from 564 thyroid cancer patients (primarily papillary thyroid cancer patients) from The Cancer Genome Atlas (TCGA) dataset^51^. Kaplan-Meier analysis revealed that patients with higher *MADD* mRNA levels (above the median) had significantly shorter overall survival than those with lower MADD mRNA levels (Fig. 2A). While these data are derived primarily from differentiated thyroid cancers due to the rarity of ATC, they suggest that *MADD* may serve as a prognostic biomarker in thyroid malignancies. The association of high *MADD* expression with poor prognosis underscores its potential importance in thyroid cancer biology and progression.

### *MADD* gene deletion leads to transcriptomic changes in ATC cell lines

To gain insight into the molecular pathways affected by *MADD*, we performed RNA-seq on the ATC cell lines with or without *MADD* deletion. Differential expression analysis identified 6,489, 2,868, and 1,940 genes that were significantly up- or down-regulated (FDR < 0.05, |log2FC| > 0.5) in 8505 C, C643, and HTH7 cells, respectively (Fig. 2B). Gene Ontology (GO) based enrichment analysis of differentially expressed genes (DEGs) in *MADD* gene-deleted and control cells (Fig. 2C) was also performed. Our analysis revealed differential regulation of genes associated with various critical physiological functions, including cell cycle regulation, cellular biosynthesis processes, and DNA replication. To focus on biological processes known to be involved in cancer progression, we filtered the GO terms related to cell proliferation, migration, and apoptosis (Fig. 2D). We found several genes related to apoptosis, metastasis, proliferation, and cell cycle arrest that were significantly impacted upon *MADD* deletion. These transcriptomic changes suggest that *MADD* deletion alters key cancer-related pathways in ATC cells, providing a mechanistic basis for its functional effects.

### *MADD* gene deletion induced spontaneous cell death in ATC cell lines

Given the transcriptomic changes observed, we investigated the functional consequences of *MADD* deletion in ATC cells. First, we assessed the impact of *MADD* gene deletion on induced *MADD* gene deletion in ATC cell lines. To rule out the direct cytotoxic effect of 4HT treatment in control and *MADD* gene knockout cells, we treated the control cells with different concentrations (0.1 to 2 µM) of 4HT for 0–72 h. We found that more than 80% of the control cells were viable after treatment with 1µM 4HT for 72 h, indicating minimal nonspecific toxicity. This finding was further confirmed by Annexin V/PI staining, which showed greater than 90% viability in control cells treated with 1 µM 4HT for 72 h(data not shown). Based on these results, 4HT doses ranging from 0.5 to 1 µM were used in subsequent experiments.

**Fig. 3:**
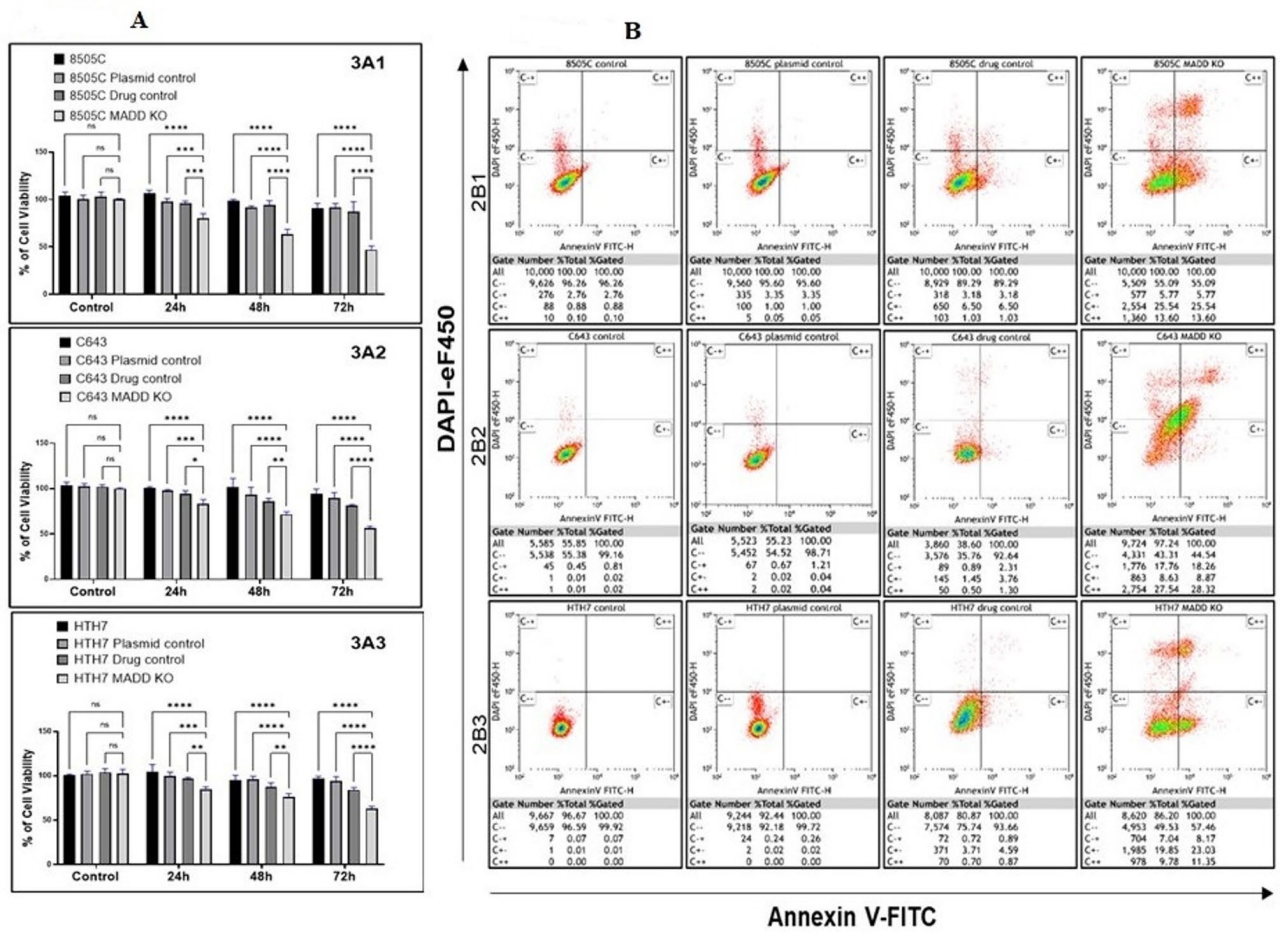
*MADD gene* knockout induces spontaneous cell death in thyroid cancer cells: (**A**) Cell viability (MTS) assay of 8505 C (3A1), C643 (3A2) and HTH7 (3A3) cells at 24, 48, and 72 h post treatment. Control, plasmid control, and *MADD* knockout cells were cultured with or without 4HT. Data expressed as mean ± SD (three replicates). *p < 0.01, **p < 0.0011, ***p < 0.0009 and ****p < 0.0001 level by Two-way ANOVA, Dunnett’s multiple comparisons test. (**B**) Apoptosis analysis of 8505 C (3B1), C643 (3B2), and HTH7 (3B3) cells by Annexin V-FITC/DAPI staining and flow cytometry at 72 h post-treatment. Representative plots shown for each cell line. Groups are as follows Control: Cells with no treatment (blank); Plasmid control: Plasmid without sgRNA, no treatment; Drug control: Cells treated with 4-Hydroxy Tamoxifen (1-micromolar); MADD KO : Plasmid with MADD sgRNA treated with 4-Hydroxy Tamoxifen (1 Micromolar); (**A**) Cell viability study using MTS; (**B**) Apoptosis study using Annexin-V/DAPI. Bar graphs indicate percentage of apoptotic cells. Data expressed as mean ± SD (*n* = 3). ****p < 0.0001 by one-way ANOVA with Dunnett’s test.

Next, we assessed the effects of *MADD* deletion on cell viability using MTS assays. *MADD* knockout significantly reduced viability in all three cell lines at 24, 48, and 72 h post-deletion compared to controls (Fig. 3A). This reduction in viability was accompanied by a marked increase in apoptosis, as determined by Annexin V/PI staining and flow cytometry. The percentage of apoptotic cells at 72 h was significantly higher in *MADD* knockout cells than in control cells (44.91% vs. 3.76% in 8505 C, 55.45% vs. ≤1% in C643, and 42.45% vs. ≤1% in HTH7; *p* < 0.001, Fig. 3B). Notably, 4HT treatment alone induced apoptosis in < 8% of the cells, confirming that the observed effects were due to *MADD* deletion.

### *MADD* gene deletion decreased clonogenicity of ATC cells

**Fig. 4:**
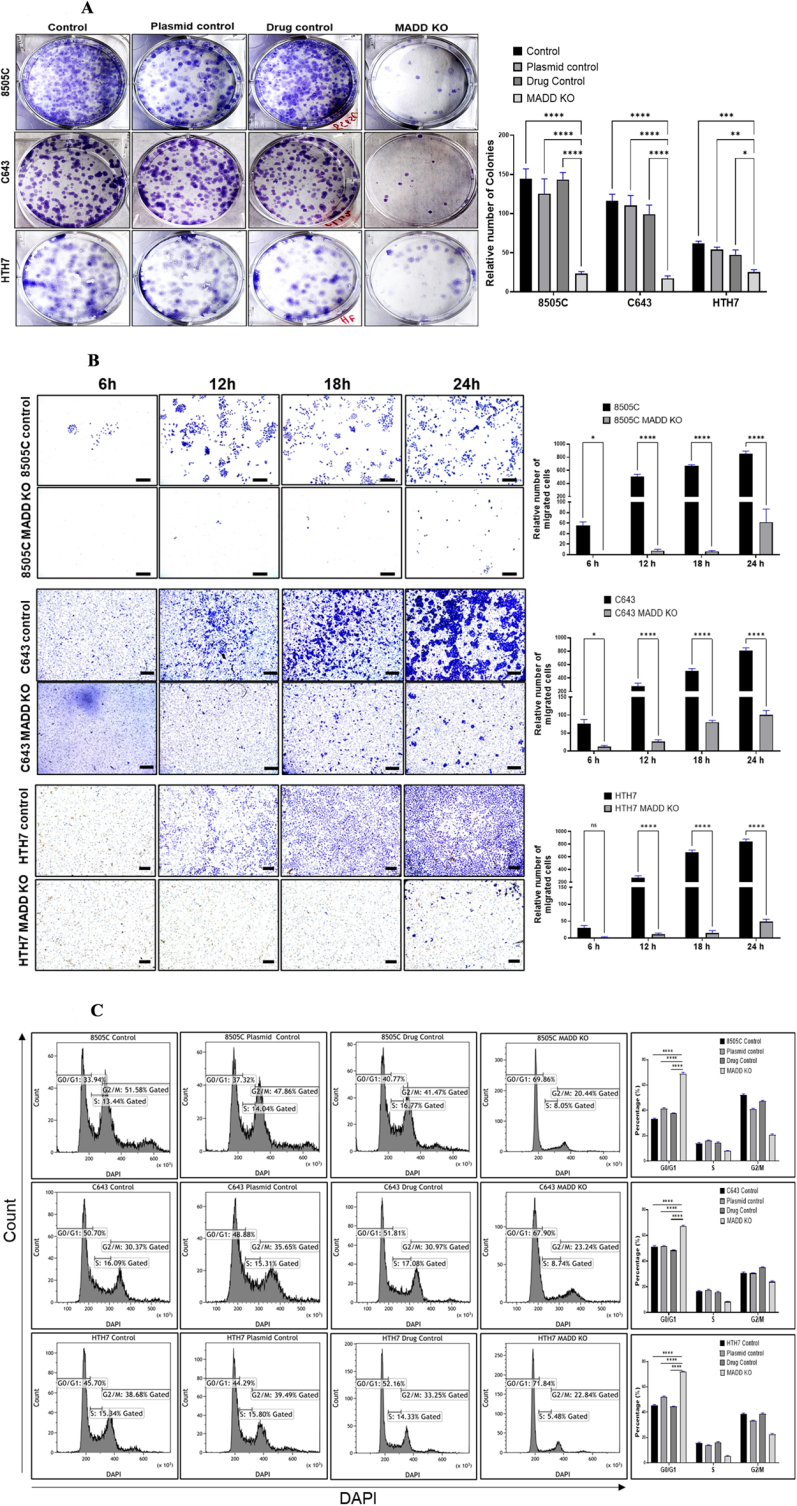
*MADD* gene deletion inhibits migration, clonogenicity, and induces cell cycle arrest in ATC cell: (**A**) Trans-well migration assay. Boyden chamber-based trans-well-insert assay showed a significant suppression in the percentage of migrated cells in the lower chamber upon *MADD* knockout in thyroid cancer cells as compared to control cells in vitro. Three independent experiments for each sample were carried out. *p < 0.03, ****p < 0.0001 level by Two-way ANOVA, Dunnett’s multiple comparisons test. (**B**) Colony formation assay. The colony formation ability of the *MADD* knockout cells was examined by the plate colony formation assay. In each group 500 cells/well were seeded in 6 wells to form colonies for up to 2 weeks. The results showed that the colony forming ability of the *MADD* knockout cells was significantly suppressed as compared to all control cells. *p < 0.02, **p < 0.003, ***p < 0.0002 and ****p < 0.0001 level by Two-way ANOVA, Dunnett’s multiple comparisons test. (**C**) Cell cycle analysis by DAPI staining and flow cytometry. Data shows that cell cycle arrest at G0/G1 phases in *MADD* knockout ATC cells. In 8505 C, C643, and HTH7 cells showed a significant G0/G1 phase arrest against *MADD* knockout as compared to control, plasmid, and drug control cells. Therefore, *MADD* deletion can induce cell cycle arrest in ATC cells at G0/G1 phases. ****p < 0.0001 level by two-way ANOVA, Dunnett’s multiple comparisons test.

To assess the impact of *MADD* deletion on long-term growth potential, we performed colony formation assays. Equal numbers of control and *MADD* knockout cells (500/well) were seeded and cultured with or without 0.5 µM 4HT for 1–3 weeks. *MADD* knockout significantly reduced the clonogenic ability of all three ATC cell lines compared to controls (*p* < 0.01, Fig. 4A), with fewer and smaller colonies formed over the observation period. Crystal violet staining and quantification confirmed the substantial reduction in colony formation upon *MADD* deletion.

### *MADD* gene deletion decreased the migration of ATC cells

Given that ATC cells are highly invasive, we next investigated the effect of *MADD* deletion on cell migration using transwell assays. Equal numbers of control and *MADD* knockout cells (1 × 10^4^ in 100 µL serum-free medium) were seeded into the upper chamber of transwell inserts, with 800 µL of 10% FBS-containing medium in the lower chamber. After 6, 12, and 24 h, the migrated cells were fixed, stained with crystal violet, and counted. *MADD* knockout significantly decreased the migratory capacity of 8505 C, C643, and HTH7 cells at all time points compared to controls (*p* < 0.01, Fig. 4B). The percentage of migrated cells was calculated by dividing the number of cells in the lower chamber by the total cell number of cells. These results suggest that *MADD* may contribute to the metastatic potential of ATC cells.

### *MADD* gene induced cell cycle arrest in ATC cells

To further understand the anti-proliferative effects of *MADD* deletion, we analyzed the cell cycle distribution by flow cytometry. Control and *MADD* knockout cells (1 × 10^6^) were fixed, stained with DAPI, and analyzed for DNA content. In all three cell lines, *MADD* knockout led to a significant increase in the percentage of cells in the G0/G1 phase and a corresponding decrease in the S and G2/M phases (*p* < 0.01, Fig. 4C). The G0/G1 population increased from 30.66% to 69.10% in 8505 C, from 51.11% to 65.44% in C643, and from 38.95% to 70.56% in HTH7 cells upon *MADD* deletion. These results indicate that *MADD* deletion induces cell cycle arrest, which is consistent with the observed changes in proliferation and clonogenicity.

### *MADD* gene deletion diminishes ATC Cell growth in athymic nude mice

**Fig. 5:**
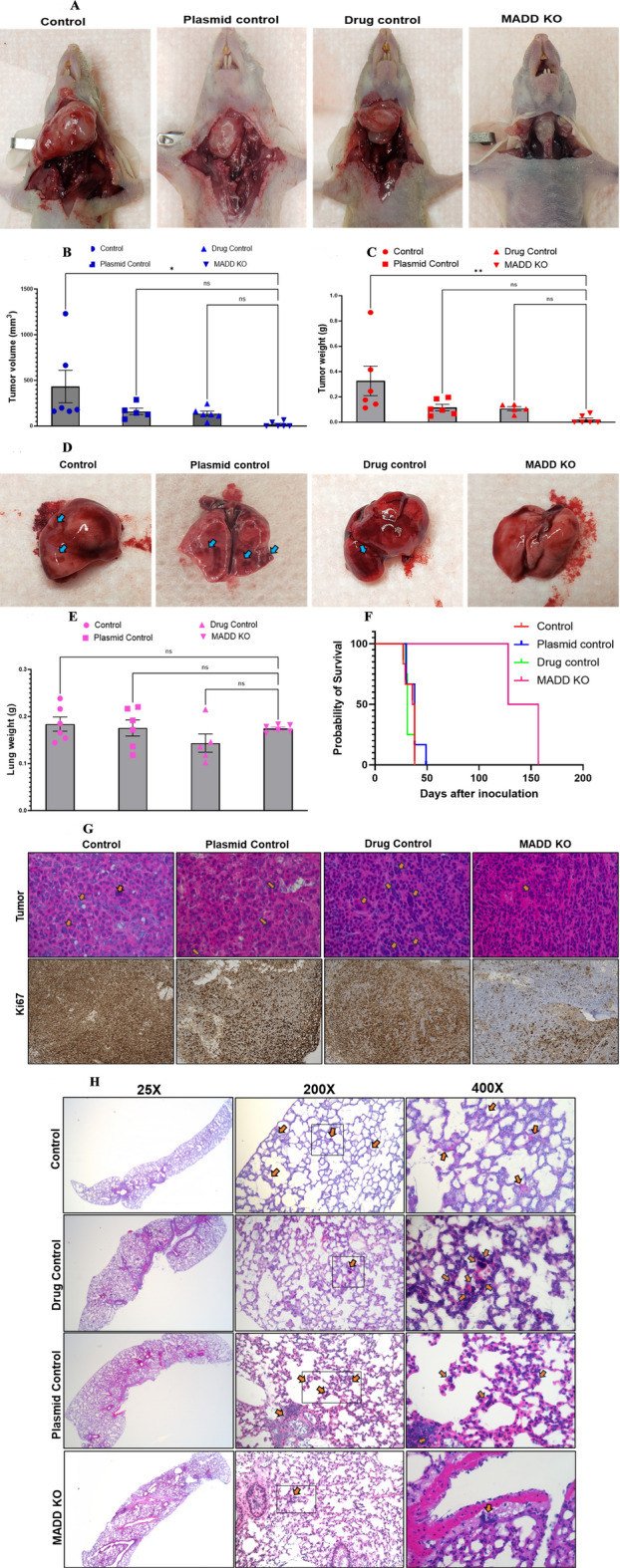
*MADD* knockout abrogates ATC cell growth and metastases in athymic nude mice. (**A**) Representative photograph of 8505 C orthotopic tumor-bearing mice demonstrating variation in tumor size. (**B** and** C**) Tumor volume and weight over time at the study endpoint. After 10 days of tumor notice, or the weight drops to 20% below its original baseline weight. (**D** and** E**) Representative images of lung specimens. (**F**) The Kaplan-Meier survival curves illustrate the survival probability (%) for the four mice groups. Tumor formation and mortality were significantly delayed in *MADD* knockout mice. (**G**) Representative H&E and Ki-67 immunohistochemistry-stained sections of thyroid tumors in mice. Ki-67 staining tumor nuclei were reduced in thyroid tumors induced by *MADD* knockout 8505 C cells compared to control cells. Original magnification, 200x. (**H**) Representative images from H&E-stained lungs from the same animal groups. The number of lung metastatic foci was significantly lower in the *MADD* knockout than in the control groups. Original magnification, 25x, 200x, and 400x, respectively.

To validate our findings in vivo, we generated an orthotopic mouse model of ATC by injecting 1 × 10^6^ of 8505 C cells (control, sgRNA-transfected, or *MADD* knockout) into the right thyroid glands of athymic nude mice (*n* = 6/group). Tumor growth was monitored, and the mice were sacrificed when weight loss exceeded 20% or at the end of the observation period. Remarkably, *MADD* knockout significantly delayed tumor onset and reduced tumor growth compared to control groups (*p* < 0.001, Fig. 5A-C). The Tumor volume in the *MADD* knockout group (32.5 ± 7.5 mm3) was significantly smaller than that in the vehicle control (434.0 ± 401.3 mm3), plasmid control (180.8 ± 56.53 mm3), and drug control (151.8 ± 44.43 mm3) groups (*p* < 0.005). Similarly, tumor weights were significantly lower in the *MADD* knockout group (49.5 ± 9.5 mg) than in the control group (327.0 ± 261.5 mg, 115.67 ± 55.70 mg, and 107.6 ± 29.62 mg, respectively; *p* < 0.005). The median survival of the mice bearing *MADD*-deleted tumors was significantly longer than that of the control mice (142.5 vs. 31–38 days, *p* < 0.001; Fig. 5F).

### *MADD* gene deletion prevents ATC metastases in athymic nude mice

Histological examination of the lungs revealed widespread micrometastases in control mice, which were largely absent or degenerated in *MADD* knockout mice (Fig. 5D). No significant differences in lung weights were observed between the groups (Fig. 5E), likely due to the small size of the metastatic lesions. Hematoxylin and eosin (H&E) staining of the primary thyroid tumors showed the replacement of normal thyroid tissue with pleomorphic tumor cells with hyperchromatic nuclei and prominent nucleoli (Fig. 5G). Occasional tumor giant cells and focal apoptotic bodies were also observed. Immunohistochemistry for Ki-67 revealed a dramatic reduction in the number of proliferating cells in the *MADD* knockout tumors compared to controls (Fig. 5G). In the lungs, H&E staining identified focal aggregates of atypical cells with pleomorphic nuclei, which was consistent with metastatic tumor cells, in the control groups (Fig. 5H). In contrast, the lungs of *MADD* knockout mice presented only scant, degenerated tumor cells.

Collectively, our results demonstrate that *MADD* plays a crucial role in promoting ATC growth and metastasis and that its deletion leads to cell cycle arrest, apoptosis, impaired migration in vitro, reduced tumor progression and improved survival in vivo. These findings highlight *MADD* as a potential therapeutic target in this aggressive malignancy.

## Discussion

This study demonstrated that *MADD* plays a crucial role in regulating ATC cell survival, proliferation, and metastasis. Using CRISPR-Cas9-mediated knockout of *MADD* in three ATC cell lines with distinct mutational backgrounds, we observed significant impairment of cancer cell functions in vitro and dramatically reduced tumor growth and metastasis in vivo. These findings highlight the promising potential of *MADD* as a novel therapeutic target for this highly aggressive malignancy.

Our approach of targeting exon 3, which is conserved across all identified *MADD* isoforms, enabled us to comprehensively evaluate the effects of *MADD* loss-of-function in ATC cells. This strategy mitigates the limitations of previous isoform-specific knockdown studies, which could not exclude the possibility of compensation by other isoforms. The consistent results across three different ATC cell lines with varying genetic backgrounds (BRAF, HRAS, and NRAS mutations) suggest that the dependence on *MADD* is a common feature of ATC, irrespective of the specific driver mutations.

In our study, we observed a significant increase in apoptosis in *MADD* knockout ATC cells. This finding aligns with *MADD*’s known role in regulating cell survival and apoptosis through its interaction with death receptors and various signaling pathways, including the TRAIL pathway as reported previously^10,34–36,40,43–47,52–55^. The absence of *MADD* in knockout cells may lead to increased sensitivity to apoptotic stimuli, as the inhibitory effects of *MADD* on proapoptotic signaling are lost. This mechanism could explain the increased apoptosis observed in *MADD* knockout ATC cells, as demonstrated by our Annexin V/PI staining assays. These results highlight the importance of *MADD* as a key regulator of ATC survival.

The transcriptomic changes uncovered through our RNA-seq analysis provide mechanistic insights into how *MADD* contributes to the progression of ATC. The alterations in genes associated with cell cycle regulation, apoptosis, and metastasis corroborate our functional assay results. Similarly, the changes in the expression of genes involved in cell cycle progression explain the G0/G1 arrest observed in *MADD* knockout cells.

The reduced migration and metastasis of *MADD* knockout ATC cells are particularly noteworthy given the highly invasive nature of ATC. These findings are consistent with previous studies indicating that *MADD* affects cell migration and metastasis through its involvement in various signaling pathways, such as the TNFα-induced activation of MAPK, Erk1/2, and Wnt-β-catenin signaling^9,33,39,56^. Our in vivo results, which showed a significant reduction in lung metastases in mice bearing *MADD* knockout tumors, provide strong evidence for the role of *MADD* in promoting ATC metastasis.

The profound impact of *MADD* deletion on tumor growth and survival in our orthotopic mouse model underscore the potential therapeutic significance of targeting *MADD* in ATC. The extended median survival (142.5 days vs. 31–38 days) and reduced tumor burden in mice bearing *MADD* knockout tumors suggest that *MADD* inhibition could potentially improve outcomes for ATC patients. These results are particularly promising given the current lack of effective therapies for ATC and the rapid development of resistance to existing treatments.

Our analysis of TCGA data^51^ revealed an association between high *MADD* expression and poor prognosis in thyroid cancer patients, further supporting the clinical relevance of our findings. While these data primarily pertained to differentiated thyroid cancers due to the rarity of ATC, they suggest that *MADD* may serve as a prognostic biomarker across different thyroid cancer subtypes.

Despite the compelling evidence presented in our study, there are some limitations to consider. The use of cell lines and a mouse model may not fully replicate the complexity of human ATC. Future studies using patient-derived xenografts or organoids could provide additional insights into the role of *MADD* in a more clinically relevant context. Furthermore, while our CRISPR-Cas9 approach effectively deleted all *MADD* isoforms, it does not allow us to dissect the specific contributions of individual isoforms to ATC progression.

## Conclusions

In conclusion, our study demonstrated that *MADD* is a critical regulator of ATC cell survival, proliferation, and metastasis. The use of CRISPR-Cas9-mediated knockout allowed us to comprehensively evaluate the effects of *MADD* loss-of-function in ATC cells, overcoming the limitations of previous isoform-specific knockdown studies. The integration of in vitro functional assays, in vivo mouse models, and RNA-seq analysis provides a comprehensive understanding of the impact of *MADD* on various aspects of ATC biology.

These findings highlight the potential of targeting *MADD* as a novel therapeutic strategy for ATC, which may help to overcome the limitations of current therapies and improve patient outcomes. Future studies should focus on developing specific *MADD* inhibitors and evaluating their efficacy and safety in preclinical and clinical settings. Additionally, investigating the potential synergistic effects of combining *MADD* inhibition with existing therapies could lead to more effective treatment strategies for this aggressive malignancy.

## Supplementary Information

Below is the link to the electronic supplementary material.

Supplementary Material 1

Supplementary Material 2

## Data Availability

The RNA-seq dataset generated and analysed during the current study is available in the NCBI GEO repository under accession ID GSE284824 (Secure token for review purposes: azwzswimzreztqb). Kindly contact Bellur S. Prabhakar, at email address bprabhak@uic.edu to request the data from this study.
