# Supplementary material for: CRISPR/Cas9-mediated deletion of MADD induces cell cycle arrest and apoptosis in anaplastic thyroid cancer cells
Source: Sci Rep. 2025 Nov 10;15:39264. doi: 10.1038/s41598-025-22907-1 (PMC12603207; doi:10.1038/s41598-025-22907-1)
Supplement: Supplementary file 1 — Supplementary Material 1 [file 41598_2025_22907_MOESM1_ESM.docx]

**
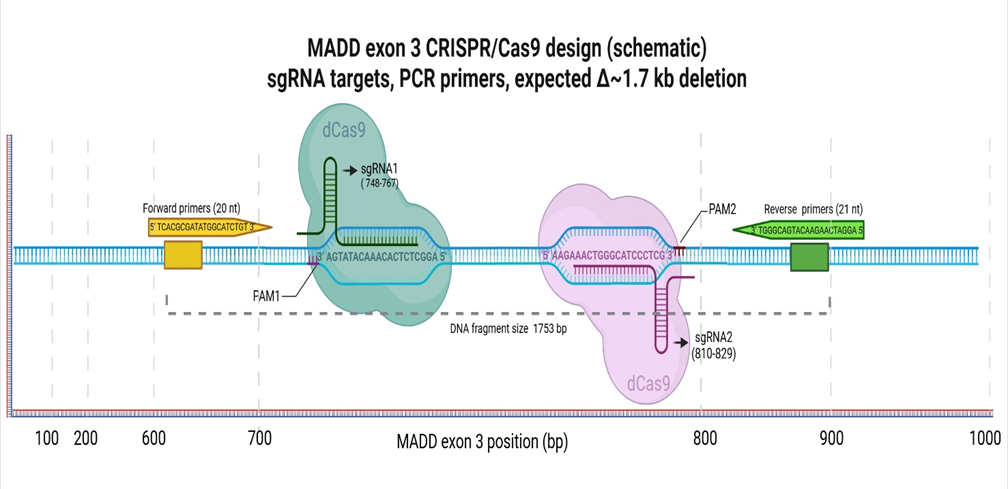
Supplementary Figure 1:**

**Supplementary Figure 1:** This figure illustrates the sequence of exon 3 of the MADD gene, highlighting the locations of sgRNA 1 (nucleotides 748-767) and sgRNA 2 (nucleotides 810-829), as well as the Cas9 double-strand break sites near the PAM sequences. Additionally, it indicates the binding sites for PCR primers in the surrounding region. The cleaving action of Cas9 at both sgRNA target sites leads to the excision of a DNA fragment of approximately 1.7 kb.

**Supplementary Figure 2A:**

**
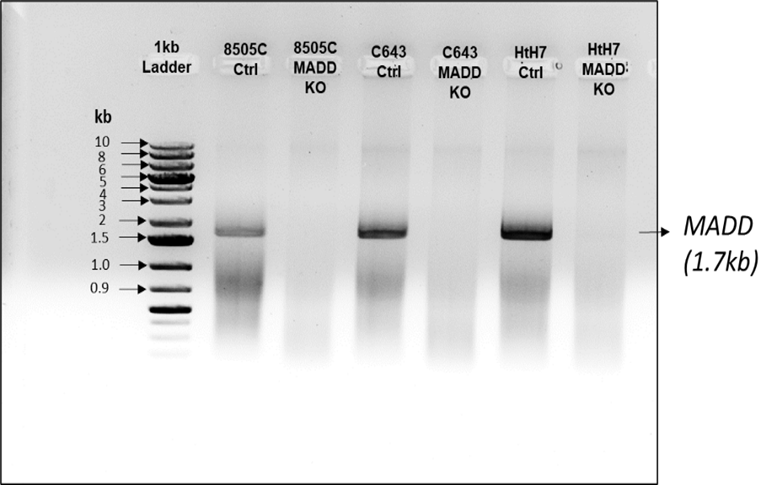
**

**Supplementary Figure 2B:**

**
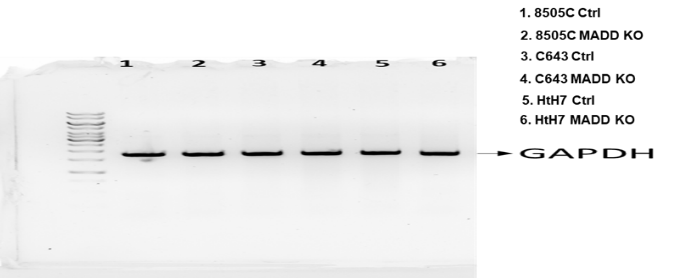
**

**Supplementary Figure 2C:**

**
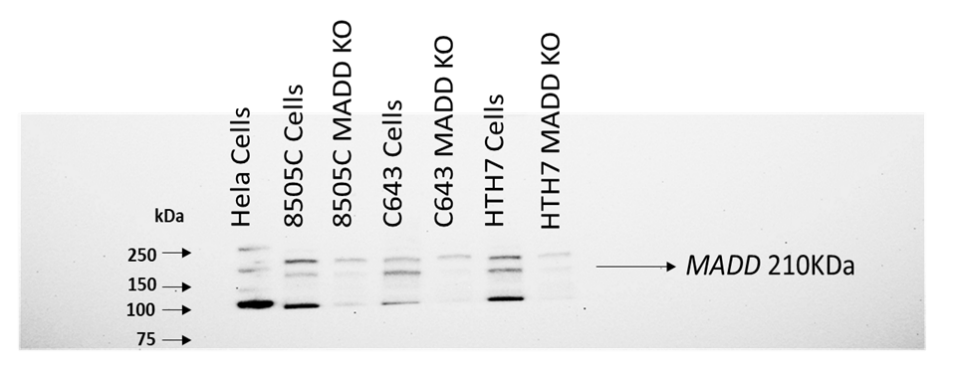
**

**Supplementary Figure 2D:**

**
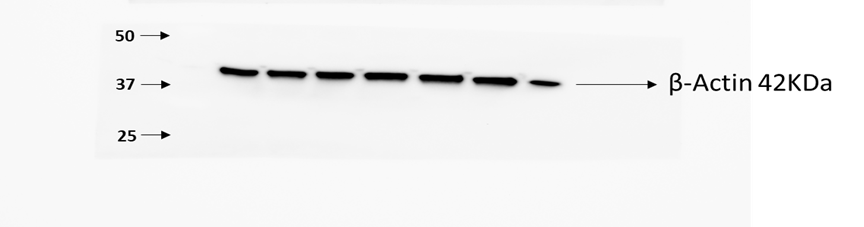
**

**Supplementary Figure 2:** Figure 2A) displays PCR results for MADD, confirming the successful knockout of *MADD* in thyroid cancer cells. Figure 2B) shows GAPDH as a control for the PCR results. Figure 2C) presents the validation of the *MADD* knockout using Western blot analysis across various thyroid cancer cell lines. Figure 2D) illustrates the corresponding β-actin results from the *MADD* knockout Western blot.
