# Supplementary material for: CRISPR/Cas9-mediated deletion of MADD induces cell cycle arrest and apoptosis in anaplastic thyroid cancer cells
Source: Sci Rep. 2025 Nov 10;15:39264. doi: 10.1038/s41598-025-22907-1 (PMC12603207; doi:10.1038/s41598-025-22907-1)
Supplement: Supplementary file 2 — Supplementary Material 2 [file 41598_2025_22907_MOESM2_ESM.pdf]

sgRNA Sequence

sgRNA1_5' AAGAAACTGGGCATCCCTCG 3'

sgRNA2_3' AGTATACAAACACTCTCGGA 5'

(TCCGAGAGTGTTTGTATACT)

Primer:

Forward – 5’ TCACGCGATATGGCATCTGT 3’

Reverse – 3’ TCCTAGTTCTTGTACTGCCCA 5’

(TGGGCAGTACAAGAACTAGGA)

https://www.ncbi.nlm.nih.gov/nuccore/NC_000011.10?report=fasta&from=47269188&to=47330031

Homo sapiens chromosome 11, GRCh38.p14 Primary Assembly

NCBI Reference Sequence: NC_000011.10

GGATCCGCCTCCAGGCCCAGAGGAGGGAGAGTCACAAATGACAGCCTTGGCCGTGAGAGGGGGCCTCTTC

TTACTAACCCCCACTGCGCCATATGGGTAAATGGAATGCTGAGGCTTAGGCGCCCCCTTCCCCTGCTGGG

ATCCGGGTAGGATCCGGTTGCCTTTCTGCGGTGGAGGAGGAAGTGTTGGATTTCAGCGCTAAGGCGTCGG

CGGTGCACCTCACGTGCATGTGTAGCATGCCTTGGTTTTTCCTTTGGCATCTGAAAAAGGCACAACCTGA

AAGACCTAGAACCCAGTGTCGGTCCCCAGGCCCTTTGGGACAGGAAGAGAAGAGCCGTGTGGCCGCGGGG

AGGATGTCCTGAGGCGGGGCTGTCCTCGCGGACTGACTGGACTCCATCTCCCAGCGGGCGCCGCGGCGCG

GCCACGCCCCCCCACTCCCCGCGCGCGCCCGGTGGAGGTAACCGCGGCCGCGCGCCCCCCTCTGCCCCCG

CTGCCCAGGATTGGTAGGCTCCACCGCTCGGCAGCCGGCTTCCCTGCTCGGACGCCGAGCACCGCCAAAG

CGCGTGAGTGGGGGGTGGGGACTGGGGGATGGGGAGCCCGTCAGGAAGTCTCAGGGGCCG
ATTATCTTGG

GAAGTGACTTTCAGAGGAATCCCCATGTCGTCGAAACGGGCGCTATGACCCCGTCGGGTGAGA
GTCTCTC

TGAGCAACCCAAACAGAAGGTACTGCTGTGGGTCCCCCACTTGGCACGCGACGGCGCTGGG
TCGGCCTGG

CGCGCCCTCCCGCGCCCACGGGCGCTAGGCTAGGTCTGTGCGCAGCCGCTGTCTCCCGAG
GTCGGCCCCG

CCCCGCGGCGCTCGGCTCGGTGCAGCCCCGGCCCCCCGGGCTGAATGGTACGGCCCCGG
CCCCGGCCTCC

CCCTCCGCACCCCGCGCGAGTGGTGCGTGCCCCCCAGTGCAGACGCGGAACTGGCGCCC
GCTCGGAGCGG

CGCCGCGCTGGGGAGCGACTGACGCCCCGCTGCCGGGGGACGTCGGGCTGGGCCTGGC
CAGTCCCCCAGA

GCTTGGGAGGTAGGAGAGTCTGGGCCGGTGGGGTCCTGGGCGAGCGTCTGGGGACTCCGAG
CGGGAGACG

GGCTCTGAGAGGACACCGTGGCTCTTGGAGGAAGGGGTCTCTCAGGAGGCCCTGGGGGAGT
GTGAAGGGG

CGTGGGGACGGGGGTCTGCCGAGGAGGGGCGGGGACGGGGATGGGGGCGCCCCGGGCA
GAGGAGGGGGCT

CAGGCTACTGGGCTTGGTTCCTGCCGAGCGGGCGGCTCGGGTTCAGGTTCGGGGCGGGGG
CGGGGGCGGG

GGAGAACTGTCCAGCAGGTGAGGGGGCGGGCAGAGCTGGCCAGCCCTGGGGCCGAACCC
GCACAGCGCTT

GGCGGGTGTGCTTGTGCGTGTGCGTGCTTGCGCCTTATTTTTGGCTAGAATTTGGAGAAACACGT
TTGGT

TCTCAAGAAATTTTTTTTTTAACTTGATATTTCAAAATTGACTTGCTGGAGAGAGAACACCATTTCATC
T

TTCTGCAATCGCTCTCCTCCCCATCCTCGTGTATCTACCTTCTTCCACCTTTCCCTGCCCTCCCC
TCTAC

CCCAACCCTGCCAGCTGCCAGCCTCCTTGCTCCAGTTCTCCCATACGCTGGACCAGTCTGTG
CTGATGAG

CTTGCAAGGTCACTGCCTGGGGAGGTAGAGGAGGGGAGGAGAGCCTCCTGTTGTGTTCACGTC
AGCAGAG

ATGTCATATCGCTCTCTGGTTCCTGTGGTTTTACTGTGAGATCCTGTCCAAAATGAGGGACGAATC
CAAA

GGGTTGGTTAGCATGGCTATTTCTGCTGAAAAGGCATAGATAACTTAAAAGTTTGTGAATCCTAAAT
CTA

CCTCTGGGTGGAGCTGATGGTGATGTCGGACTTCAGCAGATAGATATGCATTTTGAGCTGGATGC
TTTTG

GAATACAGGAAGATAGGAACCAGAGGCTCTACGTTAGTAAACATGGTTGAGGCAAGAGGCAGA
GTCGGAG

CCTCGTTGGTTGTGAAGACAAGAGTGTGAGTTGTGGGTGATGTTAGAGGATTTAGTCTTTAGAGGA
GTTA

AAGGTGTTAGTCTTTTTGGGGAGGGGAGAGAGGAGAGAATGAGAAGGAAAATCTTAGTTATCTTTG
CAAT

CTGAATAACTGAAGGCAGTGGCTGCTGATCAGATGTGAACTCTGGCTAAAAATTTATCCTACCAC
CCGTA

GCTAGAATAATAACAAGATCTCAGGGTCCTTGTGGCTCTGGAAGGAAATGATTGTGAGTGACTTGA
GTTT

CGTCAGATAGTTGTCAGTGGAGAGGGACTGAGATCCCACCAAGGCTAGAGAGGCCTGATGCGT
ACTCAGT

AGGAGGTGCTTCAGGCCTGATCTTTGAGATTTTGGTTGGAGAGGGCTCCGGGTTTTCAATGTAAA
CATAG

ATTGCAAGGTGTAGTCTAGAGCTCTGGTTCTATAAATTCTGTTTTACTTTAAGATACTTTGGCTCTCA
CT

CTAGTCATTTCTACATAATTCTTTTTTTTTTTTCGAATCCCATGACAGGTTAACTACATAATTCTTTATT

AAAACAAATTATGCTTTTTACTCATATCCCATTTCATTTCTCTATTTCTCTCCCCCTTGGATTCTGCTC
A

GGAGGAGCTGAGTGCGGCTCTGTTATAGTGCCCCACCTCCTGCAGTTTGCTCATGACTCTATTG
CAGTTG

GGAGAACCAAGGGACTATTTGGGTTCGTTAAGCAGTGGTGATACCTAGAAATGAATATATGGCGT
GCTCA

GAAACCTGGTGGTGTTCTAGAGATATCACTATCAAAAGCCAGAATTAGGACGAAGTACAAACTAC
AGACA

GATGAGGGAGAAGACAACCTAATTCTTAGCAGGTACTGTCAGTGGGAGAAGTGTCTCTAGGTTCG
AGGAG

AGTGAATTGTTATGGGCCTTGGCTGACTCTGGAGTAGCAGCTGCTAGCACTGTGCGGTCACCAA
GCAGGG

GAGGGGCTGTGGCTCTGCTATAAATAGTGCTGGGAGCCAGAGTCGCTATAGAGATAAAACTGAAT
CCTAT

TACCTGCAGGTCCTCTACTGGGAGCAAGGGAAAGGAGAAAGCAGTATTTTGAGGATTTGAGAGG
TATTGC

AAATAGGTCTTGTTTATTGTTAGGTAGGGGAGAAAAAACTTCTACTCATTACCTAAACCAAATAGGG
CCT

TAAGCATCCAGGGAGTGTGTATGTCTGTATATATGGGAGTGCCCGCAGCAGCAGGTGAGGGAGT
AGAGGG

TGTGTGTGTGTATACGATAATGGGTTTCTCTCCGTGTGCATTGAACAAGGTCTTAGGCCGCATGTA
GAGC

ACAGAGGTAGAATCCTGGAACACTAGAAATATGAGAAAAGAACTTCTACTCATAGAAGAAGTAGC
CCTAC

CAGAAATAGCTGTGCAGCGGGGTGAAACGTTTTATTTAGAGAAGTGTATAGACACTTAACACGAA
ATAAA

TTATAATTAAGACCATGAAAGCTTTTCCTAATTTTAGGACATAGTTTAGAAATTTCTCTGAGCACGTCT
C

CCTGTCCTTGCAGAAATCCTGGTATTTCTGCCTCTAATTCATAGCCTCTGTAATGTATAGTAGGACA
AGG

ACTCATCTGTGGGTATTGTATTACTGATTCTGAGCCTGAGATCAAAATAGAGGAGAGATTATGATTC
AGC

ACCAAGGGCAGAGCTAGTAGCAGTGGCCTCTGGGCTCCTTTCTTGCCTGAAGGAGGGAGAAAA
GTGGTGC

AGATTAGGCAATTGGGGCATGGACATTTTCATTTGAGATCACATGAGGTAGAATAGCCAGCTGTTA
TTGT

CTGAGCATGGTTAACTAGACAGTCTATGTGGTAGTTTTACATCAAGCTGAATGTAGATTGTTGAAAC
AGA

ATAATTAAGTTTCCATCTAGACCTAGAAGTGCAGGTTAGTGCTAGCTAGTTGAGGAGTCTGGTTTGA
TCC

TGATCCACCCTGGAGGGATCCTGAGAGTGACTTCAATTTTTGTTACAATGACTGAGTCCAGATTAG
ATGG

CCTAGGTCTGTTTTTCAGAAACAGAGCCCCAGGCGTTAAGCTGGAAATTAAGTCATTGGTAGGGA
TAGGT

AGGTGAACCATTATTTATCTACATTTTTTTTTTTTTCTTGAGACGGAGTCTCACTCTGTCACCCAGGC
TC

GAGTGCAGTGGTGCGATCTCAGCTCACCACAGCCTCCACCTCCTGGGTTCAAGCGATTCTCAT
GCCTCAG

ACTCCCGAGTAGCTGGGATTACAGATGTGTGCCACCACACCCGGCTAATTTTTTTGTATTTTTAGT
AGAG

ATGGGGTTTTGCCATGTTGGCCAGCTGGTCTCAAACTCCTGACCTCAGGTGATCCGCCTCGGC
CTCCCAG

AGTTCTGGGATTACAGGCGTGAGCCACCATGCCTGGCCTATCTATTTTTTAACATTCGATTCTGTG
AGCT

CATGAGCCTAGACACGAGGCTAGGCAAGCGTCAAGTTCTCCTATCATTAGTATGAAAGTGGAAGA
CCTTC

TGACCTCTGCCTCTGGCCGGGAAGAAGTTGAGAGGGATGAGTCCCTTAGGCACAGCAGTGGAT
CTTATCA

GTACTTTTTTTCCTATTAGACTTCGATTTTCAGAATTCCTCCTGGGAATGCTGACTCCTTGCTTGGTG
CC

CTGATGCTTCTCTGAGATAAACTGATGAATTGGAACCATGGTGCAAAAGAAGAAGTTCTGTCCTCG
GTTA

CTTGACTATCTAGTGATCGTAGGGGCCAGGTAACCAAGAAGAGATTGACTTTTGTCTTAATATCTG
GGAT

AGCCATAAAATCTGCAGTTGTTCCCTTCTCCGATTTCCATGTTGCTTTGCATAGCTCATCTTCTCCT
GTT

ATTTTACCCTGATGGGAGCATCGAAGCCAGTAGGGAAAAAACAAAATCAGGTGGATTGAAAGCA
AAACCC

TGCTCTTAACTAACATCTTCTTGATGTCGAGCAAATATACTCTTTATACCTCAGTTTTCTTATTTTGTAA

AAAGAAAAAGTACATTATAAGCTACTCCTCTATGTATACTTTTTTGGTAATTTATTAGTTAATTCACTTT

AAAAGATGTTAATCATCTTAAGGTGCCAGTGATAATTTTATTGTCAAAATCTCCCCTTCGTTTCAGAA
TA

GATGCCTTCATGACAGTGTAGACTTCACTTAGGTTACCAGCTCCATGCGGCAGGCAGCACCCAT
ATTTTC

CAAGGTGTTACAGTCAGGTTACTTTATCCAAAATAGCATCTTGAGCTTTATTTGTTGACTGTGGTTAA
AA

TTTGATAGCCCATTCCATCCCTTCAGGCTGCTGAATTTGTCTTTATGTTCTTCAGGCACCCGAGCA
GTGA

TAGCGTGGCCCAGACTCCTGAATTGCTACGGCGATACCCCTTGGAGGATCACACTGAGTTTCC
CCTGCCC

CCAGATGTAGTGTTCTTCTGCCAGCCCGAGGGCTGCCTGAGCGTGCGGCAGCGGCGCATGAG
CCTTCGGG

ATGATACCTCTTTTGTCTTCACCCTCACTGACAAGGACACTGGAGTCACGCGATATGGCATCTGT
GTTAA

CTTCTACCGCTCCTTCCAAAAGCGAATCTCTAAGGAGAAGGGGGAAGGTGGGGCAGGGTCCC
GTGGGAAG

GAAGGAACCCATGCCACCTGTGCCTCAGAAGAGGGTGGCACTGAGAGCTCAGAGAGTGGCTC
ATCCCTGC

AGCCTCTCAGTGCTGACTCTACCCCTGATGTGAACCAGTCTCCTCGGGGCAAACGCCGGGCC
AAGGCGGG

GAGCCGCTCCCGCAACAGTACTCTCACGTCCCTGTGCGTGCTCAGCCACTACCCTTTCTTCTC
CACCTTC

CGAGAGTGTTTGTATACTCTCAAGCGCCTGGTGGACTGCTGTAGTGAGCGCCTTCTGGGCAAGA
AACTGG

GCATCCCTCGAGGCGTACAAAGGTACAGTTTGCTGCTGATGCCTAATGGGGGAAGCATTTAGAG
ATTCCA

TGTAATTGGTCTTTGAGGGGCATAGGAAATTTCCTCTACAGCTCAAGGTCCTTCAGACCTATTAAG
CATA

TTTAGAGTATTTAGAGTTAATCTTTTTTCTTTTGGAGACAGTCTCGCTCTGTTGCCCAGGCTGGAGTA
CA

GTGGTGCGATCTCGGCTCACTGCAACCCCTGCCTCCTGGGTTCAAGTGATCCTTCTGCCTCAG
CCTCCCG

AGTAGCTGGGATTACAGGTGCACGCCACCATGACCAGCTAATTTTGGTATTTTTGTAGAGACAAG
GTTTC

GCCATGTTGGCCAGGCTGGTCTCAAACTCCTCACCTCAGGTGGTCCACCCACGTTGGCCTCC
CAAAGTGC

TGGGATTATAGGCGTGAGCATCATCCCCAGCCAAGAGTTAATCTTTACTAAGCAAAAGACTGCTG
CAGAG

TTAGACCTCTTTCCAAAATTGTGTTTGGAGTAATGCTGGAATTTGGTGTTTTAACTGTGGTTAGGACA
GT

AAGTCTTACCCAGTATATATCTTTACATTTGCTTTTGCCATTGTATATACTTTCCGGTCCTATCTGAGC
T

CAGAGACTGGGCTTTCTTAATGATGCTCCGTTTCCCATTGTCATCATTAGAGCCTCTGTGGTGATA
CTGA

GTTGCAGTAGGGTATCAGCTTCTGATGCTAATGTGTCTGATTCCTGCTGTCTGCTTCTCAGGGACA
CCAT

GTGGCGGATCTTTACTGGATCGCTGCTGGTAGAGGAGAAGTCAAGTGCCCTTCTGCATGACCTT
CGAGAG

ATTGAGGCCTGGATCTATCGATTGCTGCGCTCCCCAGTACCCGTCTCTGGGCAGAAGCGAGTA
GACATCG

AGGTCCTACCCCAAGAGCTCCAGCCAGCTCTGACCTTTGCTCTTCCAGACCCATCTCGATTCAC
CCTAGT

GGATTTCCCACTGCACCTTCCCTTGGAACTTCTAGGTGTGGACGCCTGTCTCCAGGTGCTAACC
TGCATT

CTGTTAGAGCACAAGGTGAGAGGCAAGCTTCCTAGACCTTTCTAGGGGAGAAACTCTTAAATATG
CCAGT

GGCCAGACCACGAGCTCTTAGAGGACAGGGACTACATATTTTTCACCTTTTTGTCTTCAGGAGGT
AACAT

GATGCCTGGTAAACAGCAGGTGTTCCGTAGATGTGGGTTGAATTAAGATACTGGTATCATGTGGGA
TGTA

TTTAGAGGGTTTACCATTCATTAGGGCTTATCCTTAATTGTACTGACCTAATTCTTTGGAAATATCAGT
A

GTTCTGTTTCAGTGAGTGGTCCTGGGATGGGCAGTACAAGAACTAGGAAGGTAAGGGAGTGATTT
GCATA

TCTCAGGTGACTGAGGATCACCATTGTGCCTGGATTGGGAGGTGGCAGGCATGGTGGGGCAGG
GGGCAGT

GGGAGAGACAAAGCTGGAGCTGTAGGCTCGATGAAGTGGAAACCAAGTTGTAATGTTGGAAGCT
GTTTTC

TTGTAAACCATATTTTATGTTTTGGAGTGATTCTTACTGGATGGCTCATGACAGGTGGTGCTACAGTC
CC

GAGACTACAATGCACTCTCCATGTCTGTGATGGCATTCGTGGCAATGATCTACCCACTGGAGTATA
TGTT

TCCTGTCATCCCGCTGCTACCCACCTGCATGGCATCAGCAGAGCAGGTGAGTCTCAAGGCGAC
TTCCGGC

TTTCTCACCTCTGTCTTCCTGAGGGAGGAGGCTTAATGCTCAAAGAAGTTGTAAGTGAGTGCTGG
TCCTC

AATCCTTTGTCATAACTTATTTGTCTACAAAATCTGACTGATTTGAACTGATCTGGTGGCAAGGCTTA
TG

CTATTTTAGTTTTCATTTATCACAACAAATGTTAATGACTGTAGGCCACTGGCTACACTGCAGCAGG
TGT

TACGTGTGTTACGTAATACAGTAGTTCCTCCTTATCTGTGGAGGACACATTCCAAGACCCACCAGT
GGAT

GCCTCAAACCACAGATAGTTCCAAACTCTATAGATACTTTGTTTTTTTCTATACATATATACCGGTGAT
A

CCGTTTAATTTATGAATTAGACACAGTAAGAGAGTAACAGTTTTCTCTTTGGGCATATATTTGGTGTTT
T

TTGGAGACAGAGTCTCCCTCTATCCCCCAGGCTGGAGTACAGTGGCACAATCTCGGCCCACTG
GAGCCTC

TGCCTTCCAGGTTCAAGTGATTCTCCTGCTTCAGCCTCCCTAGTAGCTGGGATTACAGGCACTG
CCACCA

CCACACACCAAATAATTTTTTGTATTTTTAGTAGAGGCGGGGTTTCACCATGTTGGCTGGGCTGGT
CTTG

AACTCTTGACCTTAGGTGATCCGCCTGCCTCAGCCTCCCAAAGTGCTGGGATTACAGGCATGAG
CCACCG

TGCCCAGCCTCTTTCTACATATATTTGGCTTCTCTTTGGCATATCCCAATTGCCAGCATCAGTACC
CTTG

TGCTTTGGGGCCATTATTAAGTAAAATAAGGGTTACTTGAACACAAACGCTGAGATTGTGTGACAG
TTGA

TCTGATAACCGAGATGGCTACAGGCTGGACACAGGGGTGATTCATGTCTGGGTGGGACAGAGC
GGGACAG

CATGAGATTTCATCATGCTTCTCAGAACTATTGTCCTGCACTTCAGATATGATCAAGCTGCAGTTTA
AAA

TTTATGAATTGTTTATTTCTGGAATTTTCCTTTTAATATTTTCAGACTACAGTTTGCCTCAGTTAACTGG

AATCACAGAAAGTGAAACTGTGGATAAGAAAGACTACTGTATACAAAAAAATGTACCATGTTACCTT
TCA

AAAAATCTGAAAAATTCTGAATTTTCAAATACATCTGGCCTCAAGAGTTTTGGAAGAGGGATTGTAT
CTG

CATTTCCTTATCTAGAAGAATCCAGACTTGATAAGTGCTCATGATAGGTTTTGTCTTTGTTTCTCACC
TT

CTTTATCATTTCCACAGCTGCTGTTGGCTCCAACCCCGTACATCATTGGGGTTCCTGCCAGCTTC
TTCCT

CTACAAACTGGACTTCAAAATGCCTGATGATGTATGGCTAGTGGATCTGGACAGCAATAGGGTGA
GGTTC

TTGGCTGAGGCATTGTAGAGTCTAGAGGAGGATTGCATTCTAGACCCAGTCCTGAGATATCTGTTT
CTAG

ATTCCTTTCAGGAACGTCCTAAGTTGTTGGATAATTTCTATTGCGAGACTTACTGAAACAAGTTTTTTA

AATGCTCCTTTTACCTTATTAGCCAAATAAACCCTTGGTTAGGTCTCAGAGAATTACAGGATTTATTTTT

GCCAGAATTAAGTGACCCCCCACCATATCCTCCAAGAGCAGTCAGACAGTTGTGATCTGAGGGCCATTAC

ATGTGGTATAGATCATCTCTTAATGGTTTCCCTGGGGTATAGTTTCCAGTAAGAAACTGTCTTTCAGTGC

AGGCTTAATTAATTATATGACCATTCTGCAATGTGAATACATTCAGACTTTGGTGGGAAGCCCTTATGTG

TGCTCATTGTAAAACTTAAAAAAAATAAAAATAAGCGTAAATAAAATAAGTGAAAAGAAAAACCATAATC

CTTTTATCTAGAGATAATAACCAATAACATTAAGTTATATTTCATTTCAGATGCATATGTATGTCATATA

TGTGTGTACATATATCTTACTGTGCAGTTATATAATGTATATTCGTTTATATAATAATAATGTATATACG

TCCTCTTATTTTTATTTATGTAAGTGAAATTCCTGAGCAATAAACTCTAAGGTCACGTCTTTTATCATTT

CTCTGGTGCAGGTGATTGCCCCCACCAATGCAGAAGTGCTGCCTATCCTGCCAGAACCAGAATCACTAGA

GCTGAAAAAGCATTTAAAGCAGGTAGGTGAAGAACGAAGGAAAGAAAGGGGAGTATTAGATGCTGTGGGA

TTCCTATAAGAAGACAGCTATTCTCAGAGCCAATTTCCTATCAAACTTCAAGTGGAGTAGTTTTCTTCAC

CCTGGATGGGCTTAAGAAAACGCGTATCCCATTTCTGGGTGGCAGAGTGAATTTCTGTTTTTGATTCCAA

TTCATTGTATGCTTTACCATCTTCTAATGCTTATGATGCTGTATAAAATAGCCCTTCACTCTCTGTTTCT

TGTATGTGTGCCACTCTCTATGGCTTCAGAACATTTTCTCAGTCTTTTTTTTTTTTTTTTTTTGAGATGT

GGAGTCTTGCTCTGTCCCCTAGGCTGGAGTGCAGTGGCGCGATCTCGGCTCACTGCAAGCTCTGCCTTCT

GAGTTCACGCCATTCTTCTGCCTCTCCAGGAGCTGGGACTATAGGCGCACGCCGCCACACCCGGCTAATT

TTTTTGTATTTTTGGTAGAGACGGGGTTTTGCCATGTTAGCCAGGATGGCCTCTATCTCCTGACCT
CATG

ATCCGCCTGCCTCAGCCTCCCGAAGTGCTGGGATTACAGGCGTGAGCCACTGCGCCCGGCC
GATTTTCTC

AGTCTTGTTAGGTGGAAGTTAGTAGTGTGGACCGTCTGGCTGCTTTTATATTATCTTCTAGAGTTGAT
CC

TGAGGGTTCCTTTCTTCAAAAATTCCTTTATTTGGCTGGGCACGGTGCCTCATGCCTGTAATCCCA
GCAC

TTTGGGAGGCCGAGGCAGGTGAATCACGAGGTCAGGAGTTGAAGACCAGCCTGGCCATTATGT
TGAAACC

CTGTCTCTACTAAAAATACAAAAATTAGCCGGGCATGGTGGTGGACGCCCGTAGTCCAGCTACT
CAGGAG

GCTGAGGTAGAAGAATCACTTGAACCCAGGAGGCAGAGGTTGCAGTGAGCCAAGATTGCGCC
ATTGCACT

CCTGCCTGGGCCACAGAGTGAGACTCAGTCTCAAACAAACAAAAAAAATTCCTTTCTTTGAGTAAT
TCCTC

ACATCCATTTTTTATTGATTGATTTTCTTCTTCCTCTGCATGCTGAACACAGTACCTGAAGGTAACAC
AT

GACAAAGCTTCATTCCCTTCCTCTGCCTTATGTGTTGTACAGGAACACCAGTAGATAACACTGTGT
TTAT

TTTTAGGAATCCCTGCTCTCATTTGAAGGGCCTTTAGAAATCTTTTTCTGTGTTGTCTGAAGTACTTA
GT

AGTATCTCTAGATGCTATGTTTGGGTTTAAATGGGTCATTGTGTTTCATCCCTCTCACCTCAGTTGCT
TC

ATCACTGCTAATATAATATGATACCTTGTTACTGCAGAGTCTAGTGGGAATTTTAAGTTCAGAAAGTT
CT

CTAGCTGCCTTCACTTGCTGTGTTTTCAGTCTTGATGATTGCTTTAGTATGACTAATGAGATGGAGA
GGT

TAATAAATGGAATTATATTTAATTAAATTAATTAATTAATTTATTTATTTATTTTGAGATGGAGTCCCAC

TCTGTCACCCAGGCTGGAGTGCAGTGGCACAATCTTGGCTCACTGCATTCTCTGCCTCCCAAGT
TCAAGC

GAATCTCTTGCCTCAGCCTCCTGAGTAGCTGGGATTCCAGGCACACACCACTGTGCCCAGCTA
ATTTGTT

TGTATTTTTAGTAGAGCTGGGGTTTCATCATGTTGGCCAGGCTGGTCTCAAACTCCTAACTTCAGG
TGAT

CCACTTGCCTTGGCCTCCCAAAGTATTGGGATTACAGGCATGAGCCACCGTGCCCAGCCAGAA
TTATATT

TTAAATGTTTGTAGGAGCTAAGATCCTTGGACTCATTGATCCTGTGTTCTAACTCCTGCTATTCAAAG
TG

TGGTTTGTGAACCAGCAACATTAGCATCACTCGGGCTTGTTAAAGTGCAGGATCACTCCAGGCAT
ATTGA

ATCGGGATCTGCATTTTAACAGCCTCCCCTGGTGATTCTTCTCCTCACTCACATTTGAGAAGCACT
GCTG

CTCTCGACCAGTGGTCTTCACGCTGCCCCATTGAGCCTCTGAGGGGCCGATTTAGCTTGGGAG
CCCTGAG

CATTTGAGGCTCTGCTTCCTGTGTCCTTCAGAGCAGCTCTGTTTTCAAATTTCCATTCTATATAATTT
CA

CATAGATTCCAATTTTAAAAAACATTTAATGACTATAATAATTTTGCAAACGACTGTCGTGGACTTCA
GA

TAAGATCAAGTTGCCTTGAGCTAGGTTGTCTTAAGATGGAGAGCTACATATTTCAAACTGTTTTCTAA
AG

GCTAGAATATTGGGATTTTTATTTCAAAGATGAAAGGTCTGAAATGGGTTGGCCGTGATTTGCAAAT
TAG

CACCGTTCTTTGTTGCAGAACTTTTTGACCAGAGAATACGAGGTAGCAGACCTTAGGAAGGCCTT
TTCCT

TGGAGTTAAGATTGGCTAGCTGCCCAGGGACGTTCCTTGTGTAAGGAATTTTCTTGTTGTTCCACA
GGCC

TTGGCCAGCATGAGTCTCAACACCCAGCCCATCCTCAATCTGGAGAAATTTCATGAGGGCCAG
GAGATCC

CCCTTCTCTTGGGAAGGCCTTCTAATGACCTGCAGTCCACACCGTCCACTGAATTCAACCCACT
CATCTA

TGGCAATGATGTGGATTCTGTGGATGTTGCAACCAGGTAAGACCAAGCCGACTGTATAATCACAA GTTCT

TAAATTAATTTCTTTGCTCTCTAAGGGACCTTGGGGCAGACTATTTCTCTGAACCAGGGTTCCTTTT TTT

TTTGAGATAGATTCTCACTCTTGTTGGAGTGCAATGGCATAATCTCAGCTCACTGCAACTTCCACC TCCC

GGGTTCAAGTGATTCTCCTGCCTCAGCTTCCTGAGTGGCTGGGATTACAGGCGCCTGCCACCA CGCCTGG

CTAATTTTTACATTTTTAGTAGAGACGGGGTTTCACCATGTTGGCCAGGCTGGTCTGGAACTCCTG ACCT

CAGTTGATCCACCCGCCTTGTCCTCCCAAAGTGCTGGGATTACAGCCGTGAGCCACCGCACC CAGCCCAG

GGTTTCTTTATGTATAAAATATATACTATATTTGGTTGTTATTTTCCACCTTAGTAAAATCAAAGTGAAG

AAATAGTAAACCACGTGCTCAGGATGAAGAGGGGGCTGATGATTCTTTAAGATATCTCTCCCCTTG ATGT

TGGAAGCCTGTTAAGTGATGGCTTTGGGAGGTGTTCTAAGACTTTGGATCATGGGAATCCTTACC CTATG

GGTCTCAGATTATCTCATCATCTGCTTCCCCAGGGTTGCCATGGTACGGTTCTTCAATTCCGCCA ACGTG

CTGCAGGGATTTCAGATGCACACGCGTACCCTGCGCCTCTTTCCTCGGCCTGTGGTAGCTTTTC AAGCTG

GCTCCTTTCTAGCCTCACGTCCCCGGCAGACTCCTTTTGCCGAGAAATTGGCCAGGACTCAGG CTGTGGA

GTACTTTGGGGAATGGATCCTTAACCCCACCAACTATGCCTTTCAGCGAATTCACAACAGTGAGT CTACC

TGCCCTCTGCTCCGCTCTGCCTTGTGCCTCTGTCCTCCTGATGGACTTTGATTTTTCCTTTGCTAA TGTT

TGACCTGTGCCTTCTATCCTGGCTCTGCTTTAGACTTTTTCCTGCCTTTCTTTCAGGCGTTGCTTGC CTT

TTTTTCCTTGCTGCTGACTTTCTGTTCTTTTCCCTCATGGGGTAGATATGTTTGATCCAGCCCTGATT GG

TGACAAGCCAAAGTGGTATGCTCATCAGCTGCAGCCTATCCACTATCGCGTCTATGACAGCAATT
CCCAG

CTGGCTGAGGCCCTGAGTGTACCACCAGAGCGGGACTCTGACTCCGAACCTACTGATGATAGG
TGAGCAT

CCTTAGGGCAGCAAAAAGGTTTTAAAGGTGAGGAGGCTCTCACTAGCCCTTCTTTAGCACAGAG
AAGGCA

AAGTCTCATAGGCTTCCCATATCAGTGTTTTTAATTTTATTTTATTTTATTTATTTATTTTTTTGAGACG

GAGTCTCGCTCTGTCACCCAGGCTGGAGTGCAGTGACGCAATCTCGGCTCACTGCCAGCTCC
GCCTCCCG

GCTTCATGCCATTCTCCTGCCTCAGCCTCCCGAGTAGCTGGGACTACAGGCGCCCGCCACCA
CGCCCGGC

TAATTTTTTGTATTTTTAGTAGAGATGGGGTTTCACCGTGTTAGCCAGGATGATCTCGATCTCCTGAC
CT

TGTGATCTGCCCGCCTCGGCCTCCCAAAGTGCTGAGAATACAGGCGTGAGCCACCGCGCCC
GGCCTTATT

TATTTATTTTTGTATGTATGTGACAGAGTCTTGCTGCTCTGTCACGCGGTCTGGAGTGCAGTGGTGC
CGT

CTCAGCTCACTGCAAGTTCTCCCTCCCAGGTTCAAGTGATTCTCCTGCCTCCGCCTCCCAAGTA
GCTGGG

ACTACAGGTGCTGCCACCTTGCCTGGCTAATTTTTGTATATATATATTTTTTGAGATGGAGTCTTGCT
CT

GTCGCCCAGGCTAGAGCGCAGTGGCGTGATCTGGGCTCACTGCAACCTCCGCCTCTCGGGTT
CAAGCGAT

TCTCTTGCCCCAGCCACCCGAGTAGCTGGGATTACAGGCACATGCCATCACGCCCAGCTAATT
TTTTGTG

TTTTTAGTAGAGACAAGGTTTCACCATGTTGGCCAGGCTGGTTTCAAACTCATGCCTTCAAGCAAT
TCGC

CTGCCTCGGCCTCCCAAAGTGCTAGGATTACAGGCGCGAGCCACTGCACCCGGCTACTTTTTG
TATTTTT

AGTAGAGATGGTGTTTCACCATGTTTGCCAGGCTGGTCTCAAACTCCAGACCTCAGGTGATCCA
CCCGCC

TTAGCGTCCCAAAGTGCTTGGATTACAGGCATGGGCCACTGTCCCGGCCCCCATATCAGTCTTT
ATGCTT

TGGTTGACTGTTTTTCTTGGCTTCTTTATTTGATTTGTTCCCTGGCCTTGAGTTCGGAGTGCTCCCC
CTT

TTATTTTTCCTGGTGCTGATTGTAGGTGTTCTCCCTGCCCCTTTCTGTTTTGTTTGTTTTTTATGCACTT

CACTCTGCAGTGGCAGTGATAGTATGGATTATGACGATTCAAGCTCTTCTTACTCCTCCCTTGGTG
ACTT

TGTCAGTGAAATGATGAAATGTGACATTAATGGTGATACTCCCAGTAAGTGTGCTTGGGGAGATTG
GCCA

GCCCTGGGCAGGGGTTGGGGGCCCAAGGATGGAGTGGTCTGTACCTGCCTCCCTTGGATTTT
GGCAGATG

TGGACCCTCTGACACATGCAGCACTGGGGGATGCCAGCGAGGTGGAGATTGACGAGCTGCAG
AATCAGAA

GGAAGCAGAAGAGCCTGGCCCAGACAGTGAGAACTCTCAGGAAAACCCCCCACTGCGCTCC
AGCTCTAGC

ACCACAGCCAGCAGCAGCCCCAGCACTGTCATCCACGGAGCCAACTCTGTAAGTGAGGAGC
GGTGGATGA

GTGAGAACCATGGCCTGGGATGTGGGCCTCATCTATTAGGAAAGCCAGGCTGTAGCTATTCATTT
GCTGC

TTCTCACACAATTTTCTCATCTTCCTCTTCATGGGCCCTAGAGCTTAGAGCCTAAGAGACCTACTTT
TGA

CCCCAAAAGGAGGTCATCAGGCTTTGTTCAGGGTACAGCTATGATCCCCCTTATATAGTTTGTGG
GGACA

GAGAACGGGTGCTACAGAGATCATTTCCCACACATTCCTTAGGCTAGAAATCTGACAGGCCTGG
TCCAGC

CCTGCCAAACTGGGATTACAGGAAGGTACCATTGGGCACCAGGTAACCACTTTTAATTTCATGC
GGCCTT

TAGGAACCTGCTGACTCTACGGAGATGGATGATAAGGCAGCAGTAGGCGTCTCCAAGCCCCTC
CCTTCCG

TGCCTCCCAGCATTGGCAAATCGAACGTGGACAGACGTCAGGCAGAAATTGGAGAGGGGTCA
GTGCGCCG

GCGAATCTATGACAATCCATACTTCGAGCCCCAATATGGCTTTCCCCCTGAGGAAGATGAGGAT
GAGCAG

GGGGAAAGTTACACTCCCCGATTCAGCCAACATGTCAGTGGCAATCGGTGAGAGCCTGGGCAT
CCCTTCT

AGATGGGTGACTGAAGGACCTCACCTCAGTGGACCCTGGGCAAGGGTTAATCAGAAAGTCTGA
GAAGTCT

GAATGCTCTCGTGGTCCTGCCATCCCCAGAGGATGGTGTTCTGATCCAGGGGCTTAGGAATTAC
GGGAAG

GGAGTGGCAACTGTAGTATAGCATAATTATGGCCCATACTCCCAAGATCAGGTACCCCTGCCTG
GGGATC

ATAGGTGCCTCTGTGCATTCAAGGGCTCAAAAGCTGCTGCGGCCCAACAGCTTGAGACTGGCA
AGTGACT

CAGATGCAGAGTCAGACTCTCGGGCAAGCTCTCCCAACTCCACCGTCTCCAACACCAGCACC
GAGGGCTT

CGGGGGCATCATGTCTTTTGCCAGTAAGTGCCTTCAGCTGTCTCTCTCACTCCTGTGTTCCATTTT
CTCT

ACAGCAGAGCCTGACTATGGCAAATGTTGCTTAGAACTTCACATGTAGGGCTAGGTTGGCATTAG
CAAGC

CCTAATCCAGTCATTTGGGTGATACTGAATAAGGCGTTTTCAAGTGAGGTCAGTAAAGGACAGGG
ACAGA

TTAGTCATTATGCCTGGACATTTCAGTCTATTTCAGGTAAGACAGCTGGATGGGTAACTGGAAAAC
AGGC

AGTAAATTCAAAATCACACACTTGTACTTATATTTTGTGGAAATACTCTGGTCAAATTTGATTTGAGTC
C

ATATCTGGTATCTTCTTTGCCTCATAACGGACTCTTCCATGAATGTGCTTTGAAATCGATATGAGATT
GA

ATTTTGTGCTTAGATAGTATTCCCAGCAGTGCAGGGTCGTATCAGCTAAAGATTGAAGACAGACCA
TGGA

CTAGAGAGAGCGAACGTAGAGGATCCCTGTGATTGTGAGTTTCTTCAGCAGCCAGGGAGGGAC
AGTGCCA

GCTTCAGACCAGAAGCCTGTTAACTGCCAGCTTTCATGCTGTGTAGCAGTGGATGAGAGACTTG GATATT

ATCATGTGAGGTGGACAGAGAAAGCTGTTACTGGGTTTTGGCTGATCCTGGCAGTGGCTCCATTG TTTGC

CTGGAGTTAGACTTGATCCTTTCTAAGACAGATCAGAGATGATCTGATCCACCAAACTGGGATTGT GTTT

GAAAAGGAGGGAGGTGCAGATGCTGATAGTCATTAGTCTACTGCTTTGATTACTTACTCTGCCAAG TCAT

CGCTCTTGCACCCTCCCCTTGATAGGCAGCCTCTATCGGAACCACAGTACCAGCTTCAGTCTTT CAAACC

TCACACTGCCCACCAAAGGTGCCCGAGAGAAGGCCACGCCCTTCCCCAGTCTGAAAGGTAAC TACAGCCT

TCCTTTTGCCAAGCCAGGTTTCTCCGGGAGATGTTTCGGGCTGTGGGTTCATGTCAGGAGCCCT GCATCT

GCTGAATCCTTTGACCTGGTTGTCGTCCCTCATGTTGCTCCTCATGGCTTTCACCGCACATCCTG GGCTC

TGATGGTTGTCAGATGGAAACCATCTTTGTAGCTAGAGACGGCCCTGTTATGTCATTTAGGACAAT GGCA

ACTGTGGGATTACTGCTGCTTCGCGGGAGGACCCTGTCAGGGTATATGATTAACATAGGGATCAT CAGCT

GCCCCCAGTCGTCTGCTGGGTTGCCACTGCATGAACTTAGGGCAAATTCCGTGCCAAGGAGCT GGCCCAC

CAGAGAATGGTTATGGAGAGCCTGGCAGCGTTCATAGAGCACAGCCTCTCACCTCTCACGCAC TCATAAG

CCTGTCAACCCAGGGCTTAATGAGTGCGCCAGGTAGTATTGGTTAGTTTCTAGGGGCAGTTAAAA CCTTA

TGTGAAAGCCAGGCGCGGTGGCTCACGCCTGTAATCCCAGCACTTTGGGAGGCCGAGGCGG GTGGATCAC

GAGGTCAGGAGTTCGAGACCAGACTGACCAACATGGCGAAACGCCGTCTCTGCTAAAAATACA AAAGTTA

GCCATCTGGTGTCACGCACCTGTAATCCCAGCTACTTGGGAGGCTGAGGCAGGAGAATCGCTT
GAATCCA

GGAGGCGGAGGTTGCAGTGAGCCGAGGTCGCGCCACTGCACTCCAGCCTGGGTGACAGAG
CAAGACTCCG

TCTTAAAAAAACAAAAAACAAAAAACGAAACCTTATTTGAATTGGTTTGTGCTATCTGACAGTTTCA
GGG

GCTTTTCTGAATTTGATTTTTTATTTTTTTTATTTTCTGAGACAGTGTCACTCCGTCACCCAGGCTGGA
T

GGAGTGCAAGGTGGTGATCTTGGCTCACTGTAACTGCCGCCATCCAGGCTCAAGTGATTCACAT
GCTTCA

GCCTCCCGTGTAGTTGGAATTATAGGCACATACCACCTCGCCTGGCTAATTGTTGTATTTTTAGTA
GAGA

CGGGGTTTCACCATGTTGGCCAGGCTGGTCTTGAACTCCTGACCTTGAGTGATCTGCCCACCTT
GGCCTC

CCAAAGTGCCAGGATTACAGGTGTGAGCCACTGCACCTGGCCTGATTTTTTTGTTTTCTCTGGAT
GGTAT

GACGAAATATTTAACTGTGGTAGGAAGTGCAGTGAGAAACATGTATTTTTTTTTTTTTTTTTTTTTTT

GAGACAAGAGTCTCGCTCTGTTGCCCAGGCTGGAGTGCAGTGGTGCAATCTTGCCTCACTGCA
AGCTCCG

CCTCCCGGGTTCACACCATTCTGCCTCAGCCTCCTGAGCAGCCGGGACTATAGGAGCCTGCC
ACCACGCC

TGCCTAATTTTTTGTATTTTTAGTAGAGACAAGGTTTCACCGTGTTAGCCAGGATGGTCTCGATCTC
CTG

ACCTTGTGATCTACCCGCCTTGGCCTCCCAAAGGGTTGGGATTACAGGCGTGAGCCACCGCA
CCTGGCCA

ACCTCATGTATTCTTTAACTGACCTCTCATTCCTAGTGGTAAAAAATGTCAGGTTACTTGGGTGCAG
TAG

CTCACACCTGTCATCCTAGCTACTTGTTAGGCAGAGGCAGGAGGTTCATTTGAGCCCAGGAGTTT
GAGGT

ACAGTGAGCTATAATTAGGCCAGTGCACTCCAGCCTGGACGACAGAGTGAAACCCTGTCTCTAA
AAGAAG

CCAACAGAAACAAACAAAGTTGTCAGGAGTCTAAGACATGTTCTTAGGTGAGGATGGGAACATTG
GATAA

ATGGGCAAAAGGACATCAGAGCTGGCAGTGAAAGGAGCGAACTAGCACTGCCAGGGAATCCA
CATGTGGC

CGGGTAGGCTAAGGATGGAAGGGTGTCAGTGTCACTGGCCTTCATTTGGTACATTTGAAGAGAA
GAGCGA

TTGAAACTCTTTCATTGCATGCTGATGCTTTGTCGCATTTGCTGCACTTACCCTCTCACCCATCTCC
CAT

CCTCATACCCTGACCCCACTCCCCACAACAGCATCTGCAAGATGGGTTGCTTCCCTGCACATA
CCCTCAG

GCCCCTGGCCCTCTGTGCTGGATGTTTTCTGAAAACCAGCGGTCCCTGTTTCATGCATGTGTGTG
TTCAG

TACCTGAAGGCTCCCTGTGTCATTTACCCGCCCCTGGCTTGAGAGGCCAGTTTTAAGGGTGGTC
TTTTCT

TAGAGCCTTGGTTGTCTCCTGGTCACTTGGCTTCCAGCTCTGAGGTGCCCAAGAACCAAGGTTA
TGTGCA

TTCCTCAAGCTTTGGGAGAATGCTCAGATTATCTGGCTTACTGCTCCTCGGAGGGGTAGAGGGG
TTGCGG

TATTGTGGTACACCTACTAATTGTGTATTTTGTGTCCCAGTATTTGGGCTAAATACTCTAATGGAGATT
G

TTACTGAAGCCGGCCCCGGGAGTGGTGAAGGTGGGTCTTGCCTGTGAGCTGTGCATGATCTTTT
TTTTTT

CTCTAGCATCTTCTGTGCCTGCCCGAGGGCCATCCTTCTCATGGAGCAAGCAGCGTCACATGA
GTGACCT

GCTTGCCCCGTCCCCCGTGACGCCCATGCTTGCCCATGGGGCTTGCTGCTGGTGCATGTGGA
GTGGCCTG

AGTCTCCATCCCCACCTCCTCCACATTGCCATGGCTGGTTTCTACCTACGTATGATGCCTTGGTG
CTACC

CTCACCCAGCCCTTCTGAGGAACGGGCATGGAACATGGCTACTTAGGGCTGTGCTGGCATGAG
CTCCTGT

ACTACAATAGTGATGTCTCTCATTCCTGCCTTCCCTGCCACAGGAAACAGGAGGGCGTTAGTGG
ATCAGA

AGTCATCTGTCATTAAACACAGCCCAACAGTGAAAAGAGAACCTCCATCACCCCAGGGTCGATC
CAGCAA

TTCTAGGTAATAAATGGTAGAGCTGTTTTAAAAGTTCTCAGGCAGAGGTGGGGTGGTTCCTCTACA
GAAC

TTTAGGGATGCTCAAGAGTGGGAGAGAAGCTCTCAATGGGGTAGATGTAATCAATTTCTTGCCAG
CACTT

CTCATTCTGGCTTGATCTCCATGCAACTGAAAGCTTACAGTGTTGAGGGGGGTTAATCAGCAGCG
GTGGG

TATTTGGACAAATCGCGTGCTCCAGAGAGGTGGGGATGTGGTGCACTTGGACATTCAGAGACAT
GGCTTT

GTGTTTTACCCCTGGAGGCAGACATCTAGTCTGGGTGAAAGGTGGGGGGTGCTCTGCAAAGGA
GCTGATG

ACCACAGAAGCGGTGTGTGGACCCTGTAGTGAGAACCAGCAGTTCCTGAAGGAGGTGGTGCA
CAGCGTGC

TGGACGGCCAGGGAGTTGGCTGGCTCAACATGAAAAAGGTGCGCCGGCTGCTGGAGAGCGA
GCAGCTGCG

AGTCTTTGTCCTGAGCAAGCTGAACCGCATGGTGCAGTCAGAGGACGATGCCCGGCAGGACAT
CATCCCG

GATGTGGTCAGTGTTGGGGGTAGGGAATCGGAGTAACTGGAGAGAGGGATGTGAACACCACAG
GCAATTT

GCCAACGCTAGCCCCTGGGTTATTGTTGCAGGAGATCAGTCGGAAGGTGTACAAGGGAATGTTA
GACCTC

CTCAAGTGTACAGTCCTCAGCTTGGAGCAGTCCTATGCCCACGCGGGTCTGGGTGGCATGGCC
AGCATCT

TTGGGCTTTTGGAGATTGCCCAGACCCACTACTATAGTAAAGGTAGGGATCGTACTTGCTGGGCA
CCACG

CCATCCCTAACTCTGCCACTTGTCTCCTGAAGAGAAAATATATGTGTACCCAGGACACGTTTCCC
AGTGC

CACGCTGCTTCCCATTAGGCTTTGGGATTTTATTTTCTAATGCATGTTTATACGCAGTGGGTATTCTT
TG

TTACGAAAAATAAGACATCAGCTCATCTTTCCTTGAACTTCTTCCATTCCGCACCCTCCTGTATCCT
GGC

TCCTCCCACCTCATATCTGCGCCTTGATTCCTACTCACTACTTCTCTTGACCTCTTGATATTTTTCC
CTC

AGAACCAGACAAGCGGAAGAGAAGTCCAACAGAAAGTGTAAATACCCCAGTTGGCAAGGATCC
TGGCCTA

GCTGGGCGGGGGGACCCAAAGGCTATGGCACAACTGAGAGTTCCACAACTGGGACCTCGGG
CACCAAGTG

CCACAGGAAAGGGTCCTAAGGAACTGGACACCAGAAGTTTAAAGGAAGAAAATTTTATAGCATCT
ATTGG

TACGTATCAGTGTGTTTGGTGTGGTGGAGGGGTCCTGGCTTCTTAAATATCCCTTTCTCCTCAATTC
ATC

CAGAATCCTGCCTTTCTCTGCCCCATGCTTTGCTTCTTAGAGAGGGAAGGGGGCCTGTGTCCAT
TTCTGG

TGGTGTTTCTTTGTACTTCTCTAGTCTTTATGCTTCATCCACAGCCACCCCCCACCCCGTATCTTAG
ATG

AAGAATGGTAAGTTTTGTTTAGAGGGCCCAATGAGGCTTACTGCAGGGGGTGTGAGCTGTACTCC
TGGCC

CCTCCTTTTATTTACGTCTGCATTTCTTCACATAGTGGAGAGAATTCCTGGAAGTGCTCAGGAAGTT
TAT

AGATGCATTTTTGGGCTATAAAATTATGACATTTGTGTCTCTGAAGCCAGTGTCCTCCCATCCAACC
ATT

ATACTCCTCTGGAATGTGCTCCTCTGGGGCATGCCTGGCAACCCCATTTCCCTGGGTTCTGGAT
CTTTGA

GGTATAGGTTTCCAAGTCAGTTCTTTAAATGCTTCTTCACTGGACTTGATTATAATCTCGTTTACATTT
G

GTGTTTCTCCGTTCCCACAAAGGCAGTGCCTTAGTGAGCTTTCTAGGCATCACCTCTGTTTGGCT
CACTC

CCTGCTTGACCTTGAGTCATCGAGGTTGGATCTTTCTTTTTCTGGTTCTAGGTCCCTAGGCACTCT
GCTT

CCAGCTGACATCTCATTGCCATGGCCATTAGCAGTTGCCATATGCTCCCATAAATGGTGACATGG
AACCA

GATTCCTTTTTAGGGGAGCTACACTTCCCTAGCCAGCAGAATATTATTCTAGTAGCAGTCAACTCC
TGGC

TTTCTCCTGCCTCATGTGATGTTGTAGAAAATGTACTGACCTGCTAGTCTGGAGATCTGGAGTTTTA
GTC

CCATATCAGCCTCCAACTAGGAATGCAACTTTGGGCAAATAACTTCACCTCTCAGGGTATCTGTC
TCTTC

ATCTGGGAAATGAGGAAATCATACTAAGTACACTCTGAAGTTCCTTCCAGGAAAAGAATTGCTGTG
GTTG

CCATTCTGTCTGCTTTTCCCTATGAAGATGCTGGTCTTGACCATTTTATGTTCTCTGTCACCTCAACT
GG

AGGCATTTTTCCAGATCACTGGTGGGTAGGGCAGGTAGGGGTGAGTTGGTTTCTTGAGGAGCAA
TGAGGA

GGGATGCTAACTGCCTGCATGAATTGGCCTTTGTTCAGTCCAGTGAATCAAGCAAAGATTGGTGT
TCTTC

CTTCTGAAACTCTGGCTGCTTGAGGTGTTCATCTGTCTCATCTGGAATCTGAAAAATTGCCCTTCC
CCAG

CTCATTTGGTAGAGAGAGTGTGTGGCATGGTGGGAGTTTAAATGGATCCTAAGTTACATGATCTTGA
TCA

AGTGCTCCAGCCTCTTTAGGACCAGTTCCTCATGCAAAAATGAAGGAGCTTTGTTAAGGGCACTT
CCAGC

TCTAACATTCTGCAGGGTTTTTACTGCCACATTGGGATTGGAATTAGAAATTTGGAATCTGTTCATG
AGT

TTGGGTGGAGGGGTTAGATCTCTTGTTTTCTGCCCCTTCTCCCCATGAAGATATCTTTGTATCTCTT
GGT

TGGTAATCCAGTGCCTGAGACTGAATCGACTTGGGTGGGTGGGTTTCCATTTCGTCTGTCTGACC
AGCCT

CTGACTTTCTGTCTTTCTCTCCTGCTTGCATTGCATCTGGGGTAGAATTGTGGAACAAGCACCAGG AAGT

GAAAAAGCAAAAAGCTTTGGAAAAACAGAGTAAGGAACAAATGCCCTTTCCTGTTCCCAAGTCC TCCATA

CCTGCCAACTCCCCAGGCCCAGTTGGGGCAGGCTCCCTTTGTCAGCCCCACCCCAAATTTGC TCTTAGAG

CGTGTTTGGAATGGCAGCAGGTAGGTAGCATGTGACTCCTAGGTGTGTGTAGGCCGCTTCTTTCC CCCTC

CCCTCGTGTCAATCAGGCTATAACCAGGTGTCTACTGAATACAACTCCCCAGAACTTGGTTCTCT GTTGC

TTCAGAGTTGGTGGGGAGAATGTTTGCCTATTTTAGTGCCCCCCATTGGCTGATATCTCCCACAC CTGTT

AGAAGGCCTGTGGGCACAGTTGGCAGAGATGGTTGCTGTCCCTCCATGTTGAGTCTGTAGCTGC CCCACT

CTCTGGTGGCTTGCCTAGGCACGCTTTCTTGCTTTCTGTCATTCTGTCTTTCTGCCATACCATTGTG GGT

GGGTGGGGGACAAGGGGGGTGGTTTTCATCTCAAACATAGAAGTTGTAGGATGGAGGTAGGCA GTGCCCA

CCAAAACAGCTGGTGTTTCTTATCTCATCGGGCCCAACCTTTTCTACCTCCTTTAGACACATTTGTT TGG

GAAGCATTAAGGGACCTGGGGACCATTAAATGGTTTCAGGTACCAGTGAAGCTCAGACTCTTGA GTGCCA

TGGAATGTGGTTTAGACATTTGTTCGTGGAGCTTTTCTTTTTTTTTTTTTTGAGATGGAGTCTTGCTCT

GCTGCTCAGGCTGGAGTGCAGTGGTACGATCTTGCCTCACTGCAACCTCTGCCTCCAGGGCTC AAGCAAT

TGTCGTGCCTCAACCTCCCGAGTAGCTGAGATTACAGGTGTGCATCACCACGCCCGGCTAATTT TTGTAT

TTTTTGGTAGAGACAGGGTTTCATCATGTTGGCCAGGCTGGTCTCAAACTCCTGACTTCAGGTGAT CTAC

CTGCCTCAGCCTCCCAAAGTGCTGGGATTACAGTCGTGAGCCACCATGCCCAGCCTTGTGGAG CTTCTCT

GTTTTAAGATGATTTGCTCAGAATTTTCTCAGGTTGAGAAGATGGCTGGAGAACCCATTGAGACCA
GGTC

TGATTTGTCCTACTTGGTTCTTAAGGCATCTGGGGTCCTCTTTTTCCAGTGGGTCCTGAGTGGGGG
GTAG

TCCTTGGAAAGGTGACTAGGATGGGCTGAACGGGTCTGGGAACAGCCTGTGCTGCCGTCCCA
CCCCCTTT

ACCAGCCTGCCCCTAGCCTTTGTGCACGGAGTAACAGAAGTCTTCCCCTACTCAGGGCCTGAA
GTAATCA

AACCTGTCTTTGACCTTGGTGAGACAGAGGAGAAAAAGTCCCAGATCAGCGCAGACAGTGGTGT
GAGCCT

GACGTCTAGTTCCCAGGTTTGTGACAACCTTGTTGAAATTTGCAAGTATTAAATATGCTGTCTCAGA
ATA

CTTCTTTAAGTTAAAATAGAACAGTCAGACAGCCGGGCACAGTGGCTCATGCTTGTAATCCCAGC
ACTTT

GGGAGGCCGAGGCGGGTGGGTCACCTGAGGTCGGGAGTTCGAGACCAGCCTGACCAACATG
GTGAAACCC

CGCCCCTATGAAAAATACAAAATTAGCCAGGCATGGTGGCGCATGCCTGTAATCCCAGCTACTC
GGGAGG

CTGAGGCAGGAGAAATGCTTTAACCGGTGGGCGGAGGTTGTGGTGAGCCGAGATTGTGCTGTT
GCACTCC

AGCCTGGGCAACAAAGGTGAAACTCCATCTCAAAAAAAAAAAAAAATAAAAATAAAAATAAAATAG
CCAG

GTGCGGTGGTTCATGCCTGTAATCACAGGACTTTGGGAGGCCGAGGCAGGTGGGTCACTTGAG
GTCAGGA

GTTGGAGTTGGAGACCAGCCTGGCCAACGTAGTGAAACCCCGTCTCTACTAAAAATACAAAAGT
TAGCTG

GGTGTGGTGGCGGGCACCTGTAATCCCAGCTACTTAGGAGGCTGAGGCAGGAGAATCACTGG
AACCCGGG

AGGCAGAGGTTGCAGCGAGCTGAGATTGCGCCACTGCACTCCAGCCTGTGCCGCAGAGTGAG
ACTCCGTC

TCAAAAAAAAAAAAAAAAAAAAAAAATGAACAACCTCAAAACAATATAAATAACTAATGATAAGGGAATT

ATTAAATAAGTATAATAGATCCATGTAATGCAATATTAGTATCCATTAAAGATTGTGTTTTCAAAAATTT

AGTGACATGGGAAACTGTTTAACAAAGCTACCTATAGTGTGACTCCAATTTTATTTTTAAAATGTATTGA

GAAAAAACTGAAAGGAAATATACTAAAAACAGTGGTTAACGTGGGAGGTAGATTTACAGCTTGTTCTTAT

TTCCTTATTCTTTCATGTATTTTCTACAGTGAGCATGTACTTCTTTAACGCATTGTTTTTTGTTTTTTTG

TTTTTTTTTTTGAGACAGGGTCTCATTCTGTCGCTGCAGCTGGAGAGCAGTGGTGTGCTCTCTGCTCACT

GCAGCCTCAAACTCCTGGGCTCAAGTGATGCTCCCACCTCAGCGTCCTGAGCATCTGGGACCGCAGGCAC

CCACCACCATGCCTGGCTAAGTTTGTGTATTTTTTGTAGAGATGGGGTTTCGCCATGTTGCCCAGCCTGG

TCTCCAACTCCTGGGCTGAAATGATCCTCCCACCTCAGCCTCCCAAAGTGCTAGGACTACAGGCGTGAGC

CACTGTGCCCGGCCTATTTCTTTTTTTTATTACATAAGTTGTACATATTTTATATTCTGGAAACAAAAGT

TGCTCGTGACTTCTCTAAGGATGATAACAGAAGGGTGGGGATGAGGAGAAAGAAAAAGGTACAGTATTTC

TGTGGGAAACTGAGAGGAGATTGGACACCTCCCCTAATGTTCCTGTGTCTTGTTTCAGAGGACTGATCAA

GACTCTGTCATCGGCGTGAGTCCAGCTGTTATGATCCGCAGCTCAAGTCAGGATTCTGAAGTTAGCACCG

TGGTAGGGGAACACCACACTGGCATCTTGGTGGGTGGGGTGTGGATTTCCCCTTTGGAAAAGGGTGCTAC

TTTCTCTTTGGAGGCTGCTCTGAGTCTCAGGTCAGCATGGTTCTCATGGTGGAGATGTTTACCATCATAG

GTGTGTGTATACGAGATTTCACCCAGAGCTTCTCATCTTATAAAGAAGGTACTTCCAGAGGCTATCTGAC

ACACTTTTGAAATTTAATACTTTTTTATGGTGGCAGGGAGCTCTAACAGCTTGGTATTTCTGGGGCC
CAT

CCCTGGGGACTGTCTCTTACTACCTTTTTTCCTTTCCAGGTGAGTAATAGCTCTGGAGAGACCCTT
GGAG

CTGACAGTGACTTGAGCAGCAATGCAGGTGATGGACCAGGTGGCGAGGGCAGTGTTCACCTG
GCAAGCTC

TCGGGGCACTTTGTCTGATAGTGAAATTGAGACCAACTCTGCCACAAGCACCATCTTTGTAAGCT
TTGTT

TATTAACAAAAGAAAACCATTTCTTTAATGGGGGAGGGAAGCAGGCAAGAAAAGAATGAAAGTTA
AGAGA

CGCTAAAGAGGTAAAGGTTAAATAAGGAAGAGGTAATCTTCTTATATTTGGCTTTAAGGAAAACATT
TCT

TAAGGCTTTGTTCAGAGTCATTTGACTCACAATCTGGGGCTTTTAGAACCCTCTCCTGAATCATTGA
TGA

GTTAGGCTCCGTGCCCAGTCACGTAAGGGCTTTGAATCCTATGTAGTATTAACCCCCAGGTTTAA
CACAG

GCAAAGTAATACCTTTGTGGCAATGTCAGGACAATTACAGCTCTCGTTTAGTACCAGTCTGCCTAT
TTCT

ATGGACTATCCAGTTTTATTTTGGTCACTGCTCTATTTATAAATAAAATAGCATTTTATTTTAGTATATT

CCTAACAGTTATTTGAAGTTTTCATTCATTAATAAACATCCATGAAGTGATTTACTAAGTGCCAGATTT
T

GTGCTAAAGAAACAGGGATGAAAAAGGCATGGTTTCTACTCAAGAAGTTTGTGATTCACTATATTG
CTGT

GAGTAGTCTCTTCTAATAGGTGAGGGGAACAATGCCTTAGGAAAAAAGAACTTCCCTAGATCACA
CAGTA

AGCCCATGATCAGCTGGAAGGAGATGAATTCTTTTTAGTCCTTGCCGTAGACATCTGTTTTTTGTTG
TTT

TTTTTTTTTTTTTTTTTTACACAGGGTCTCACTCTGTTGCCCAGGCTGGAGTGTAGTGGCACAGTCA
CTG

CTCAACTGCTCACTGCAGCTTTGACCTGTTAGGCTCAAGTGATCCTCCCACCTCAGCCTCCCAA
GTAGCT

GGGACTGCAGGCATACACCACCATGCCTGGCTAATTTTTTAGTTTTTTGTAGAGATGGGGTTTCCC
TCTG

TTGTCTAGGCCAGTCTCAAACTCCTGGGTTAAGTGAGCCTCCTGCCTTGGCCTCTTAAAGTGGC
ATGAGC

CACTGTGCCCAGCCTCTGCTTTTTAATGTGTATGTGCCTCTATAGGCTGTGTTAAAACACTGATAGA
CTT

GTTACTTTATTAAACAGAGATGAGAATGGATGTTCTCTACAAGAGCTTATACGCATAGGTCCTTATCT
TT

AAGGCCTATGGGGAGAAAGGAAGATAAAAGTTAAATGAAACCAACTATTTAAAACTGGTTATCATT
CCCA

CCCCCAGAGCCTTCTGTATGTGTGTTTTTAATAGAGTCAGTGACCTCCCAGAGTGGAGATTGGGA
ATAGG

ACTGAGAGGCTACTCAAGTTAATGGGCCTTAACTCTCTCTCAGTCTGAACAGACTGATTGGCTGAT
TGAG

TGATTGATTTTTCTTTTGTGCTGACAGTTTATTAGGGGTTTTCTTTTCTTTTCTTTTTTTTTTTTTTGAG

ATGGAGTCTCGCTGTGTCACCCAGGCTGGAGTGCAGTGGTGCGATCTCGGCTCACGGCAAGCT
CCGCCTC

CTGGGTTCACACTCTTCTCTTGCCTCAGCCTCCCAAGTAGCTGGGACTACGGGTGCCCGCCAC
CACGCCC

AGCTAATTTTTTGTATTTTTAGTGGAGATGGGGTTTCACCATGTTAGCCAGGACGGTCTTGATCTCC
TGA

CTTCGTGATCTGCTCGCCTTGGCCTACCAAAGTGCTGGGATTACAGGCGTGAGCCACTGCTCC
CGGCCAA

GGGTTTTCAATAATGTTTTCTTTTCTTTTCTTTCTTTCTTTCTTTTTTTTTTTTTTTTTGAGATAGAATC

TTGCTCTGTCGCCCAGGCTGGAGTGCAGTGGCGCGATCTCTGCTCACTGCAATCTCCACCTCC
CAGGTTC

ACACCATTCTCCTTCCTCAGCCTCCCGAGTAGCTGGGACTACAGGCGCCCACCACCATGCCC
GGCTAATT

TTTTGTATTTTTAGGAGAGATGGGGTTTCACCGTGTTAGCCAGGATGGTATCAATCTCCTGACCTTG
TGA

TCTGCCCACCTCGGCCTCCCAAAATGCTGGGATTACAGGCATGAGCCACCGCGCCCGGCCTT
CTTTACAT

ATTAGTTCAACCTTCACCACAAAATCTAACCATGGATCGTAAAAATTAAATTGGCAACGGGGCTAG
CCTG

CTATACTTCCTGTTTACTTTTAGATGCTTTTCAAACCAATAAGGATGACATTTGTGGTGGAAGGAGC
CAG

AGTAATAGGAAAATTACTTCTCTTCGCTTAGAAGAAAATCCTCCACCTTGAGCATCTTCTGAACTTT
TTA

CTATAAGACATCTCAGTGCTTGCTTGTACTCTTCAGCCGCATAACTGTGAAGATAGCAACCATCAG
TTTC

TCATAAATGGAAGCGGCAGTGAGAACCGTCTACAGCAGTTTGGCTTCAAGGCCTGTGTAGAGGC
CCATTA

TTCCAAAACACCTGTTAGCCATTTGTATGTCTTCTTTTGAGAGAGATCTATTCAGCTCATTTGCCCAT
TT

TTAAATAGATTATTTGTGTTTTTTTGCTGTTGAGTTTTTTCTTTCTTGTATATTCTGGCTATTAATCCCT

TGTCAGATGAATAGTTTGTAAATATTTTCTCCCATTCTGCAGACTGTCTCTTCACTCATTTCATCGTGT
C

CTTTGTTGTGTAGAAGCTTTTTAGTTTAATATAATCCTGTTTTTGCTTTTGCTTTTGTTGCCTGTACTTT

TGAGGTCTCCATAAACTTTTTGCCCAGACCAATGTCCTGAAGCATTTCTGCTACGTTTTCTTCTAGT
AGT

TTTATAGTTTTGGGTCTTACAGTTAAGTCTTTAATCCATTTTGGGTTGATTTTTGTATATGGTGAGAGCT

AAGGGGTCTAGTTTTATTTTTCTGAATAATGGATATCAAGTTTTCCGAGCAGCATTTATTGAAGAGAC
TG

TCCCTATCCCCAGTGAATATTCTTGGTTCCTTTGTCAAAAAACAGTTGGCTATAAATATATGGATTTC
TT

TCTGGGTTCTCTATTCTGTTCCATTGGTCTATGGGTCTGTTTTTTATGCCAGTAGCATGCAGTTATGG
TT

ACTATAGCTTTGTCTTATATTTTGAAGTCTAGTAGTGTGATTCCTTTTTGTCCAGGATTGTTTTGGCTAT

CTGGGGTCTTTTGTGGTTCCATATGAATTTTAGGATTTTTTTTCTATTTCTGTGAAGAATACCGTTGGT
A

TTTTGGTAGGGATTGCATTGAATCTGTAGATCACTTTTGGTAGTATGGTCATTTTCACAATATTCGTCC
A

ATCCATGAACATGGTATATCTTTCCATTTTTTCCCTATACTCTTTCTTACATCAGTGTTTTTTATTTTTC

CTCATGGAGATCTTTTACCTCCTTCATAAAAGATAAATTTCTCCTAAGAATTTATTCTTAGGGCATTAA
T

TTGTGGCTATCGTAAATGAGATTGCCTTCTTGATTTCTTTTTCTGCTATTTTATTGTTGGTATATAGACA

CACTACTGATTTTTATGTTGATTTTGTATCCTGCAAGTTTATTGAATTCACTTATTAGTTCTGAGTTTTC

TAGTGGAGATTTTAGGGCTTTTTTTTTTTTTTTTTTTTTTTTTTTAGATGGAACCAGGCTCAAGCAGTC
C

TTCCATCTCAGTCTCCCGAGTAGCTGGGACTGTAGGCATGCACCACCGTGCCCAGCTGATTTTT
GTATTC

TTTGTAGAGACAAGGTGTTGCCATGTTGCCCAGGCTGGTCTAGAACTCCTGGGCTCAAGCAGTC
CACCCG

CCTTGGCCACCCAAAGTGCTAGGATTAACAGGTGTGAAGCACAACACCCAGCCTAATTTTAGGG
ACAGTT

TGACTTCCTTCTGTCCAAGTTGGATGCCCTTTATTTCTTTCTCTGGCCAATTGCTTTGATTAGCATTT
CC

AGTTGAATAAAAGTGGTGGAAGTAGACATCCTTGTCTTGCTACAGATTGTAGAGGAAAAACTTTCAT
CTT

TCCCCATTCAGTATGATGTTGGTTGTGGGTTTGTCATATATGACTTTTATTGTTTTGAGGTATGTTCGC
T

CTGTACCTAACTTGTTGAGAGCTTTTATCATGAAGGAATGTTGACTTTTATCAAATACTTTTGTATTTG
G

TATTCGTTCTTCTTTAAATGTTTTTAGAATTCTGCATTGAAGCTATTGGGTCCTGAGGCTTTCTTTTCTT

TTTTCTTTCTTTTTTTTTTTTTTTTTTAAGACGGAGTCTTGCTCTGTTGCCCAGGCTGGAGTGCAGTTG
T

GCGATCTCGGCTCACTGCTACCCCCGCCTCCCGGGTTCAAGCAGTTCTCCTGCCTCAGTCTC
CCAAGTAG

CTGGGATTACAGGCGCCCACGACCACGCCTGGCTAATTTTTGTATTTTCAGTAGAGACAGGGTTT
CACCA

TGTTGGCCAGGGTGGTCTCGAACTCCTGACCTCAAGTGATCTACCCCCCGCCCGCCCCAAAG
TGCTGGGA

TTACAGGCGTGAGCCACTGCGCCCAGCTTTTTTTCTTTGAGACAGAGTCTCGCTCTGTTGCTCAG
GCTGG

AGTGCAATGGTGTGATGTTGGCTCACTGCAACCTCTGCCTCCCAGGTTCAAGCAATTCTCATACC
TCAGC

CTCTTGAGTAGCTGGGATTACAGGTATTCACCACCATGCCTGGCTAATTTTTGTATTTTTAGTAGAG
ACA

GAGTTTCACCACGTTGGCCAGGCTAGTCTTGAACTCCAGGCCTCAAGTGATCTGCCCGCCTCA
GCCTCCC

AAAGTGCTGGGATTATAGGCGTGAACCACCGTGCCCTGTGATGGGAAGCTTTTTATTACAGATTC
AATCT

CATTACTTGAAATTGGACTATTCAGGTTTCTGTTTCTTGGTTAAATCTTGGTAGGTTGTCTATGTCTAA
A

TTTATCCGTTTCTAATTTGTTGGCATATAGTTATAGTCTCCAGTGATTTTTTGTTTTTCTGTGATGTCAA

TTGTAATGTTTACTTTTTTGTTTTTGGTGTTTTCTTGGTGGAGGTCTTTTCACTCTCTCTCTTTTTTTTT

TTTTGGTTAGTCTAGCTCTAATGGTTTGTGGATTTTGTTTATCTTTACAAAGATGAACTTTTCATTTTGT

TGATTATTAATTTATTTATTTTGAGATGGAGTTTTGCTCTTGTTGCCCAGGCTGGAGTGCAGTGGTGC
AA

TCTTGGCTCACTGCAGTCTCCACCTCCCAGGTTCAAGTGATTCTTCTGCCTCAGCTTCCCAAGTA
GCTGA

GATTACAGGTGCCCACCACCACAACCGGCTAAATTTTTTTTTGTATTTTTAGTAGAGATGGGGTTTT
GCC

ATGTTGGCCAGGCTGGTCTCAAACTCCTGGCCTAAGGTGATCCACCCATCTCAGCCTCCCAAA
ATGCTGG

GATTATAGGCATGAGCCACCACGCCCGGCCTATTTTTTTAAGTCTCAAGTTTTATTTCTGCTCTGAT
CTT

GATTATTTCTTCTAATTTTGGGTTTGGTTTGTTCTTGCTTTCACAATTCTTTGAGCTGCATCATTAAGTT

GATTTGAAATCTTCCTACTTTTTTGATGTGACGTTTATTGCTATAAGCTTCTTTCTTAATACTGCTTTTG

GTGTATCCCATAGGTTTTGGTATGCTGTGTTTTTATGTTCATTTGTGTGAAGAAATTTTTAAATTTCCTT

CTTAATTTCTTTGTTGACCCATTGGTCACTCAGGAGCATATTGTTTAATTTCCATTTATTTGTATGATTT

TAAAAGTTTCTTGTTATTTATTTCTAATTTTCAGAGCACAGTGTTCCTTTGTGGTCTGAAAAGATACTTG

CTGTGATTTCAGTTTTCTTAAATTTCTCATTATACTTATTTTGTGGCCTCATATGTGGTCTGTCCTAGAG

AATGTTCTGGGTGTTGATGAAAAGAATGTGTATTCTACACCTGTTGGATGGAATGTTCTGTAAATGTC
TG

TTAGGTCCATTTGGTCTAAAATGCAATCTAAATGTAAAATGTAGTATAAATCCGGTATTTCTGTGTTGA
T

TTTCTGTCTAGATGATCTGTCCAATGCTGAGAGTGGGGTATTGAAGTCCCCAACTGTTATTGTATTG
GTG

TGTATCTCTCCCTTTAGATCTAATAATACTTGCTTTATATAGCTGTTCTGTGTTGAATGCATATATATTT

GCAATTGTTATATCCTCTTATTGTTATTACATAATTACTTTCTTTGTCTCTTTTTAGGTTTTTTTTGTGT

GTGTGAGACAGAGTCTCACTCTGTCGCCCAGGCTGGAGTGCAGTGGCGCAATCTCCGCTCACT
GCAACCT

CCACCTCCTGGGTTCAAGCGATTCTCCTGCCTCAGCCTCCCAAGTAGCTGGGACTACAGGTGT
CTGCTGC

CATGCCCAGCTAATTTTTGTATTTTTAGTAAAGACAGGGTTTCACCATGTTGGCCAGGCTGGTCTC
AAAC

TCCTGACCTTGTGATCTGCCCGCTTCGGCCTCCCAAAGTGCTGGGATTACAGGCGTGAGCCAC
CGTGCTC

AGCCTCTTTTTAGTTTTTGACATAAAGTCTGTTTTATCTGATATAATTATAGCTACTCTTGCTTGCTTTT

GGTTTCTATTTGTGTGAAATATCTTTCTCCATCCCTTCACTTTCAGTCTGTATGTGTCTTTGCTAGTGA
A

ATGAGTTTCTTGTAGGCAGCATATAGTTAGGTCAAGTATTTTTTTTTTTTAATCCATTCAGCCAGTCTA
C

CCCTTTTAAGTGAGGGAATTTAATCTATTTACATTCAAGGTTGTTATTGATAGTGTGGACTTACTTCTG
T

TGTTTTGTTAATTGTTTTCTGATTATTTTGTATGTCCTTTGTTTCTTCCTCTCATTTATCATTATGGTTT

GGTGGTTTTCTGTAGAGATAAGGTTTGATTCTTTTCTTTTTCTCCTTTGTGTGTCTGTTCTACAAGTGG
A

TTTCATACTTTTGTGTATTTTCATGATAGTAATTATCATCTTTTTACTTCCAGATGTAGGACTCCCTTGA

GCATGTCTTGTAAGGGTGTGATGACTTCCTCATTTTTTTTTTCATGTCTGGGAAAGACTTAGTTTATC
CT

TCATTTCTGAAGCACAACTTTGCTGGGTATAGTATTCTTGACTGAGATTCTTTTTCTTGCAGCGCTTT
GA

ATATATCATCCCATTCTCTACTGGCTTGTAAAGTTTCTGCAGAGAAATCTGTTAGTTTAATAGAGATTC
C

CTTAAATGTGACTTAACACTTTTCTCTTGCTGTTTTTTTTTTTTTTTTTTTGAGACGGAGTCTCACTCT

GTCACCCAGGCTGGAGTACAGTGGTGCAATCTCGGCTCACTGCAAGCTCCGCCTCCTGGGTTC
ACACCAT

TCTACCTCAGCCTCCTGAGTAGCTAGGACTAAGGTGCCTGCTACCATGCCTGGCTAATTTTTATGT
ATTT

TTAGTAGAGACGGGGTTTCACCATGTTAGCCAGGATGGTCTCGATCTCCTGACCTCGTGATCTGC
CTGCC

TCGGCCTCCCAAAGTGCTGGGATTACAGGCGTGAGCCACTGCACCCGGCCTTCTCTGGCTGTT
TTTAGAA

TTCTCTCTTTGACTTTTGACAATTTGACTGTAATGTACCTTGGAGAGGCCTTTTTTTTTTTTTTTTTTGA

GACAGTCTCACTCTGTCACCCAGGCTGGAGTGTAGTAGTGTGATCTCAGCTCACTGCAACCTGC
GCCGCT

TGGGTTCAAGCTTTTCTCCTGCCTCAGCTTCCTGAGTAGCTGGGATTACAGGCATGCACCACCA
CGCTCG

GCTAATTTTTTTGTATTTTTAGTAGAGATAGGATTTCACCGTGTTGGTCAGGCTGGTCTCGAACTCCT
GA

CTTCAAATGATCTGCCCGCCTTGGCCTCCCAAAGTGCTGGGATTACAGGCGTGAGCCACCGCA
TCTGGCT

GGAGAGGACTTTTTTGGGTTGAATCTGTTTGGGGACTTTTGAGCTTCCTGGATCTGGCTGTCCATC
TCTC

TCCCCAGACTTGGGAAGTTTTCAGCTATTATTTCATTAAATAAGTTTTTATGCCTTTCCCCTTCTCTC
T

TTGGAAATCCAATAATATGAATATTTGTTTACTTAATGAGATCCCCTAAGTGTTGTAGACCTTCTTGAC
T

GTTTTCCATTCTAACTTTTTTCCCTCTGACTAATTTCGAATGACTTATCTTCAAATATAGAGTCATTCTT

CTGCTTGATCAAGTCTGCTGTTGTAGTTCTCTATTGTATTTTTTCCTTTTTTTTTTTTTTGAGACGGAGT

TTCGCTCTTGTTGCCTAGGCTGGAATGCAATGGCACTATCTCGGCTCACCGCAACCTCTGACTC
GTGGGT

TAAAGCAATTCTCCTGCCTCAGCCTCCCAAGTAGCTGGGATTACAGGCATGCGCCACCACACC
TGGCTAA

TTTTGTATTTTTTAATAGGGACGGGGTTTCTCCATGTTGCTTCGGCTGGCCTTGAGCTCCCAACCT
CAGG

CGATCCACCCACCTCACCTCCCAAAGTGTTGGGATTACAGGTGTGAGCCCCCACGCCTGGCC
CTCTATTG

TATTTTTTAAACTTTGTTCATTGAATTATTCAGCTGCAAGATTTCTGTTTTTTTGTTATGATTTTTATCT

CTTTGTTGAATTTCTAATTCATATCATGAACTGTTCTTCTGATTTTGTTGAATCATCTATCTGTATTTTC

TTGTATCTCATTGCATTTCCTTAAGATCATTACTTTTAATTTATTTTCTGGCAATTAATTGATTTCCTTT

TCATTGGGGTCTATTACCAAAGAGTTATTATGTTCCTGTGGTGGAGTCATATGTCCTTGTTTTTTTCAT
G

TTTCTTATGTCTCTGTGTTGATACCCCACATCTGGCAGAACAGTCACTTCTTCCAAACTCTCTGGA
GTGG

CTTTCATAGAGAAAGACTTTCACATGCAGTTGGGTTTTAGTGTGCCAGTTGGGAAGGGTATGGCA
ACTCT

GTTTCTGGATAGATGTAGTGGTATGTTTTCTGTGTAGCTTCTTCAGCTGCATTCAACATGAGCAATAA
CT

ATGGGTGCCTCAAGAGTCCTAGGCTATAGAAGTGTGGCAGTGGCAGTGGTGGTATATGTTGTTAAT
ATCC

TTGGTGTCAAGGAGTTTTGGAGTCCTCCTATTCTCATTTTCCTCACAATGGAGTGACTCAGCCAAG
AGAA

TCCCTCTTGGTGTCAGGTCTGACTGACATGGCCTACAAGCAGCTGCAGAGGCACTGGGTTCCA
GGTACAG

ATGCGTGGAGTAGCTGTGGAGTCAGGTTTCCAGGCTTAGGGTCTCACAAACCTATTGTGGCACC
TGGGTA

TTGGGGTACAGATTTGCTCTCTGTGGCAAGGTTGGATGTAGGTTGCCCACAGAGCCAGGATCTG
AGGCAC

CCGCTAGCAGCTCAGGCCCAGGGGACTGGCTCATAACTGTGATTCTAATACTGGGGGATAGGC
CATAGCA

CTGGCCTTACTCTGTGGAAGAAGGGGTGCTCTGGAGGTTTGGGCCTAGGGAGCAGGGTACAG
CTGCAATT

CAGGGATCTGAGCCAATAGGGCTCAGTGGCAGCTCAGGTCCCACGGAATGAGGCACCATGTA
GTAGTGAC

TCTAGACTCCAGGATGGTGGGGCTTGGCTGTATCCTAGACTCTGTGAAGCCAAGTGCAATGGCA
GCGAAT

ACCCCAGCGTGGCAGAGCACAGCTGTCATTTGGGCCCTGGGGGTCAGGGAGCAGCACAGCA
ATGACTCCA

CTCCCCAGGAAGAGGGTATCTTAGCAGCTCAGACTCTAGGGGCAAGTCCAGTTCCAGGGAAG
CAGGGTAC

TGGAGTTGTTTGGCTAGGGCAAGGTATCTCAGCTCAGCCACTGCTCTGTTTCCTGGGACATGGG
GTACTG

TGTCAGCTCAGCCCTGGAATGCGTAGCTGCTCAGCTTGGCCAGCCACCAATTCCCCTGGGGA
CAATGTGC

TGCTTTAGCACAGGCCTAGTGTGGGCATGACTGTTCTGGGTGACCCAGGCACAGTATCATTGGA
ATGCAG

GGTGCCGCTTCAAGTTAGGTGCTAGGATGCATGACTGCCCTGGGTGGCCAAGGCACCATTTCC
TGGGATA

CAGATTGCTTCTTCACCTTAGGCACCAGGGAGGCCTGACTGCTCTGAGCAGTCTAAGTACTGTTT
TCATA

GTGGATAGGGTACTGCTTCAGCTGCAGCCCAAGGGGAGTGGGGAGGGGTAGGTGGAGTGGCT
CTGCCTCC

ACTTGGCCCCTTGGGGAATCATGTAATAGCTGCTCACAGCTTGGCTTAGATTGTCAAGACATCAG
GCTAG

AGTGGTTTGTTGGTGGCTTAGCCTCAGGAATGAAGGGGAGCTGTGGCCATCTGTCCCCAGAGC
AAAACAC

AATCCCACTGTAGCTCCACTTCCAAGATGGCATAGCACAGTAGCTGCACAGGCCACAGGGGCT
GGCGCAT

AGTATTGGCTCCTTCTCTGGAGGGAGCACAGCTGTATGGACTCCAGGCAGCTCCTTCAGCTGG
GCTTAGT

GCCTAGAGGACTGTAGGGAAGCCAGTGGTGAAGTCTGTAGGTGTCCAAGGTGCTGGTGGCAGT
TTCTAGA

ATTGTCTTGCTTACTTCCTTGCTGTGGGGAGAAGTTCCTCCTTGTTCCCAGCTGATCCTAGATGGG
GGAA

GGGGGATGGGGGGGAGGCCTGATATTGCCATCTGTTCTCTATGTGGCCATCCTGAGTTTCTGTG
CTCATC

AGCATTCCTGTCACCCTTCTGATACACCCCCACAGTCTCCCTCAGTTATTTTTGTTAAAAGTATAGC
TGT

TTATTACTCTACCTCTCTTTGTGAGGGGGATGTGCTTTAGGGGCTGCTCGGCCATCTTGCTGACAT
TACT

CCCCTAGGCTGTTTTTTTTTGTTGTAACTTTTTAAAAATGTTTATTTATTTACTTTTGAGACAAGGTCTT

GCTCTGGTGCCCAGACTGCAGTGCAGGGTGCAGTCATGGCTTGGTGCAGTCATGGCTTACTCC
AGCCTCA

AACTTCTGGGCTCAAGCAATCCACCCACCTCAGCCTCCCAAGTAGCTGGGACTGTAGGCATGC
ACCATCC

CTCCTGGCTGAGATTTTTATACTTTTTTTTTTTTTTGGTAGAAACAGGTTCTTGCTATGTTGCCCAGG
CT

GGTCTTGAACTTCTGGGCTTAAGTGATCCTCCTGTTTCATCCTACCAAAGTGCCAGGACTATAGAT
GTGA

GCTATCATGCCTGGCCAAAGTGAGCTGCTTTAATAAGAGTATTTGAAATTATTCCAGTGTGTTTTAAT
TC

CAAATGTATTAAAATAGGTATGATGGAGTATCGGTCTTGCAGGAAGCCATTCAGCTTCAGTGTGCT
ACCT

TTTCTGGCATCTTTCTGGGACAAATTCGTATTTGTTCTATGCTGGTGAGCAAGGGCTCTTTATACAG
AGC

TGCTGAGTGTAGCGACCTAAATTTTTTTGCTCAAACAAGGCTGTTTTAATAAGAAATAAATTATATAAA
G

TCACGTGATCCATTTAGTAAAATATTGGGCTGTGAACCAGGAGACTGGTATTTAGTCACACTGATTA
GGT

CTTACCCAATAACCTTTGATCAGTTGCCTCTCTACTTACTCTATTTCATGTGTTGTGAAATGGCATGA
TA

TTACCTACCTCTGATCTAGTTGAAAATCTTTTTTTTTTTTTTGTGAGATGGAGTCTCATTCTGTCACCC
A

AGTTGGAGTCTCACTCTGTCACCCAAGCTGGAGTGCAGTGGTGCAATCTTGGCTCACTGCAACC
TCCGCC

TCCAGGGTTCAAGCGATTCTCCTGCCTTAGCCTCCCGAGTAGCTGGGACTATAGGTGCATGCCA
CCACAC

CCAGCTAATTTTTTGTATTTTTAATAGAGACAGGGTTTCACCATGTTAGCCAGGATGGTCTCAATCT
CCT

GACGTGGTGATCCCAACCACCTTGGCCTCCCAAAGTGTTGGGATTACAGGCGTGAGCCACTGC
ACCCAGC

TGAAAATCTTTTAATGAATAGAAAATCTACTCAGAAGTAGCAGATGAAGTTAGTGATATTTTTTAGGGT
C

AGTAAGAGTTGATAGGAAAGAAGAAATGAACATTTCTCAGTTACTGGCCAGGTGCTAGGCACTGTT
AGGT

GCCTTATATGTATCTTCAAATTTAATTCTTGTAAAGTAGTCCTATGAGGTAGGTATTATAACCTTCCTTT

GACAGATAAGGAAAATGAGGCTCAGCGAAGCTGATTTCTGTACCTAAGGTCACACAGCTAATAAG
AGGCA

CGGATCTGAACTTTGGTCAGTCTGATGGCAAACCCTGGGTTCTTTCTGCTGTGTTATGTTGCTACA
CAGA

GAATTAATCTGATTGTTGTTTTTGTTGCTTTAAAGAGAAAATAACCTTACAGAGACTAAGGAGTAAAG
AT

AAGTCAAAAATGAAAACTTTATTGTATAGTTTAGGAGGCAGTAACAGAAAGCAGCGTAAGTGGACT
TGCA

GAAGCAGGGGTACTGGAGTTTATGTTGCTAATGGATGGTGACTGGTGTGAGAGCCTCTTAGTGAA
GTGCG

TGGTTTCCTTTGTCTCTATTCATTGCTACCTCTGGCCACTGACCTATCACCTCTTCTCTCTAGGGTA
AAG

CCCACAGCTTGAAGCCAAGCATAAAGGAGAAGCTGGCAGGCAGCCCCATTCGTACTTCTGAA
GATGTGAG

CCAGCGAGTCTATCTCTATGAGGGACTCCTAGGTGAGAAACACACTGGGAAGGCCCTTTGCACT
GGAGAA

AGCTGACTCAGCCAGGGGAGAGGGTCACACTGAACAAGTAGCTGGTTGTCTTTCTGCTGATCTT
AATGCT

AACATTAGATAGCAGTTTTATACCTGAAGCAAGCGACTTACTGGGTCCAGAACAAGCAGTGGGTG
AAAAC

TGGACAAGATTGGGTTTTGGTGTTAATCAACAGCCTTTGCCTTTCTGTCTTCCTTTCTTTCCTTTCTT
GC

TACCATGGTCCTTCCTGCTCTCAAATTGGCAGGAAGGGACAAAGGATCCATGTGGGACCAGTTA
GAGGAT

GCAGCTATGGAGACCTTTTCTATAAGTAACCACATTTATTTGTGTGCTGTGTTCATCCCTCCTCCTT
GGG

AGGGACTGGGCCTTACTTGGAGGCATCTGGGGCTGGTGGCAGGCATGTTAGATCTGGGGTAGA
AAGATTG

TGTTTCCTTTGCCCTGGAATTTGTAATTATTCCCTTTTATCTGGATTTTACTCTTTATTTTTGTTTGGCA

GCTATATTCTTAAATGTTAAATAGGCCCCCTAAGAAACCTTTATTCTATGCAGGCAAAGAGCGTTCT
ACT

TTATGGGACCAAATGCAATTCTGGGAAGATGCCTTCTTAGATGCTGTGATGTTGGAGAGAGAAGG
GATGG

GTATGGACCAGGGTCCCCAGGAAATGATCGACAGGTATGGGGCTTAGGAAACCATTGGGAATC
AGCAAAC

TCAGCCTCCTCCCAGTTAGTTCTGTGGTCTCCCACTTGAGGGCTGACTTGTGCTTGGTCTTCTCC
TTAGG

TACCTGTCCCTTGGAGAACATGACCGGAAGCGCCTGGAAGATGATGAAGATCGCTTGTTGGCC
ACACTTC

TGCACAACCTCATCTCCTACATGCTGCTGATGAAGGTAATGTCAATTTTGCTGTGTCCAGCTCCG
CTGAT

CCTAGAGGGGCAAGGCTTGTTCCTGTCGCCTAAATTTCGGGGTAGCTGGGAAGCTATCAAGGTA
TCTTAA

CTTAGGGCTATCTTCTTGATTGAAAGTCTGAGGCCTCTCTGCCAGCTTACAAATGTTCCATTTATCT
GAA

GCTGCCAGCCAGTTTTACTAAGGAGTGTTGTAGGAAGAACTGATGATTCTCTTTTTTTTTTTTTTTTT
T

TTGAGACGGAGTCTTGCTCTGTCACCCAGGCAGGAGTGCAGTGGCGCAATCTCAGCTCACTGC
AACCTCT

GCCTCCCCAGTTCAAGTGATTCTTGTGCCTCAGCCTCCCAAGTAGCTGCGATTACAGGCGCAC
ACCACCA

TACCCAGCTAGTTTTTGTATTTTTAGTAGAGACAGGGTTTCACCATGTTGGCCAGGCTGGTCTCAA
ACTC

CTGACCTCAAGTGGTCCACCCACCTTGGCCTCCCAAAGCGCTGGGATTACAGGCGTGAGCCA
GTGCGCCC

GACCAAGAACTGATGATTCCATAGTATTTATTTATTTATTTATTTTCATTTTCTTTTTTTTTGAGACAGA

GTCACACTCTGTCACCCAGGCTGGAGTACAGTGGCACGATCTTGGCTCACCACATCCTCTGCC
TCCCGGG

TTCAAGCAATTCTCCTGCCTCAGCCTCCCAAGTAGCTGGGATTACAGGCATCTACCACCATGCC
TGGCTA

ATTTTTCTATTTTTAGTAGAGACGGGGTTTCACAATGTTGGCCAGGCTGGTCTCTCGTACTTCTGAC
CTC

AAGTGATCCTCCTGCCTCAGCCTCCCAAAGTGCTGGGATTACAGGCAGGAGCCACCACACCC
AGCCATTA

TTTATTTATTTTGAGATTTATGTGGTCTCTCTTTACTAAGTAGTTTTAGGGTGCCAATAGAGCTTCCCA
C

AAAATACAATGAATTAATTTTTTTTTTTTACAATAGTAGATTTTAAAACCTTAGAAAGGATGCTCAGGAC
C

AGGCGCGGTGGCTCATGCCTGTAATCCAAGCACTTTGGGAGAACAGGGCGGGCAGATCACCT
GAGGTCAG

GAGTTCGAGACCAGCCTTGACCAATATGGTGAAATCCTGTCTCTACTAAAAATACAAAAGTTAGCT
GGGC

ATGGTGGCTCATGCCTCTAATCCCAGCTACTTGGGAGGCTGAGACAGGAGAATTGCTTGAACCT
GAGAGA

AGGAGGTTGCAGTGAGCCGAGATTCCACCATTGTACTCCAGCCTGGGCAACAAGAGCGAAAAT
CCATCTC

AAAAAAAAAAAAAAAAGAAATGATGCTCAGAATTTGTTTTTCAGCACAGCCCTATATCTTACAGAAT
TTT

CTATAGTTATAAAAAGAGCCACTCATTAACAAAGTAATAGTAGATTTTAACTTGATTTGGTTGTACTTG
G

TTTTATAGCTGTGGAAGAAGTATGTTACCAAGTAGAAGAGTTCTGCTCTGAAAGGGAAACATAAATC
TGG

AGTTGATGGGTAGAAGAGAATTGTTTGGAGAACGAGCCACTGGGTTTCTCCTCCTTCTGTTCTCC
CCAAC

CTCCCTAAGTGTGAAGGGATCAGTTGATCAAATGCAGAGAATGCCCCCAGCCCCTCTTGGTGTG
CTTCAT

TTCCTGGTGTAAGTTGCACTGTACTCTGTGGAATTTGGAGGGTACCAGTTGACTCTGGCTGAGAAA
CGAT

GATGATCCCTAACCTTCAGAGATGTCTTGGCTACCCAGTCTGGTGTTGCCACTCTAAGTCATTGTT
TCTG

AATTACACTATGAAGAGCCTTGGGTGTTTTTAGAGCCCTTGTAGGGGTGAATGTTGTGGTATTGAGT
GGG

GATAGGGAGAAAGTGTCCCCTGGAACCAGAACCGCTCTAAGAGCCACTTTTCTGCAGAAGCTA
ACTAGAG

ACAGAGCCAGGACTTTTCTTCCCCTCATGGTGGCAGAATACACAGTTGAAAGTTTTCTCCCATGT
TCAGT

GATGTGGTCTTTCAGAATTCCACTTCTCTCAGATGGTGGGAAGCCCACTTGTACATTCTCTTTTCTC
TAC

CTCAGTCGGGAGGAGAGCAGATCCCTTGTTAAGAGTCATGTGTTGGCTTTTCAGGTAAATAAGAAT
GACA

TCCGCAAGAAGGTGAGGCGCCTAATGGGAAAGTCGCACATTGGGCTTGTGTACAGCCAGCAAA
TCAATGA

GGTGCTTGATCAGCTGGCGAACCTGGTAAGCACGTCTGGCCACCCCTTAGGCTTCCCCATGG GTCATTTC

TTGGTTTGTGTCACTTGCAGTCCAGTTCACCCCGTTTTTGAAAATGGAGCAGTTGTCTTTGACTGTA AAT

GAGGCATTAGTCCCTGTGTTCTGTGATGGGATTCTCTGTATAAAACCATCGTAGTGCATCAGTTTTG ATG

TGTGGTCTGTGAACTGCTGAGGGTTCAGAAGGTGAAAACGATTTCAAATGATTCTAAAATGTTATTT GCT

GTTTTCACGGGGTTACATTTGCACTGATGGTACCAACGCAGTGATGGGTAAAACTGCTGGCACTG TAACC

CAAGTGAAGACAATGGCCCCAAACTATGCCAGCAGCCATTGCATTCCTTGCCTTCATATACTCTC AGTTT

TTCGTTTTTGTTTTGTTTGTTTGTTTTTTGAGACTGACTCTCACTCTGTCGCCCAGGCTGGAGTGCA GTA

GCGCAATCTTGGCTCACTGCAACCTCCACCTCCTGGGTTCAAGCGATTCTCATGCCTCAGCCT CCCAAGT

AGCTGGGATTACAGGCATGTGCCCGCCCACCCAGCTAATTTTTGTATATACATATATATTTTTTGAG ACA

GAGTTTCACTCTCTTGTTGCCCAGACTGGAGTGCAATGGCGCAATTTTGGCTCACTGCAACCTCT GCCTC

CCGAGTTCAAGCAGTTTTCCTGCTTCAGCCTCCCGAGTAGCTGAAGCTCCTGAGGTTACAGGCA TGCACC

ACCACGCCCAGCTAATTTTTGTATTTTCAGTAGAGATGGGCATTCCCCATGTTGGCCAGGCTGAT CTTGA

ACTCCTGACCTCAAGTGATCTGCCTGCCTTAGCTCCCAAAGTTCTGGGATTACGGGTGTGAACC ACCGCA

CCTGGCTTACACTCTTAGTTTTTTAAAAATGCCAGTTGTGCTTGTAAATGTCCTTGAAGAAGCAGTG AAG

ACTGTTACTCTTATTAGATTGATTAATTATAAATGTTTTTAATATTCTTTGTGACAAAATGGGAAGTACA

CATAAAGCATCTCTGTTGCAAACCCAGTATGATGGTTGTTTTGAAGAAAAGCAATTTGCAGCCTTG ATTT

GTTCACCTTGAGTTGCAAGTGAACTTAATCGCTTTGTTCATTGAACATCATTTTTACTGGAAAGGAC
AAT

TAATAAACCATGGTTATTCAGAGTACCCAACTGGCAGACATTTTCCTTCAGTTAATCAAGTGAGCCT
GTT

GTTTCAGGGAAAACAACTGACAGTATTTGTTGCCAATGATAAGATTCAGCTTTTAAGTGAAAATAAG
AAT

TTTGGCAGAGTTTTGAACTTGAAGCTTCCCAATATTTAAAGACTTTTCTAATGAGATTGGTGGTGATA
TT

AATGAATGTGATTTTTTAAACATTGATTTAATATTACAAAATATGTCAACATTTGGAAGAGCTGTGTAAC

TCAGTGAAACACTATTTTCTTTCTTTTTTTTTTTTTGGAGACGGAGTCTCGCTCTGTGGCCCAGGCT
GGA

GTGCAGTGGCAGGATGCTGGCTCACTGCTACCTCCGCCTCCTGGGCTCCTGGGTTCAAGCAAT
TCTCCTG

CCTCAGCCTCCTGAGCAGCTGGGAGTACAGGCGTATGCTGCCACACCTGGCTAGTTTTTTTTTTT
TTTTG

TATTTTAATAGAGACGGGGTTTCACCATCTTGCCCAGGCTGTGGTCTTGAACTCCTGAGTTTAGTC
AATC

CGCCCACCTTGGCCTTCCAAAGTTCTAGGATTACAGGCACCCAGCCAACAGTATTTTCTAAATAC
CAATG

TATGATGTTGTAAATTCAAGTATGGATAAAAGATCTACTCAAAATGCAAGGCAGAGATATGGATCTTA
AT

TTTTAAATAACATTTTTTTTCAGTCTGGGGAACATAGACCCCATTTCTTTTTGTTTGTTTGTTTGTTTTT

GAGACGGAGTCTCACTCTGTCACCCGGGCTGGAGTGCAGCGGCGCGATCTCGGCTCACTACA
ACCTCCGC

CTCCCAGGTTCAAGCTATTCTCCTGCCTCAGCCTCATGAGTAGCTGGGATTACAGGTGCCCGCC
ACAACG

CCCGGCTAATTTTTATGTTTTTAGTAGAGATGGGGTTTCACCACGCTGGTCAGGCTGGTCTCCAA
CTCCT

GACCTTTTGATCCGCCCACCTCGGCCTCCCAAAGTGCTGGGATTACAGGTGTGAGCCACCGC
GCCCAGCC

AAGGGAGACCCCATTTCTACAAAAAATAAAAAAATTAGCTAGGCATGGTGGCATGCACCTGTAGT
CCCAG

CTACTTGGGAGGCTGAGGTGGGAGGATCACTTGAGCTCAGGAGGTTGAGGCTGCCGTGAGCA
CCACTGCA

CTCCAGCCTGGGTGACAGAATGAGACCCTGTCTCAAAAAAAAAAAAAAAGTGTATGGAGAATAG
GGTCTC

ACTTTGTTGCCCAGGCTGGTCTCCAACTCCTGCCTGGGCTCAAACGGTACTCCTGACTTGGCCT
CCCAAA

GTGTTGGGATTATAGGAATGAGCCACCATGTCTGGCCTAAATGGATTTTAATGTAACAATGTAAAAT
GTT

CATTGATGTGCTTTCAGATTCCACACTGCAACTAACTTTCAAAAGCTGCTTTTGCTGGGTGTTGTG
GTTT

ATGCCTGTAATTCCAGGTACTTGGGAGGCTGAGGCAGGAGTGTCACTTAAGCCCAGGAGTTCAG
AGCTGT

GGTGGGCTATGGTCATGCCACTGCACTCCAGCCTGGATGACAGAGCGAAACCTTGTCTCTAAAA
AAAAAG

AAGCTTCTGCTTCCCAAGTTCTGGTGTAGTGTTAAAGAAGAATACGTCTATTTATCTGAAAAGGCTA
TGA

AAATCCTCTTCCATTTTCCAAATACGTATCTCTGAGGTCAAGTTTTTTTCATATACCTCAACCAAAGC
AA

CATACTGCAACAGACTCAATGCAGAGGCAGATAGGAGAATGCAACTATTTGATTCTAAGCCAAAC
ATTAA

AGAGATTTGCAAAAATGTGAAACAGTGCCTTTTTTTTGAAAATATAGTTATTTTATTAAAAATGTTTATA

TTACTATGTAATGGGTTATATTAATCATTAATTGTTTTTATAATTAATCATTTGAGAAATTTCTGTTTAA

ATTTCTAGTATGTAAATATCTATAAATATAACTCACATAAACAAAAGCTCTTTGGAATCTTCAATAAATT

TTAAGAGATATAGGGCCTCTGAGACCAAAACATCTGAAACCACTTCCCTGGTGACCAGTGGCCA
GCAGAT

GAGACTGTGCTGAGGAAGCCGATATGAATTTGATTGCTGGATGGGGAATTCTTGGCCCAGAGCC
CTCTGA

GAGGGATGTATGACTGTCCCTAAAAAATCTCTCTTTCATCAGAATGGACGCGATCTCTCTATCTGG
TCCA

GTGGCAGCCGGCACATGAAGAAGCAGACATTTGTGGTACATGCAGGGACAGATACAAACGGAG
ATATCTT

TTTCATGGAGGTAGGTGCTGGTTCATGCTGGGGGCCCAAAGGGCTATTGAGAGTCACAGGGAA
CTCATAG

GACCGATGCCAGGATATTTTTCTCATTTATCTCATTCATCTTCATGATCTATTTATCATTAAATTATCAG

ATTGGTTTTTTTGTTTTTTGCGTGTGTGTTTTTTTGAGATAGTGTCTCGCTCTGTCACCCAGGCTGGA
GT

GTAGTGGCTTGATCTCGGCTCACTGCAACCTCCGCCTCCCGGGTTCAAGCAGTTCTCCTGCCA
CAGCCTC

CCAAGTAGCTGGGATTACAGGCGCCAGCCACCAAGCCCAGGTGATTTTTCTATTTTTAATAGAGA
TGGGG

TTTGATCATGTTGGCCAGGCTGGTCTCAAACTCCTGACCTCAAGTAATCCACCCACCTTGGCCTT
CCAAA

ATGCTGGTATTATAGGCATGAGCCACCGCACCCGGCCAAATTATCAGATTGTTATTCAAATAATTA
AATT

TTATATAGTAGATGATACCCATTTCTTTTTTTTTTTTTTTTTGAGACAGAGTATCGCTCTGTCACCCAG
G

CTGGAGTGCAGTGGTGTGATCTTGGCTCACTGCAGCCTCCACCTCCCAGGTTCAAGCAATTCTC
CTGCCT

CAGCTTCCTGAGTAGCTAGGATTACAGGCATGCGCCACCATGCCCGGCCAATTTTTTGTATTTTA
GTAGA

GGTGGGGTTTCACCATGTTCGTTGGCCAGGCTGGTCTTGAACTCCTGACCTCAAGTGATCCACC
TGCCTC

GACCTCCCAAAGTGCTGGGATTACAGGTGTGAGCCACTGCGCCCGGCCCCATTTCTTTTATACA
CGTGCG

CGCGCACACACACGCGCACACACATATACATAAAGATATCTATATATAGATGATATAGTCACATTGT
TCA

TAAATCAGAATAACAGAAAATAGTACACATTGGGAGGTCTTGCACTCATTCTTGTCCCCTTCCACT
GAAT

TTCCCACTCCCCTCACCCCTTCATCCTATAGACAACCACTTTTATTGGTTTCCTTTGTAAATTCCAG
TGG

ATTTTTTTTTTCTTTTTCTTTTTTTTTTTTTTTTTTTTTGAGACGGAGTCTCACTCTGTCGCCCAGGCTG

GAGTGCAATGGCTTGATCTCAGCTCACTGCAACCTCCACCTCCTGGGTTCAAGCGATTCTCCTG
CCTCAG

GCTCCCAAGTAGCTGGGATTACAGGCGCCCACCACCGCACCCAGCTAATTTTTTGTATTTTTAGT
AGGGA

TGGGGTTTCACCATGTTGGTCAGGCTGGTCTCCAACTCCTGACCTCACTGATCCACCCGCCTC
GGCCTCC

CAAAGTGCTGAGATCATAGGCGTGAGCCGGTGCACCCGGCCTTCCAGTGGACTTTTATGCAAA
CAATAAA

ATATGAACACAAATTCTTATTTTCCTCCTTTCTTTTTTTTCTTTGAGACAGAGTGTTGCTCTTTTGCTCA

GGCTGGAGTGCAGTGGTCCAAACTCAGCTCACTGCAACCTCTGCCCCTTGGGTTCAAGTGATT
CTCCTGC

CTCAGCTGCCTGAGTAGCTGGGATTACAGTCGCCCACCACCATGCCCAGATAATTTTTCTATTTTT
AGTA

GAGACGGGGCTTCACCATATTGGCCAGGCTGGTCTCAAACTCCTGACCTCAAGTGATCTGCCC
ACCTTAG

CCTCACAAAGTGCTGGGATTATAGGCGTGAGCCACCCCGCCTGGCCTATTTTTCTCCTTTCTTTC
AGAAA

GATAGCACATCTTATCCATTGCTTTATCCCTTGCTTTTTTCATTTAACATTGTCCTGGTCCTCTTCTGT
A

TCAGTACCAAGAAAGCTTTCCCATTGGTTTTATGGCTACATGTATTCCACTGTGTGACTGTGCCTTA
ATT

TATTTAACCAGTCAGTACATATGGATCCTTGGGTTGCTTCCAAATTTGCTGTTACTGATAGTCATACT
GT

GAATGATGCTGTGCCTGCTTCATTTCATACATGTGCAAGTGTGTCTAAGGATTCCCAGAAATGAGA
TTGC

TGGGTCAGAGGGACAATGCATTTGTAATTTTGGTTGATATAATTCTTAAGTGTCTCATAACACGTAAT
AC

CCCCTTTGCCATTAATTTTGTTGAAGAAATTGGATCGTTTGCCCTGTGGAGTCTCCTACATTCTAGA
TTT

TTTGCTGATTGTGTCTCCTTTGTATTAGCGTGTTTCCTGTCACATATTTCTCATAGACTGGTAGTTCAG
T

TTAGAGGTTTGATCACTTTCAGATTCACTCTTTTGGGAAGAATACTTAATGAGTGGTGCTATACGCTT
CC

TATTGCCTCATATGGTTGTCCCTCTCTTACACATCAGTGGGCTCAGATGTGGGCAACCTGATCTGC
CCAC

CAGAAAGTTCCTCATTTGTCTTCTGATTTTGCCTATGGTATTTTTTGACACACATAAATTTTTTATTTGT

ATATAACTTTTTTTTTTTTTTTGAGACAGAGTCTCTCTCTATCGCCCAGGCTGGATGCAGTGGCATGAT
CT

CGGCTCACTGCAACCTATACCTCCCGGGTTCAAGCAGTTCTCCTGCCTCAGCCTCCCAAGTAG
CTGGGTT

TACAGGTGCCTGCCACCACACCCGGCTAATTTTTGTGTTTTTAGTAGAGATGGGGTTTTACCATGT
TGGC

CAGGGTGGTCTCGCACTCCTGACCTCAAGTGATCCACCCACCTCGGCCTCCCAAAGTGCTGG
GATTACAG

GTGTGAGCCATCACGCCTGGCTACTTTTATATAATTAAATTAATCAATAGTTTCTTTTACGGCTTCTG
GA

TTTTGAGGCATGGTTAGAAAAAGCCTTACTCTCCAGGATTATAAAGGAAATTGGCCGTGTTTTCTTC
ATT

TCTGTGTTTTTAATTCTGAAACAGACCTAAGTAATTTTAAGGACCAAATACAACTCCTATATATAAAGT
A

GTAAAATTTTGCCTATGTTATCTGTTCCCCTGTGGTTTAGCTGAATTTGAATATCACATTCACAGTTTT
T

TTGAACAGGTGGTCTACCAAACGCAAGAAGGGCTTACCCTGAAGCAGAAACATACATGTCAGTG
TAGCCA

AGAAGGAGTCATTTTCATGTCACCCTTTAAAGTGTTAGAATTTGGCTAGTTGCCTCTCAGAGCCTA
GGGC

TGACTTAGGACATCTGCACCACAGCAGAAGTGGCACATGACTCTTGCCCTTTTCTTTTCCTTTTAA
AAAA

AATATCTCCAGCAGATTCCTTGAACTACTCTTACTCTCACCACCGTACAAAGGGAGCCTCATGCT
AAGTC

TCTGGAGTGTGGGACTGGACCCATTACCCAAATAGATGCCTCTTCCTCGAAGTGGTGCCATGCC
AGGGAA

GAGTGCACCATGGTCACTGTGCCATCACCTGGCTCCTGTGAGGCTATTTGATTTAGCCTTTCTCA
GAGCA

TAGCCTGCTTTTTGCATGATCAACTGGACAGCAGTTTGGGAATTTTTTTTTTTTTTTTTTTTTTTTTTTT

TTTTTTTTGAGACAGAGTCTCACTCTGTTGCCCAGGCTGAAGTGCAGTGGTACAATCTTGGTTCAC
TGCA

ACCTCCACTTCCCAGGTTCAAGCGATTCTCCTGCCTCAGCCTCCTGAGTAGCTGGGACTACAG
GCACATG

CCATCATGTCTGGCTAATTTTTGTATTTGTAGTAGAGACGGGGTTTCACCATTTTGGCCAAGCTGGT
CTC

GAACTCCTGACCTCAAGTGATCTGCCCACCTCAGCCTCCCAAAGTGCTGGGATTACAGGTGTG
AGCTGCC

ACACCTGGCCAAGGAAAATATATATATATATATAATTTTTTTTTTTTTTTTGGAGGTGGAGTCTTGCTCT

GTCACCCAGGCTGGAGTGCAGTGGCACAATCTTGGCTCACTGCAACATCCACCCACCGACTTC
AAGAGAT

TCTCCTACCTCAGACTCCCGAGTAGCTGGGACTACAGGCACGCATCACCACGCTTGGCTAATTT
TTGGTA

TTTTTAGCAGAGATGGGGTTTTGCCATGTTGGCCAGGATGGTCTTGAACTCCTGACCTTAAGTGAT
CCGT

CTGCCTTGGCCTCCCAAAATGCTAGGATTACAGGTGTGAGCCACCATACCCAGCCAAAGATTTT
TAAAAA

TTGTCTTTGTTGCCCTTTTTTTAGTTACAGAAGTAAAACAGTATATGCTGTAAATGTTTCAAACCGTGC
C

AAAGTTAAAAAATAATTAAAAGTTTTACTCACTGCCTTTCAATCCTTTGTCCCAGAGGTAACCAGTA
ATG

AAGAGTTTTATGTGACTCCTTTCAGATTATTGAAAATGCATATTTGTGCAAATATAATTTATTTATATGG

AATTTTGTTTTTGCACTTACGAAAGTGGTATCAAATTACAAATATTGGGCTGGGCATGGTGGCTCAC
ATG

AGCCTGTAATCCCAGCACATTGGGAGGCCGAGGCAGGTGGATAACTTGAGGTCAGGAGTTCCA
GACAAGC

CTAGCCAACGTGGTGACACCCTGTTTCTACTAAAAATACAAAAATTAGCCGGGCGTGTTGGGCA
GGCGCC

TGCAATCCCAGCTACATGGGAGGCTGAGGCAGGAGAATCGCTTGAACCCGGAGGCGGAGGTT
GCAGTGAG

CCGAGATCATGCCACTGCTCTCCAGCCTGGGTGACAAAGCGAGACTCCATCGCAAAAAAAAAA
AAAAAAA

AAAAATTACAAATATTGTTAAATAATTTGCTTTTTTCACTTAATGATATACCTTTCCGTGTCTTTTTTTT

TTTTTCTTTAATGGATGCTTAGCCATTAGATGTCGTATGGTGTACCATAATTGGTTTAACTAGTGCCTC
T

CACTATTTTTTAAAAAAATATTACTCATGCTGGGTGTGGTGGCTCACGCCTGTAATCCCAGCACTTT
GGG

AGGCCAAGGTGGGTGGATCACGAGGTCAAGAGTTCAAGATCAGCCTGGCCAAGATGGTGAAAC
CCTGCCT

CTACTAAAAATACAAAAAATTAGCCAGGCGAGGTGGTGGGTGCTTGTAATCCCAGTTACTTGGGA
GGCTG

AGGCAGAGAATTGTTTGAACCTGGGAGGCGGAGGTTGCAGTGAGCCGAGATTGCACCACTTCA
CTCCAGC

CTGGGCGACAGAGCAAGACTCCGTCTCAAAAAAAAAATTTAAAAAAATGAAATATATATTTATATATT
AC

TCATTAGGCCAAGCATGTTGGCTCACGCCTGTAATCCCAGTACTTTGGGAGGCTGAGGCAGGA
GGATTGC

TTGAGCCCAGAGTTTGAGACCAGCCTGGGCAACATAGGGAGACCCTGTCTCTATAAAAAATTTAA
AAATT

AGTCATACATGTGGTGATGTATGCCTGTGGTACCAGCTACTGGGGAGGCTGAGGTGGGAGGTTC
GCTTGA

GCCCAGGAGGTCAAAACCAGCCTGGGCAACATAGGGAAACCCCGTCTCCACAAAAAATTTTAA
AATAAGC

TAGGCATGCTAGTGCATGCCTATGGTCCTAGCTACTGGGGAGGCTAAGTTGGGAGGATCCTTGA
GCCTGG

GAGGTCAAGGCTTCAGTGAGCTCTGATTACACCACTTCAGTCCATCCTGGGTGACAGAGCAAAA
CCCTGT

CTCAAAAAATAATAATAATAATAATAATTGCTCATTGAATATCCTTGAACATATATTTTTGCACCCTCAC

ATGAGAATTTCTATAGGATATAGTCCTAGACATGAAAATTTCAAGTCAAGCTTATATACGTTTAAAATT
T

CGGTAGATTTTGCCAAATTGCCATCCCAAAAACTATACCAGTTTACACCTCCAACAGCCACTTAG
ACGAC

TATAACAGTATATTACAAGCATTTCTCTAGACTAGAATGCTCTAGCTTAGAAATCATGTGTTCTCCTAA
A

TGTGTTCTGCCTGCCTTTACATGATGTTCTTAAAGGATTATGAAAGGTGGAGTGGTATTGTCCATTTT
CC

CCCTGGAGATAACCACAGAGTCAGACTGGGCCTAGAACTCAAGTTTCCCATTCCTACTCTGCCA
CTTTGA

CTGATTCTTCAAAGGCTTTAGAACTCCAGTAAAGTCTTATACAGTAGCTTAAACCTGATAGTCTTGT
GTG

TTCCTTACTCACTCACCTAATATTTGTGGAGCACGTATTATATGCTAGGTACTTTGTTGGGTGTTGGG
AT

TATAGCAGTGAATAAGACAGACGTGATTTCTATTGCCCTTAGCATTGACAAGCTATTGGGGAAATC
AGAT

AATTAAAGAGTATTATAAAATATGGTGAGCATTATGAAAGAAGCTCAGGGGGGGCAGAATAAATAC
CAGA

GGGCCTAGGAGCTGTGGACAACTCCTTTGAGAAGTTGACCTTTGAGCTTAGACTTGAAGAAAGA
GTAAGA

GTTAATGGTTGGGGAGTGGAAGAGCAGTCAGGGATATTTGAGCTAGAACCAATGGGACTCAGTG
ATGGAG

TGTAGTTAGTTAGAGGGAAGAGGAAGGAGTCGGGCATCAATTCTTCATTTCAGGCTGGGGCAGC
TGGGTG

GATGGTGGTGCCATTTCCTCAAATAGCAGACTTAAAAGGAGGATCTGCCCAGGAGGAGATGTTG
AGTTCA

GATTTGGACACAATGAGTTTGAGGTGCTACATGTGAAGCATCCAGACACTGAGAAGCTCAGGAG
CTCAGG

AAGAGAGGTCTAAGATGGAGAAAGGTAAGAACCATCAACATGGTAATAATCATTAGTATCATAGTTC
TGA

TATCTAGTAGCTAATTAGGGTACTTTCTGTGTGCCTGGCTTTGTGTGCACCTTAAATACTTTATGCAC
AT

TAACTCATTTAGTCTTCATAGTAACTGTAGGAAGTTATTACTGTTACTATCCCCATCTTACCTGTGAG
GA

ATTGTGGACACAGATGAACTTGCTCAGGGTGGCACAGCTCAGTAATGGCAGAGCTGGAATCTAA
ACTCAG

GCTGACTTTAGACCTGTGCCCTTCACCATTGTGCTGTAATGTCTCCCGTAACATTTGAAGCCTTAA
GAAC

GGATAACTGGCTGCGCATGGTGGCTCACGTCTGTAATCCCAGCACTTTGGGAGGCCGAGGCG
GGCGGATC

ATGAAGTCAGGAGATCAAGACTATCCTTGCTAACACGGTGAAACCCCGTCTCTACTTAAAAAAATA
TATA

AAAAATTAGCCGGGCATGGTGGTGGGTGCCTGTAGTCCCAGCTACTCGAGAGGCTGAGGCAG
GAGAATGG

CGTGAACCTGGGAGGCGGAGCTTGCAGTGAGACGGGATTGCGCCACTGCACTCCAGCCTGG
GCGACAGAG

CGAGATCCGTCTCAAAAAGAAGAACAGATAACTTCACCTGAAAGAGAATGTTGAACAAGAAGGT
GTCAAA

GATATTTGCTTTTTAATTTATATCATCCTGTATTGTATCATTTTTATGTAATAAGCATTGTTCATGTGTC

ACTTGTGTAATTAATTTTTAAAAATATTTTTATAGAAAAAGGGTAGACCCAGGATTAGAAAGTGCATCA
G

CTCCATTTTTCTCCTTGGTTAAGTCTGTCATGATCTCCTACCTTTGTGTTTGTCAACAAAGATGATTC
CT

GAGCTTGTAAGATGGAGGCATGAGATCTCCTCCAAATACTTTAAGTCCGTAGAGCACATAAGAGG
TAATA

AATAACTGGGCCGGGCGCGGTGGCTCACGCCTGTAATCCCAGCACTTTGGGAGGCCAAGAC
GGGTGTATC

ACTTGAGGTCAGGAGTTCGAGACCAGCCTGGCCAACATGGTGAAACCCTGTCTCTACTAAAAATACAAAA

ATTAGCTGAGCATGGTGATGTGTGCCTGTAATCTCAGCTACTCGGGAGGCTGAGGCAGGAGAATCATTTT

AACCCACAAGGCAGAGGTTGCAGTGAGTCGAGATTGCACCACTGCACTCCAGCCTGGGCAACAGAGTGGG

ACTCCATCTCAAAAAAAAAAAAAAAAAAATTATAGCCAATGTGATGGTGTGCACCTGTAGTCCCTGCTACT

TTGGGGGCTGAGGCAGGAAGAACCTGGCTGGTCGAGGCTGCAGTGAGCTGAGATGGCGCCACTGCAGTCA

AGCCTGGGGTACAAAGTGAGACCCTGTCTCAAAAAAAAAAAAAAGAGGTAATAAATAACTGATTGATTTG

TGCCTCCTCCCTTATACATCGGTTCCTTCTAATGACTCCATCTCTTCCAACAATCCTTGTAGCAGGCAGC

ATGTCAGTAGTTCTGTTTTGTGTCTTCACCTAGGGAAACCATGAGCGTAAGGCAGAAAACCTGACCATGG

GGATGCCCCAGGACCACCTTTCCCTGTAGGAAACAGTCTAGGGAGAGGCTGTCAGAGACAGGAACCACTG

AGCCGCTTTGTGCACTTCTCCAGGTGTGCGATGACTGTGTGGTGTTGCGTAGTAACATCGGAACAGTGTA

TGAGCGCTGGTGGTACGAGAAGCTCATCAACATGACCTACTGTCCCAAGACGAAGGTGTTGTGCTTGTGG

CGTAGAAATGGCTCTGAGACCCAGCTCAACAAGTTCTATACTAAAAAGGTACGCAGGATCTGTGTTTGGG

TTGGGGCTAGTAGGCATTGAAGACCAAATAGTGAATTTCTCTTTGGGAAGTTAAAATTCCAAAACACACA

TCTGGAGCCACACTTCTGTCTCCAGCTGTTGGGTGGAGTACCTAAAGCTGTCTCTGTCAAGCCAACTAGG

CCTAATAGTAGTCACAATCGGTATTAACCAACCATATCTAGCATTCCTACACATCTGTACCCATGCCTTC

TTTAAAGCCATACTATTCATATGCTCCGGATCTATCATTCATAACCTCAACAATGAATAAGACATCC
GAA

AAATAGGAGGACTACTTAAAATTTTACCCCTCACTTCAACCTCCAGCCTTCAGTGGCCATTATAGA
GCAG

TGGGTGGGGAACCCTGGACAGCCCTCATGATGGGACCACTCTCCCCCTGTGCCACAGTGTCG
GGAGCTGT

ACTACTGTGTGAAGGACAGCATGGAGCGCGCTGCCGCCCGACAGCAAAGCATCAAACCCGGT
GAGGAGAG

TTTTTCCTGAGAGTGTCTTCCCTGTTTCTACCAGTCCTTCTGAGTGTCAGGGGCCTGGCAGGGAA
GTCAG

GGGTGGGATTCCCCACCTCACCAGTGAGCTGATAGCCCCCTCCCTCACCACAGGACCTGAAT
TGGGTGGC

GAGTTCCCTGTGCAGGACCTGAAGACTGGTGAGGGTGGCCTGCTGCAGGTGACCCTGGAAGG
GATCAACC

TCAAATTCATGCACAATCAGGTAGGTGCGAGCGGCAGCACGAGGCTCCCTGTCGTTCCATCTGT
AAGAAG

GACCAATGTCCAAGCCCCCAGTACTTCCCCAGCAAGCATGAGAGAGGGCCCTCTGGAGCTAG
AGGGAGAA

CTGGAGCAGCTGGACCCAGGAAAGAAACCCCAGGGGAGGGCACAGTGACGTTGGCGACCA
CCTGGCGCTG

TGAAGGTGCACCAGGGAGCCCGTGGGGGAGGCTGGGCAGGAGCGAGGCCAGGTGGCCAC
GCTGCCCCATC

CGTTGGACTCCACACAGTGTGAGTGGGAGCGTCTCACTTGGCCCTTCCCCTGCAGTGTCTCTG
CCTTCCT

CTATTCCCAGGATATGCTTCTGCTTGGTGGTTTTGCTTTGAGAGTCTAGACTGGCCGATCTCCTTG
TGTC

TTTCTGGTGTGCGTGCAGCTTGCGTGTGCGTGCGTGCGTGCCTGCGTGCTTGTGTGTAAGTAGCT
TGTAT

CTGTCCTCTCTGGAGTGTGTTTATTTGCCTTTTTCTTCTGCGGATTCTTCTTCCTGTTCTTTTCCTTCC
T

CTGAGTGTTCCCACTCTGTCCCCTGCTCCACAGGCCCCATTCCCTTTCTTTTGATTGGGGAGCA
CTTGTG

GATACATCTTTCCCTCATTGTAATTCCACCATAGGAGCTGACTTTTTTTTTCTCTCTTGGTCTTTTAA
C

ACAGTGTTACCTTTTTCTCTCAGTGGACATTCCTTAAGCTAATTCCTTTCTGAGGCCAGCGCATCAT
CCC

AGGTCCGTTCACAGCTCTGACTCCTCACACAAGTGCCTTCCCTCCTCTTTCCCTGATGTCCGGG
TTCCTC

TCCCTGAGGCTCTGAGCCAGACCCAAATGTGTGAAGCCCCACAGACCCTGGCCAGGCCGCA
GACCCTCAG

GGGAGGTGGATGCCTGACTGACGTGGCTGTCTCCCTCCCTCCGCTAGCCGTTGTAGCTCTCTTT
CAGGAA

CGTAGCTGGGCAGCTTTGGTTTAACAGGAAATGGCTGCGCAGGCTGCCAGCAGCCCCTTAGTA
TATTTTC

AATTTAGGACTTAGGGCAGTAGCCAACCCCAAAGACCTTAGGACCATCCCAGAGAGGGGTCTA
CCTAAGT

GCTTTTCTGGACCACAAAGGTGTCAGTACTTCTTGGGGAGCAGACTTCTGACCACATTTTAGCGC
CGTGG

CCTCTGAAGATGAGATCTTGCTGAGCAACGCCCAGGTGCTCTGCCCTGTTCCTCGTCCTGCCC
TTGGCAT

AGCCCCACTGGGCAGCCATCCAAGCCAGTCTGTGGTTGAAGGCTTGGCCCCTAGAAGATAGA
CTGCAGAG

AAGGGGAGGAAAGCCCGGGTTCTCAATCTCACCTCACAGTTAGTAATGAGATAGCGACCTGCC
CCCTATT

CTGCCACACAGTGTTCAGTTGGAGTGGAGGAGGCAGCAGACCTCCTGGTGGAGCTGTGCAGT
CTGGGGTA

AATGGGAAAAGCACAGGCTTCGGTGTGAGGACATGCTTGGATTTGAAGCCCAGCTTCCACGTTC
ATGGGC

AAAGAAACTTTCTGATCCATAGTTTCCTCATGTAGAAAATGGGGGTAATACTATTTACTGTGTCACGT
AG

TAGGTGCAGAGATTAGAAATCGTATTTGTAAAACCCCAAGTAGGTGCTCAGCAAGTGGTAGCTGA
TATTA

TTATCACCACATACCTCTTGGGAGATCCTGGATCCTGTTTCAAGGAGGATACCAACACCTCCCAC
CCTGA

GGACAGTCTGAGGGGAGCTTTGCGCCCGTGCCAGCCCTCTGCGCCGGAGGAGGATGCATGG
AAGGGTAGA

GACCTAATGGGCTGAGTGGGGACCCCTGGGGCACTGTGTCTTACAAGCACTGCCCTGGGCAG
AGGGTACG

GGCGGGCAGCCCAGGCCAGCAGCAGGGCCTTCGGGTTGGGTTTGACCATTCAAACTAACGC
CTTCATTTC

TCTCCCATGCTTTCTCCTCCGGCCAGGAGCGGAAGGTACATGCCCTTTCTGCTATCTTCGCTTCT
TAGCG

CTTTTGAGTTGTGTGCGTGTGGATTCTTCTGTCCTCTCTTTGGGAAATAGACTTTCTTTGTGTGGGTG
GG

GCTGTAGCTGGGAGAGTGTGCTGTGTGGGGCAGGGGTGGAGCCTGTGGGCCTTACCCCGGC
CTCCCTCCC

TCTCTTGCAGGTTTTCATAGAGCTGAATCACATTAAAAAGTGCAATACAGTTCGAGGCGTCTTTGTC
CTG

GAGGAATTTGGTAATTACACTATTTTGCTCTTAGGTCTGGACTCACATGGCAGTAACTCAAACCTC
GGAG

CTCCAGAGGAGGGTCTAGGGGCAGGGAGAAGAAGAACCTCTGTAGAGAAGTCAGGAGGAGCA
GGAGTGAC

AAGGAAGAAAAGGGACCCCTGAGATGAGAGCCGGGATGTGGAAGGGAAAGATAGATAATGGAT
CGCAGAA

GAGCAAATGGGGCCTCAGGTGGGTTTCGAGTTTAGAGGTGGTCCTGAAGCAGCTCAGTGAAGT
AGCACCA

CCTCCCCGTGCTGGGCCAGCCAGTGGTCAGAAAGTTTGGGACACAGCAGACTGGCGACCCC
CAAGCTCCA

CTATGTCCATGTCACCTGCTGCAATCTCTCTAGCAGCCCAGTGCGCTAGCCAGCCTCCCTGGC
GCCGCTC

TGGTCTCCACCTGGCCTCTGTGCCAGACAAAGGGGCACCGGGCGTCGCAGGCAGACTCACT
TGAGGGACA

GAGGAGTCTAGCGGGACAGCTGTAGCCGGAAGCTGGGAGCCAGCGCTAGGGAGTGGTGTAGT
GGGTTCTC

ACCCTGGGGCTTTGAGTTCCTGCTGTGTTTGTGCTTCATTCTTTTGTGCCCCTCATCGCTGCTATG
AAAA

CCTTCGTTCTCCAGCAGGTAAAGTGACCTCGGCTCGTGCTGCCTCTCCCCAGGTCTCTGCCTT
GGGCCCT

GCCCTGGGTGGGTAGCTCCCATCGAACCAGCTCCTCACACTGCCAGCTCGTCTCTTCCTTTCT
CCCCTAC

CCTCTTTCTTCCTACCTTGCCTCCCCCTTCTCACCGGCTCACTGGACTTTCCTCCCCGTGTGTTC
CCTTC

TCTCTTCTGTCAGAGAGATACTTGGGGGAATCTTCCAACTCGGGAGAATGGCCTGGGGCTTCTT
GCTCTA

CCTTTGATGAGTTTATTCCCTGCTGACCCTGGGTTCCCCCAAATTCTACCTCCCAGGGTCAGAAG
AAGGT

ACGGCCAATTCTCCTCCTGCCTTTTCTGGCCCCCAGGGAGATGCCAGGCCCCTCTGCTCCCA
GTCTCAAG

GGGCCATGATGGTGGCTGAGCCTGGGGGGCCTACTCCTTCCTCTGGCCAAGCAGAACCCCTG
CATGGCTG

GAGCGGGGGGACTCTCACTCTTGCCTTCTGGGCTGTGGACATGTGTGTATTTTGCCTGATGCTC
CCTGTC

ACCTCCCTCCCTGGGTGCTGTACTCGCCTGTCTTCTGCTGGGTGTGTTGCTCCAGAGCCCTCAG
ACGCTC

TCCCTCACACAGGCAGGTCTGGGCTTCTCCTGCCACTCAGGGAAGGACACTGCCACATCCTGT
GGGTTTA

TATCATGTTACCTCTTCATCCAGCCTCAAGACCCTCTGTGTAGGGGGCTGGGAATAACGGGAACT
GTGTT

TAACATGACCCAATTTTCAGGCTGGTGACGAGTTCACGGGTGTCTCAGCAAAGTTGGACACAAG
CTAGAG

AGAGCTCTCTCCTCCCAGCCAAAACCTGGGAATTCCCCACTGCAGCTCCTCAGGGGCCCTGG
ACAGTAGC

TGTGACCAGGCTGGTGAAAGGCCCGTCCCTCCCATCCAGGGCTTTCAAAGAAGTGGGTAGAAT
CACGGCC

ATCAAGTAAACGCCACTGCGGATGACAGAGGCCTAGCTGAGGAGGCTAGGGCCATGTCCTCC
CAGCTGGC

AGAGTCTGGACAGAGCCTCTGGTGGCCTCTGCCCTGTCATAGCCTAAGCTGCTGCAGCCACTG
TGAGAGG

CCACAGAGGGGAAGGGGACCCTGACGCCGGGCGCTGCCGCCTGGGGGAGCATGGCACGA
TTGCTCTTAGT

GTTGAACTTTCTGTTTTGTCTCTTGCCCGTCCGGTTTTAGTTCCTGAAATTAAAGAAGTGGTGAGCC
ACA

AGTACAAGACACCAATGGTGAGTGTGCCGCTCAACCCTGATGGGCCACGGTGCGCAGGGCTC
CCTGGGGG

ACCTTCGGACAGGAGGAGGGGAGGGCCCTCTGAGCGCACAGGGGTGAGTGGGGACCTCGTT
TTGTTGCCA

AAGGTTCAACTCAGGCTGAAACAGGTCTTGAGCCCTGAGAGACAGACACACACACACGGATCC
CCTCTCC

CATGGGGACAGCTTCCTTGCTTGGAGCTCACGTTTAGCCCAGAGGCCCATGCCCAGTGTTAGG
GCAGGAG

TTGCCCATTGGCAGATCCTTCACATATGGAGATGGAGGAGTCTCAGTATCGTGATCCGCCTGGTT
TTCTC

TCCTCTAGGCCCACGAAATCTGCTACTCCGTATTATGTCTCTTCTCGTACGTGGCTGCAGTTCATA
GCAG

TGAGGAAGATCTCAGAACCCCGCCCCGGCCTGTCTCTAGCTGATGGAGAGGGGCTACGCAGC
TGCCCCAG

CCCAGGGCACGCCCCTGGCCCCTTGCTGTTCCCAAGTGCACGATGCTGCTGTGACTGAGGAG
TGGATGAT

GCTCGTGTGTCCTCTGCAAGCCCCCTGCTGTGGCTTGGTTGGTTACCGGTTATGTGTCCCTCTG
AGTGTG

TCTTGAGCGTGTCCACCTTCTCCCTCTCCACTCCCAGAAGACCAAACTGCCTTCCCCTCAGGG
CTCAAGA

ATGTGTACAGTCTGTGGGGCCGGTGTGAACCCACTATTTTGTGTCCTTGAGACATTTGTGTTGTGG
TTCC

TTGTCCTTGTCCCTGGCGTTATAACTGTCCACTGCAAGAGTCTGGCTCTCCCTTCTCTGTGACCC
GGCAT

GACTGGGCGCCTGGAGCAGTTTCACTCTGTGAGGAGTGAGGGAACCCTGGGGCTCACCCTCT
CAGAGGAA

GGGCACAGAGAGGAAGGGAAGAATTGGGGGGCAGCCGGAGTGAGTGGCAGCCTCCCTGCTT
CCTTCTGCA

TTCCCAAGCCGGCAGCTACTGCCCAGGGCCCGCAGTGTTGGCTGCTGCCTGCCACAGCCTC
TGTGACTGC

AGTGGAGCGGCGAATTCCCTGTGGCCTGCCATGCCTTCGGCATCAGAGGATGGAGTGGTCGA
GGCTAGTG

GAGTCCCAGGGACCGCTGGCTGCTCTGCCTGAGCATCAGGGAGGGGGCAGGAAAGACCAAG
CTGGGTTTG

CACATCTGTCTGCAGGCTGTCTCTCCAGGCACGGGGTGTCAGGAGGGAGAGACAGCCTGGGT
ATGGGCAA

GAAATGACTGTAAATATTTCAGCCCCACATTATTTATAGAAAATGTACAGTTGTGTGAATGTGAAATA
AA

TGTCCTCAACTCCC

http://useast.ensembl.org/Homo_sapiens/Transcript/Exons?db=core;g=ENSG0000011051
4;r=11:472691

61-47330031;t=ENST00000706887

Exon: 3; -ENSE00000839266; Start-47,274,563; End -47,275,159; Length -597

GCACCCGAGCAGTGATAGCGTGGCCCAGACTCCTGAATTGCTACGGCGATACCCCTTGGA

GGATCACACTGAGTTTCCCCTGCCCCCAGATGTAGTGTTCTTCTGCCAGCCCGAGGGCTG

CCTGAGCGTGCGGCAGCGGCGCATGAGCCTTCGGGATGATACCTCTTTTGTCTTCACCCT

CACTGACAAGGACACTGGAGTCACGCGATATGGCATCTGTGTTAACTTCTACCGCTCCTT

CCAAAAGCGAATCTCTAAGGAGAAGGGGGAAGGTGGGGCAGGGTCCCGTGGGAAGGAAGG

AACCCATGCCACCTGTGCCTCAGAAGAGGGTGGCACTGAGAGCTCAGAGAGTGGCTCATC

CCTGCAGCCTCTCAGTGCTGACTCTACCCCTGATGTGAACCAGTCTCCTCGGGGCAAACG

CCGGGCCAAGGCGGGGAGCCGCTCCCGCAACAGTACTCTCACGTCCCTGTGCGTGCTCAG

CCACTACCCTTTCTTCTCCACCTTCCGAGAGTGTTTGTATACTCTCAAGCGCCTGGTGGA

CTGCTGTAGTGAGCGCCTTCTGGGCAAGAAACTGGGCATCCCTCGAGGCGTACAAAG
